# Dysregulation of Pseudogenes/lncRNA-Hsa-miR-1-3p-PAICS Pathway Promotes the Development of NSCLC

**DOI:** 10.1155/2022/4714931

**Published:** 2022-08-30

**Authors:** Yichen Song, Zhiying Wang, Lewei He, Feidi Sun, Beilei Zhang, Fu Wang

**Affiliations:** ^1^School of Basic Medical Sciences, Xi'an Jiaotong University, Xi'an 710061, China; ^2^Department of Obstetrics and Gynecology, Tangdu Hospital, Air Force Medical University, Xi'an, China; ^3^Xianyang Key Laboratory of Molecular Imaging and Drug Synthesis, School of Pharmacy, Shaanxi Institute of International Trade & Commerce, Xianyang 712046, China

## Abstract

**Objective:**

Non-small cell lung cancer (NSCLC) explains about 80 percent of whole lung cancers, and its 5-year survival rate is impoverished, as when people are first diagnosed, 68% of whom are identified at a dangerous stage. The molecular mechanisms of NSCLC are still being explored.

**Methods:**

GSE18842 and GSE19804 were exerted to scan for diversely expressed genes (DEGs) in NSCLC, and then we used GEPIA for the validation of DEGs expression. The prognostic values were determined through Kaplan–Meier analysis. Three target prediction databases indicated potential microRNAs (miRNAs), while miRNet predicted hsa-miR-1-3p′s upstream long non-coding RNAs (lncRNAs) and pseudogenes. UALCAN was utilized to identify the co-expressed genes of PAICS, while enrichment analysis on them was managed with Enrichr.

**Results:**

We initially found that the gene expression level of cyclin B1 (CCNB1), cyclin-dependent kinases1 (CDK1), and phosphoribosylaminoimidazole succinocarboxamide synthetase (PAICS) had a notable increase in NSCLC. We predicted 6, 10, and 7 microRNAs to target CCNB1, CDK1, and PAICS, respectively. Among miRNA-mRNA (microRNA-messenger RNA) pairs, we deduced that the hsa-miR-1-PAICS axis was the most potential one to inhibit the occurrence of NSCLC. We also noted that the hsa-miR-1-3p-PAICS axis participated in regulating the process of mitosis with mechanical functions. Moreover, we identified 5 pseudogenes and 33 long non-coding RNAs (lncRNAs) that might inhibit the hsa-miR-1-3p-PAICS axis in NSCLC.

**Conclusions:**

The pseudogene/lncRNA-hsa-miR-1-3p-PAICS is very important in NSCLC on the basis of this study, thus providing us with effective treatments and promising biomarkers for the diagnosis of NSCLC.

## 1. Introduction

So far, there are many virulent tumors, especially NSCLC, which mainly explains 80% of cases in China. It is worth noting that NSCLC contains many subforms, such as squamous cell carcinoma and adenocarcinoma. It is reported that about 68% of patients are found at a hazardous stage with a low five-year survival rate [1]. Surgery [2], radiation [3], chemotherapy [4], biotherapy [5], immunotherapy [6], and electric field therapy [7] are the current treatment options for NSCLC. However, the therapeutic result remains poor with the usage of several treatment procedures. The primary reason is that the pathophysiology of the disease and its prognostic markers remain unclear.

There is a non-coding RNA, whose length is beyond 200 nucleotides, called lncRNA. Numerous studies have established that lncRNA has a noteworthy function in manifold biological processes, including dosage compensation [8], epigenetic control [9], cell cycle regulation [10], and cell differentiation regulation [11], which has been a focus of genetic study. Generally, lncRNA transcripts can influence the activity of particular proteins by chemically linking to them. To regulate other RNA transcripts, competing endogenous RNAs (ceRNAs) can strive for shared miRNAs. A non-coding pseudogene can attach to and compete with the same collection of miRNAs via microRNA response elements (MREs) as a combination zone [12], affecting the distribution of miRNA molecules on all their target miRNAs. It has been proved that pseudogenes are a convincing example of ceRNA as they presumably include many of the same MREs as their ancestor genes and can operate to combine with the target miRNAs [13]. Furthermore, ceRNA may suppress the activity of some miRNAs [14], whose decreased expression may lead to overexpression of particular genes associated with NSCLC.

**Figure 1:**
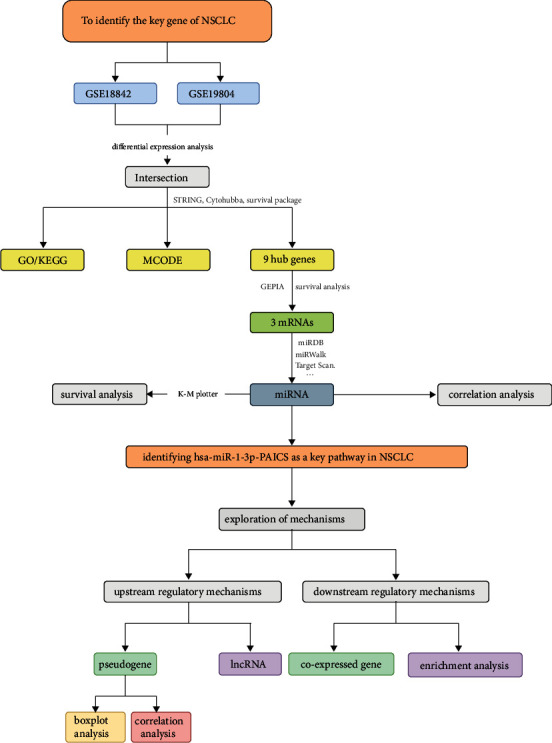
The process of this study.

Through a variety of analytical processes, we created a network connected with the evolution of NSCLC in this study. We are sure that this research will bring new methods to the fields of treatment and pathogenesis of NSCLC. According to Figure 1, you would have a good understanding of our research process.

## 2. Materials and Methods

### 2.1. The Analysis of Microarray Data and Scanning for Diversely Expressed Genes

Aiming at comparing genome-wide gene expression of NSCLC with normal tissues, we searched the widely utilized GEO database (https://www.ncbi.nlm.nih.gov/geo/) [15]. For future studies, the GSE18842 dataset (46 tumor and 45 normal samples) and the GSE19804 dataset (60 tumor and 60 normal samples) were used. We filtered the DEGs on GSE18842 and GSE19804 microarray, respectively, using the R program limma [16] with the condition that *p* value is less than 0.05, log2FC is greater than 2. After that, We intersected the results to obtain the common DEGs thus drawing a Venn diagram.

### 2.2. The Analysis of Functional Enrichment, Interplay Network, and the Recognition of Hub Gene

In order to further elucidate the dormant functional annotation and pathway enrichment-related with the DEGs [17], Gene Ontology (GO) analysis, was conducted using the clusterProfiler package (version: 3.18.0) [18], and *p* < 0.05 indicates statistically remarkable variances. The network of DEGs' protein-protein interactions (PPIs) was made through STRING (version: 11.0) [19], and the threshold score was 0.4. We deleted protein nodes that did not have a relationship with other proteins. Additionally, the PPI network was examined by Cytoscape (version 3.8.0) software to recognize key modules and hub genes (which is shown in text foot notation 8) (version 3.7.2) [20]. We also use the MCODE (version: 2.0.0) plugin to identify important clustering modules on the foundation of the following criteria: the score of MCODE >10 and node count >20, and by using the clusterProfiler software [21], the genes' pathway enrichment analysis included in these modules was conducted. Following that, we used the CytoHubba (0.1) plugin to scan for the PPI network and identified genes with a degree greater than 30 as NSCLC hub genes [22].

### 2.3. The Survival Analysis and Confirmation

To thoroughly assess hub genes' prognostic relevance in NSCLC, we use the survival software (version 3.2-7) for survival analyses, with the default settings and the median as the cut-off value [23]. The sample of NSCLC was picked to be the dataset. Besides, the Cox proportional hazards and Kaplan-Meier models were exerted to compute hazard ratio (HR). *p* < 0.05 means the dissimilarity is statistically marked. GEPIA database (http://gepia.cancer-pku.cn/detail.php) [24], which is to examine the expression data from RNA sequencing, contains data from 483 cancers and 347 normal samples from the TCGA and GTEx projects' RNA sequencing programs. To assess the above gene survival through this database, the Group Cutoff to Quartile, the Cutoff-High to 75%, the Cutoff-Low to 25%, and the 95% Confidence Interval to NO were set. Betwixt tumor and normal samples, to study the differential expression and to carry out differential expression analysis simultaneously, we set all parameters to default values. *p* < 0.05 means the dissimilarity is statistically notable.

### 2.4. The Prediction of miRNA of Hub Genes

A rather complete approach to microRNA (miRNA) prediction was conducted in this investigation. TargetScan (https://www.targetscan.org/) [25], miRmap (https://mirmap.ezlab.org/) [26], miRDB (http://mirdb.org) [27, 28], PITA (https://genie.weizmann.ac.il/pubs/mir07/mir07_dyn_data.html) [29], microT (http://www.microrna.gr/webServer) [30, 31], RNA22 (https://cm.jefferson.edu/rna22/) [32], miRWalk (http://mirwalk.umm.uni-heidelberg.de/) [33], miRanda (http://www.microrna.org/microrna/home.do) [34]. Only the miRNAs that were listed in several prediction systems were picked for further investigation.

### 2.5. Screening for Key miRNAs

We used Cytoscape to generate three target gene networks in this article [20]. Following that, the predictive importance of miRNA expression of hub genes that were found in NSCLC was determined through the Kaplan-Meier plotter (https://kmplot.com/) [35], a web-based database for gene expression. The data of this database contains information about lung cancer [36], ovarian cancer [37], gastric cancer [38], and breast cancer [39].

To summarize, miRNAs were initially taken as input. Based on the median expression value, the complete amount cases of NSCLC were categorized into a lower expressed classification and a higher expressed classification. Then we conducted Kaplan–Meier survival charts with the use of this web page. Additionally, we generated and published the HR, 95% CI, and logrank *p*-value on the homepage automatically. *p*-value <0.05 denotes statistically notable.

### 2.6. ENCORI Database Analysis

The ENCORI database (https://starbase.sysu.edu.cn) is a free platform to research the interactions of non-coding RNAs [40, 41]. We exerted ENCORI to assess the bond betwixt miRNAs and genes or pseudogenes expression and *R* -0.1 and *p*-value of 0.05 were found to be the cut-off values for associated miRNA-gene/pseudogene pairings. Additionally, the ENCORI database was applied to foretell pseudogenes and lncRNAs that possibly tie to hsa-miR-1-3p.

### 2.7. UALCAN Database Analysis

UALCAN (http://ualcan.path.uab.edu) is a database to evaluate the diverse expression genes and survival effects that enables simple entry to publicly accessible cancer transcriptome data, NSCLC [42, 43] included. The database work to identify PAICS co-expressed genes in NSCLC in this investigation. Then, as noted previously, these co-expressed genes were cross-referenced with those obtained from the GEPIA database. The co-expressed genes that were shown frequently in both databases were reclassified as co-expressed genes and we selected them to perform further enrichment analysis.

### 2.8. Enrichr Database Analysis

As previously published [44], we performed functional annotation and KEGG pathway enrichment analysis on the co-expressed genes of PAICS via the Enrichr database (https://maayanlab.cloud/Enrichr/). There were three classifications (BP, CC, and MF) in the GO functional annotation. On the homepage, the first five enriched GO terms and KEGG pathways were shown and downloaded as pictures.

### 2.9. Identification of lncRNAs Upstream of miRNA

To anticipate the regulator of hsa-miR-1-3p, we use LncACTdb 2.0 (http://www.bio-bigdata.net/LncACTdb/) [45], which contains extensive details on ceRNAs in various species and disorders. Similarly, so as to foretell possible lncRNAs that were associated with hsa-miR-1-3p, we used miRNet (https://www.mirnet.ca/) [46], a comprehensive platform that combined data from numerous miRNA-related databases (TarBase, miRTarBase, miRecords, and miRanda). We utilized ENCORI [40], miRNet [46], and LncACTdb [45, 47] to predict the lncRNA upstream of hsa-miR-1-3p. By combining the data, we achieved the maximum number of possible lncRNAs for hsa-miR-1-3p.

## 3. Results

### 3.1. Identification of DEGs

**Figure 2:**
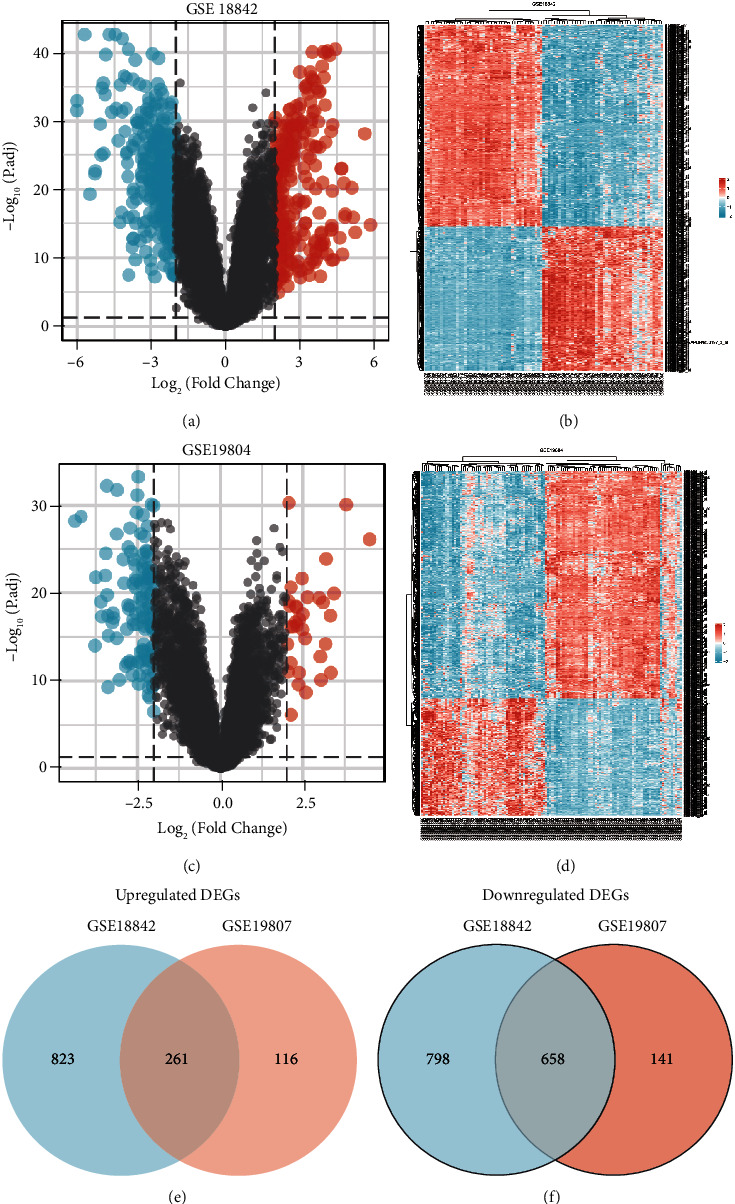
Scanning of DEGs. (a, b) Volcano result and heat map result of the GSE18842. (c, d) Volcano result and heat map result of GSE19804. (e, f) Up-regulated and down-regulated DEGs in GSE18842 and GSE19804.

The GSE18842 dataset consists of 2540 DEGs, 1084 of which are up-regulated and 1456 of which are down-regulated (Supplementary Table S1). 1136 DEGs were identified by the GSE19804, comprising 337 highly expressed genes and 799 low expressed genes. Venn graphs were constructed for two sets of DEGs by a bioinformatics and evolutionary genomics online application. Finally, we classified 919 DEGs, comprising 261 UP and 658 DOWN genes (Figures 2(a)–2(f)).

### 3.2. Dinucleotides' Functional Enrichment Analysis, Integration of Networks which Show Proteins Interact with Each Other, and Analysis Modular

**Figure 3:**
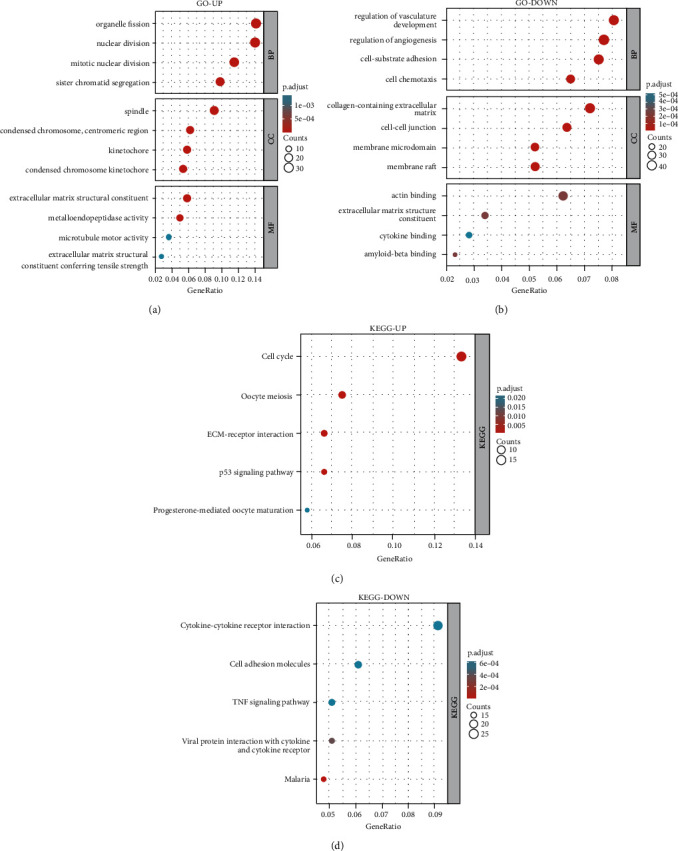
Co-expressed genes were analyzed in GO pathways and KEGG pathways. (a, b) DEGs' GO enrichment analysis. (c, d) DEGs' KEGG pathway analysis.

Intending to gain a better comprehension of DEGs' biological roles, GO enrichment analysis was performed. The BP category enriched for overexpressed DEGs involved in the division of the organelles, the nucleus, and the mitotic division of the nucleus (Figure 3(a)). By contrast, the DEGs of down-regulation are abundant in controlling vasculature development, regulation of angiogenesis, and cell-substrate adhesion (Figure 3(b)). The increased DEGs in the CC category are mostly localized in the spindle and condensed chromosome (Figure 3(a)). Down-regulated DEGs were commonly placed in the collagen-containing extracellular matrix and cell-cell junction (Figure 3(b)). Up-regulated genes are primarily concentrated in extracellular matrix structural constituents and metalloendopeptidase activity in the MF category. The down-regulated DEGs are mostly actin binding, extracellular matrix structural components, and cytokine binding (Figure 3(b)). Furthermore, revealed by KEGG pathway analysis, what was considered highly expressed in cell cycle, Oocyte meiosis, and ECM-receptor interaction were up-regulated DEGs (Figure 3(c)). In comparison, down-regulated DEGs are much more frequent in Cytokine-cytokine receptor interaction, as well as Cell adhesion molecules (Figure 3(d)).

**Figure 4:**
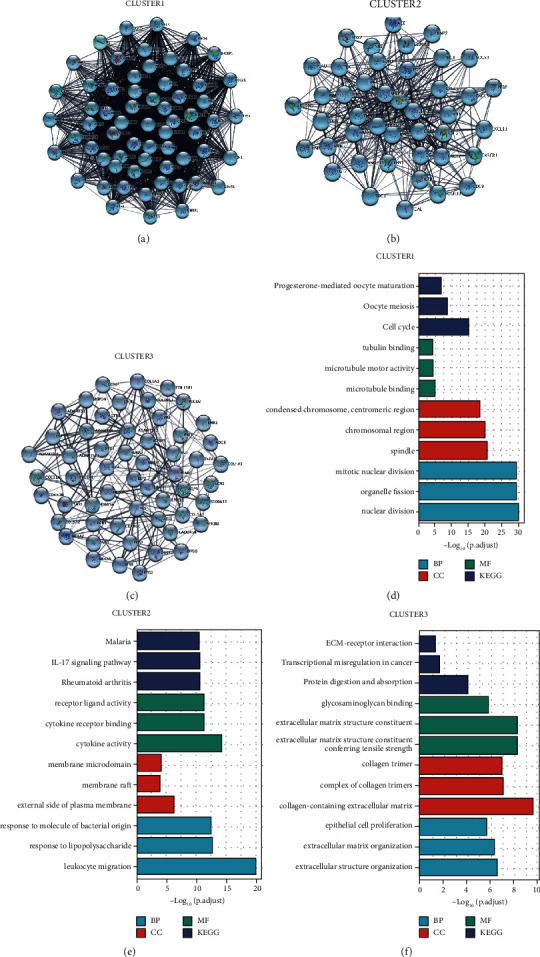
The analysis of MCODE in generic DEGs. (a b, c) The three important modules. (d, e, f) The three important modules pathway enrichment analyses.

Next, we constructed the PPI system through STRING and evaluated it through the Cytoscape program. We used the MCODE plugin and obtained three major clustering modules and examined the functional annotation's degree for these modules (Figure 4). The first cluster module contains 63 nodes and 1777 edges. Module 1 genes are primarily involved in progesterone-mediated oocyte maturation (Figures 4(a), 4(d)). Module 2 of the cluster consists of 43 nodes and 427 edges. Module 2 contains genes mainly involved in malaria and the interleukin 17 (IL-17) pathway (Figures 4(b), 4(e)). The third cluster module contains 55 nodes and 199 edges. In the module, the primary genes are related to extracellular matrix-receptor (ECM-receptor) interaction, transcription dysregulation in cancer, and protein digestion and absorption (Figures 4(c), 4(f)). In total, 125 DEGs were identified using the degree method in the CytoHubba plugin for further research.

### 3.3. Survival Analysis and Validation

**Figure 5:**
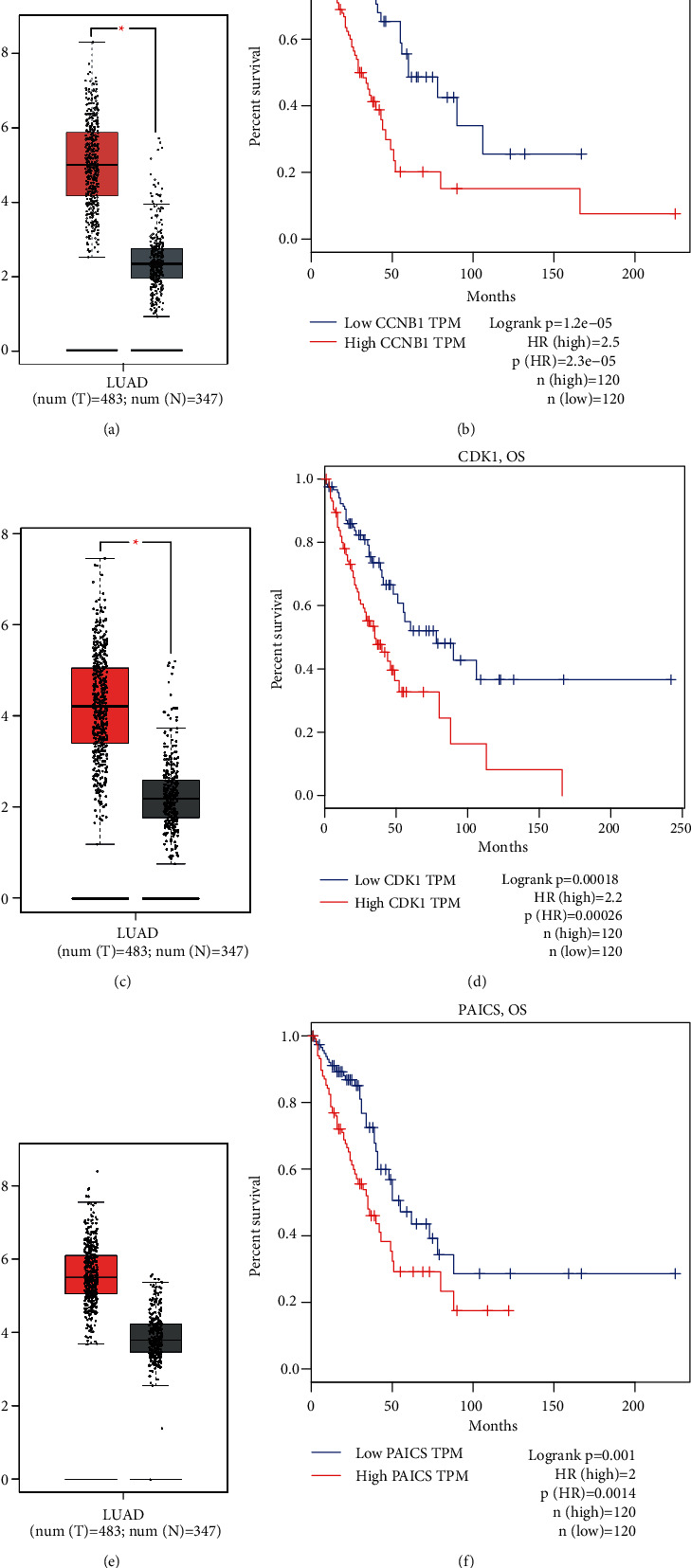
All DEGs' differential expression and Survival analysis results. Differential expression and survival analyses were performed through the GEPIA database. The figures manifested that all three genes were diversely expressed in normal samples as well as tumor samples, which cut patients' OS rate. (a-b) The distinguishing expression and survival analysis of CCNB1. (c-d) The differential expression and survival analysis of CDK1. (e-f) The differential expression and survival analysis of PAICS.

The predictive significance of 125 important genes was assessed through the r.survival program. Examination of survival data proved most genes were not related to overall survival (OS) in NSCLC patients. But Cox proportional risk suggested that EZH2, CCNB1, MMP9, SOX2, FCGR3B, IL6, COL1A1, PAICS, and CDK1 were substantially linked with the operating system in NSCLC patients (Table 1). Three genes (CCNB1, CDK1, and PAICS) have been shown to have a fairly significant effect on patients' OS rates, and the tumor and normal groups' differences is statistically significant (Figures 5(a)–5(f)). Overall, CCNB1, CDK1, and PAICS could be three critical genes that influence tumor stage development of NSCLC and they could produce a poor prognosis.

### 3.4. hsa-miR-1-3p-PAICS Axis is Picked out to be a Potential Pathway which is Linked to the Evolution of NSCLC

**Figure 6:**
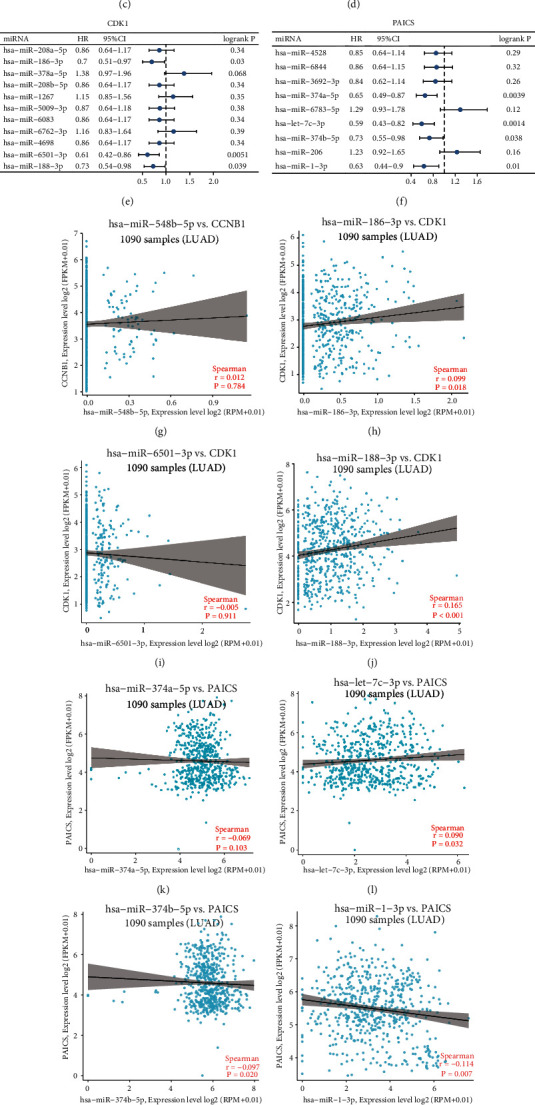
Identification of upstream potential miRNAs of CCNB1, CDK1 and PAICS. (a-c) The miRNA-CCNB1 network, miRNA-CDK1 network, and miRNA-PAICS network that were constructed by Cytoscape. (d-f) Potential upstream miRNAs' prognostic values of CCNB1, CDK1, PAICS in NSCLC. (g-n) Expression correlation of hsa-miR-548b-5p and CCNB1, hsa-miR-188-3p and CDK1, hsa-miR-6501-3p and CDK1, hsa-miR-188-3p and CDK1, hsa-miR-374a-5p and PAICS, hsa-let-7c-3p and PAIC, hsa-miR-374b-5p and PAICS, hsa-miR-1-3p and PAICS in NSCLC. (o) Diverse expression of hsa-miR-1-3p based on GEPIA database.

MiRNAs chiefly functioned in negative gene regulation and are important in human biological processes, cancer initiation and progression included. As a result, we used eight prediction programs to determine the upstream miRNAs of CCNB1, CDK1, and PAICS (Table 2). Lastly, we discovered 6, 10, and 7 upstream miRNAs which may, respectively, target CCNB1, CDK1, and PAICS. To facilitate visualization, miRNA-CCNB1, miRNA-CDK1, and miRNA-PAICS subnetworks were constructed, as seen in Figures 6(a)–6(c). The predictive significance of these miRNAs in NSCLC was then determined by the TCGA database. As seen in Figure 6(d), among all predicted CCNB1 miRNAs, elevated appearance of hsa-miR-548b-5p is in connection with a favorable OS rate in NSCLC patients, whereas highly expressed hsa-miR-3130-5p is in connection with a bad OS in NSCLC patients. Higher expression of hsa-miR-6501-3p, hsa-miR-188-3p, and hsa-miR-186-3p in CDK1 was, respectively, connected to a favorable prognosis (Figure 6(e)). In PAICS, upregulation of hsa-miR-374a-5p and hsa-miR-1-3p, respectively, corresponded to favorable prognosis. Given the functional mechanism and carcinogenic potential of CCNB1, CDK1, and PAICS miRNAs, these three genes' upstream miRNAs ought to be tumor suppressive. As a result, we chose hsa-miR-548b-5p, hsa-miR-186-3p, hsa-miR-6501-3p, hsa-miR-188-3p, hsa-miR-374a-5p, hsa-let-7c-3p, hsa-miR-374b-5p and hsa-miR-1-3p for further research of miRNA-mRNA pair expression relationships. There is a strong negative correlation only in hsa-miR-1-3p with PAICS in NSCLC, as seen in Figures 6(g)–6(n). At the same time, we exerted GEPIA database to judge hsa-miR-1-3p′ expression difference, and the consequence was that its expression was notably decreased in patients in comparison with the normal, proving that the research of the miRNA has clinical significance (Figure 6(o)). In summary, the most plausible pathway mediating the staging progression of NSCLC ought to be the hsa-miR-1-3p-PAICS axis.

### 3.5. The hsa-miR-1-3p-PAICS Axis is Related to the Regulation of Mitosis Revealed by Co-Expression and Enrichment Analyses

**Figure 7:**
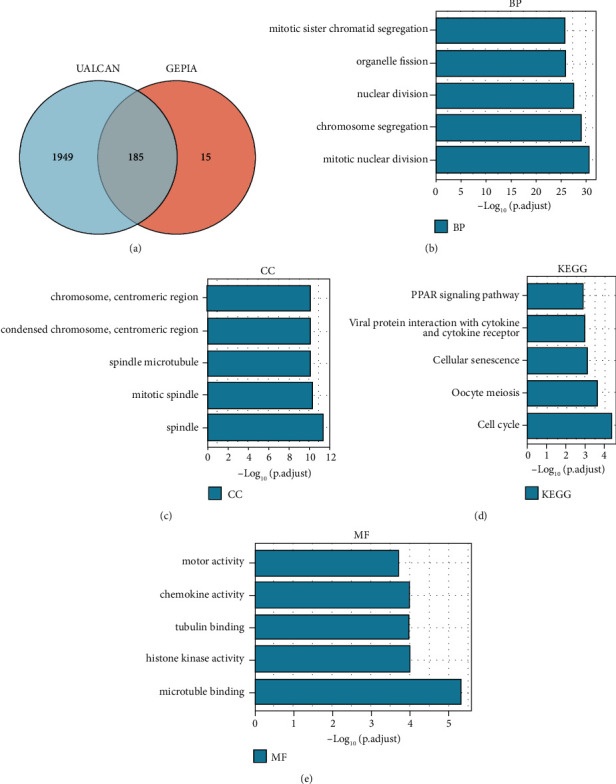
Results of co-expressed genes' enrichment analysis of PAICS in NSCLC. (a) The co-expressed genes of PAICS both in UALCAN and GEPIA databases. (b) First five enriched BP items. (c) First five enriched CC items. (d) First five enriched MF items. (e) First five enriched KEGG items.

Two datasets were utilized for co-expression analysis: UALCAN and GEPIA. We obtained, respectively, 1898 and 200 (the top 200 most influential genes) co-expressed genes from the two database, and Supplementary Table S2 itemized them. We discovered that 185 co-expressed PAICS genes were frequent in both databases (Figure 7(a), Table 3). These genes were subjected to GO functional annotation and KEGG pathway enrichment analysis using the enrichment of the Enrichr database. Mitotic sister chromatid segregation and organelle fission are included in the BP class (Figure 7(b)). The CC class encompasses chromosome and centromeric regions (Figure 7(c)), whereas the MF class has motor activity and chemokine activity (Figure 7(e)). KEGG pathways that have been enriched mainly indicate the PPAR signaling pathway (Figure 7(d)). These results indicate that by controlling the chromosome and centromeric region the hsa-miR-1-3p-PAICS axis may be implicated in mitotic sister chromatid segregation, PPAR signaling pathway, and motor activity, thus limiting the development of NSCLC.

### 3.6. Hsa-miR-1-3p-PAICS's Upstream Dormant Pseudogenes and lncRNAs

**Figure 8:**
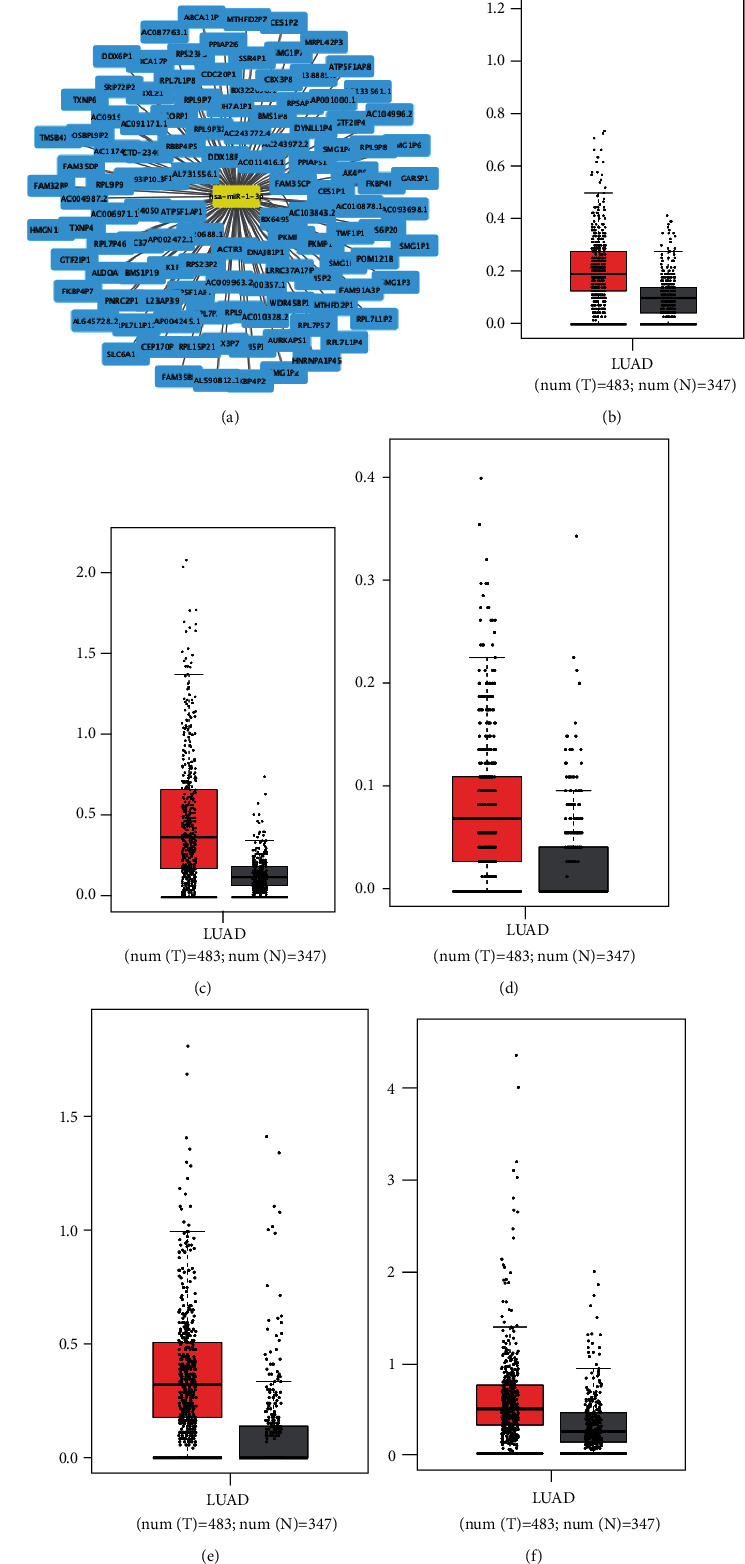
Hsa-miR-1-3p′s upstream dormant pseudogenes. (a) Pseudogenes-hsa-miR-1-3p axis The expression degrees of FAM91A3P (b) LRRC37A6P (c)、PKMP1 (d)、RPL9P32 (e)、BMS1P8 (f) in NSCLC in comparison to normal controls. “^*∗*^” means “*p*-value < 0.05”.

**Figure 9:**
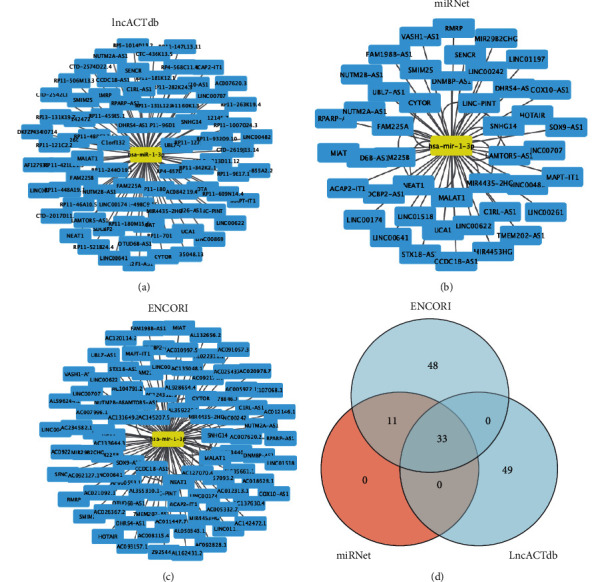
Hsa-miR-1-3p′s dormant upstream lncRNAs. (a-c) Dormant lncRNAs foretold by lncACTdb, miRNet, and ENCORI. (d) 3 intersected lncRNAs from lncACTdb, miRNet, and ENCORI databases.

**Figure 10:**
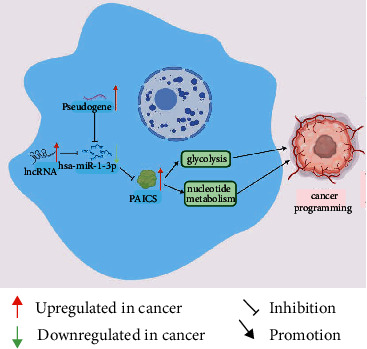
The pseudogene/lncRNA-hsa-miR-1-3p-PAICS network model and its expression and dormant influences on NSCLC.

Pseudogenes and lncRNAs both are significant subtypes of non-coding RNAs, whose main function is interacting with mRNA as competing endogenous RNAs by competing for common miRNAs. As a result, we used the ENCORI database to anticipate dormant pseudogenes upstream of hsa-miR-1-3p-PAICS. In Figure 8(a), 119 pseudogenes were identified. These pseudogenes should be oncogenes in NSCLC on the foundation of the ceRNA mechanism. We exerted GEPIA to determine 119 pseudogenes' expression degrees. Finally, only five pseudogenes were substantially elevated in the part with cancer compared to normal controls: FAM91A3P (shown in Figure 8(b)), LRRC37A6P (shown in Figure 8(c)) lly, we predicted certain lncRNAs that would influence hsa-miR-1-3p (Figure 9, Supplementary Table S3). As shown in Figures 9(a)–9(c), 82, 44, and 92 upstream lncRNAs were, respectively, discovered in lncACTdb, miRNet, and ENCORI. Supplementary Table S3 has detailed lncRNAs. Through the intersection of the three databases, 33 lncRNAs are constructed (Figure 9(d)). In summary, overexpression of lncRNAs/pseudogenes results in enhanced PAICS expression and mitosis regulation, which contributes to the development of NSCLC (Figure 10).

## 4. Discussion

NSCLC grow and divide slowly in comparison to small cell lung cancer cells and disseminate relatively late. NSCLC accounts for around 80% of all lung malignancies [48], approximately 68% of which are diagnosed at a late stage with a poor 5-year survival rate [1]. It is essential to comprehend the molecular process of NSCLC advancement to create innovative therapeutic strategies and improve patients' survival rates.

With bioinformatics technology being introduced into medical molecular biology [49], the scope of basic research can be expanded, and the prediction of important biomarkers can be more convenient and accurate. Furthermore, it's through the bioinformatics methods that comprehensive exploration and analysis of mRNA data sets [50], miRNA data sets [51], and lncRNA data sets [52] in different databases can be conducted, which eventually improves the accuracy of differentially expressed genes determination. This study screened three genes with research values from 919 DEGs. Then, the main idea of this study was to construct the regulatory axis of ceRNA, and to predict the potential miRNAs, lncRNAs, and regulated upstream of central genes through the data set. Finally, the regulatory axis hsa-miR-1-3p-PAICS is constructed.

Through analysis and survival analysis, this study identified three genes (CCNB1, CDK1, and PAICS) as key genes linked with the development of NSCLC in this study. CCNB1, CDK1, and PAICS expression levels raised in NSCLC, which have been implicated in the development of many human malignancies as oncogenes. What's more, we can indicate that three genes potentially function as biomarkers for cancer from previous studies. For instance, the high-level mRNA expression of CCNB1 and CENPF can be regulated by hnRNPR, thus promoting the aggressiveness of gastric cancer [53]; Zhang et al. [54] suggested that high expression of CCNB1 in pancreatic cancer inhibits cell proliferation and promotes cell senescence through p53 pathway; Sepideh lzadi [55] found that CDK1 is an important target for breast cancer diagnosis and treatment; Huang et al. [56] indicated that the interaction between CDK1 and SOX2 promotes the dryness of the cells in lung cancer; the study of Shuyi Zhou [57] suggested that PAICS may provide us a novel treatment for lung adenocarcinoma; Moloy Goswami et al. [58] confirmed that increased expression of PPAT and PAICS affects disease progression by regulating lung adenocarcinoma metabolism. From all the reports and our analytic results, we can draw the conclusion that CCNB1, CDK1, and PAICS may be three hub oncogenes in the development of NSCLC.

MiRNAs are non-coding RNA molecules that are involved in controlling biological activity by downregulating the expression of target genes [59]. So we intend to identify miRNAs that specifically target CCNB1, CDK1, and PAICS. Numerous miRNAs were predicted through a variety of online sources, including six for CCNB1, ten for CDK1, and seven for PAICS. The miRNAs mentioned above function as tumor suppressor miRNAs in NSCLC on the foundation of their mode of action. Following survival analysis, we picked eight sets of miRNA-mRNAs as expressions for subsequent correlation study. Connection analysis revealed a strong negative correlation only in the hsa-miR-1-3p-PAICS pair. In conclusion, the hsa-miR-1-3p-PAICS axis is being investigated as a possible route implicated in the development of NSCLC. Numerous studies have established that hsa-miR-1-3p is a critical inhibitor of the genesis and progression of a range of human malignancies. For example, the study of Zhanrui Mao [60] showed that the low level of hsa-miR-1-3p may be a indication of CR which had a significant relationship with the disease stage according to the analysis of miRNA data in TCGA; Li et al. [61] suggested that the apoptosis and proliferation of the cells in hepatoma can be influenced by the overregulation of hsa-miR-1-3p. Afterward, we identify the co-expressed genes of PAICS. The GO analysis revealed a high enrichment of these co-expressed genes during mitosis. Consequently, by regulating the mitosis, the hsa-miR-1-3p-PAICS axis may restrict the cell division of NSCLC, thereby halting stage advancement.

Along with miRNAs, there are several additional forms of RNAs, including lncRNAs and pseudogenes. They could affect health and illness, including cancer, by binding competitively to common miRNAs as ceRNA [62]. Using the ENCORI database, we acquired 119 upstream pseudogenes of the hsa-miR-1-3p-PAICS axis. The GEPIA database was utilized to better distinguish between NSCLC samples and normal controls, as well as between main phases. Correlation analysis of expression data showed that hsa-miR-1-3p negatively correlated with FAM91A3P, LRRC37A6P, PKMP1, RPL9P32, and BMS1P8. When the ceRNA mechanism and the findings of the preceding investigation are combined, it is verified that pseudogenes may regulate the hsa-miR-1-3p-PAICS in NSCLC. Finally, the lncACTdb, the miRNet, and the ENCORI databases were employed to determine the hsa-miR-1-3p-PAICS axis's upstream regulatory lncRNAs. 33 lncRNAs have commonly appeared in the three databases, which shows many of these lncRNAs functioned as oncogenes in different human cancers. For example, lncRNA UCA1 promotes proliferation, migration, and immune escape and suppresses apoptosis in gastric cancer by binding anti-tumor miRNAs [63]; lncRNA CYTOR promotes the resistance of tamoxifen in breast cancer cells via binding miR-125a-5p [64]; lncRNA RMRP promotes proliferation, migration, and invasion of bladder cancer via miR-206 [65]. The reports above further indicated that these lncRNAs have similarities with those 119 possible pseudogenes, may also participate in hsa-miR-1-3p-PAICS network regulation, thus involving in the development of NSCLC.

Although we constructed the hsa-miR-1-3p-PAICS axis to better understand the occurrence of NSCLC, there are some limitations in our study. Above all, this study lacks experimental verification. Further in vivo and in vitro experiments will be conducted soon to confirm the expression and function of key genes. Additionally, we should further investigate the binding affinity of the biomarkers in our study through experiments.

## 5. Conclusion

In conclusion, integrated bioinformatics investigations indicate that the hsa-miR-1-3p-PAICS axis may contribute to the evolution of NSCLC via mitosis regulation. Additionally, we discovered putative upstream pseudogenes and long non-coding RNAs of the hsa-miR-1-3p-PAICS axis. In the future, the structure of this pseudogene within the lncRNA-hsa-miR-1-3p-PAICS axis may function as a marker and target for treatment.

## Figures and Tables

**Table 1:** Survival analysis of all hub genes showed 8 genes with a prognostic value.

Gene	HR	95% CI	Logrank P
EZH2	1.31	1.15–1.48	3.80E-05
CCNB1	1.62	1.37–1.91	8.70E-09
MMP9	1.14	1–1.29	0.046
SOX2	1.33	1.13–1.57	7.00E-04
FCGR3B	1.2	1.06–1.36	0.0046
IL6	1.32	1.16–1.49	2.00E-05
COL1A1	1.33	1.17–1.51	1.20E-05
PAICS	1.3	1.14–1.47	6.10E-05
CDK1	1.4	1.9–1.21	2.60E-04

**Table 2:** Prediction of miRNAs binding to CCNB1, CDK1, or PAICS.

Gene symbol	miRNA name	Predicting program	Number
CCNB1	Hsa-miR-548b-5p	miRWalK, miRDB, TargetScan	3
CCNB1	Hsa-miR-892b	miRWalK, miRDB, TargetScan	3
CCNB1	Hsa-miR-3130-5p	miRWalK, miRDB, TargetScan	3
CCNB1	Hsa-miR-548w	miRWalK, miRDB, TargetScan	3
CCNB1	Hsa-miR-4482-5p	miRWalK, miRDB, TargetScan	3
CCNB1	Hsa-miR-548aq-5p	miRWalK, miRDB, TargetScan	3
CDK1	Hsa-miR-208a-5p	miRWalK, miRDB, TargetScan	3
CDK1	Hsa-miR-186-3p	miRWalK, miRDB, TargetScan	3
CDK1	Hsa-miR-378a-5p	miRWalK, miRDB, TargetScan	3
CDK1	Hsa-miR-208b-5p	miRWalK, miRDB, TargetScan	3
CDK1	Hsa-miR-1267	miRWalK, miRDB, TargetScan	3
CDK1	Hsa-miR-5009-3p	miRWalK, miRDB, TargetScan	3
CDK1	Hsa-miR-6083	miRWalK, miRDB, TargetScan	3
CDK1	Hsa-miR-6762-3p	miRWalK, miRDB, TargetScan	3
CDK1	Hsa-miR-4698	miRWalK, miRDB, TargetScan	3
CDK1	Hsa-miR-6501-3p	miRWalK, miRDB, TargetScan	3
CDK1	Hsa-miR-188-3p	miRWalK, miRDB	2
PAICS	Hsa-miR-128-3p	PITA, miRmap, microT, miRanda	4
PAICS	Hsa-miR-339-5p	PITA, RNA22, miRmap, miRanda	4
PAICS	Hsa-miR-142-5p	PITA, miRDB, miRmap, microT	4
PAICS	Hsa-miR-146a-5p	PITA, miRDB, miRmap, miRanda	4
PAICS	Hsa-miR-516b-5p	PITA, miRDB, miRmap, microT	4
PAICS	Hsa-miR-340-5p	PITA, miRmap, microT	3
PAICS	Hsa-miR-10b-5p	PITA, miRmap, miRanda	3
PAICS	Hsa-miR-1-3p	PITA, miRanda	2

**Table 3:** The co-expressed genes of PAICS commonly appeared in UALCAN database and GEPIA database.

Common co-expressed genes of PAICS	R^a^	R^b^
PPAT	0.87	0.85
SRP72	0.8	0.75
POLR2B	0.72	0.71
LYAR	0.71	0.67
NCAPG	0.69	0.66
WDR43	0.67	0.61
CCNA2	0.66	0.63
CENPE	0.66	0.64
NAA15	0.66	0.62
ABCE1	0.66	0.61
CHEK1	0.66	0.63
PRR11	0.65	0.59
CCNB1	0.65	0.63
SKA1	0.64	0.61
CDCA5	0.64	0.63
BUB1	0.64	0.6
CDC25A	0.64	0.62
PSMD12	0.64	0.59
MCM10	0.64	0.61
KIF4A	0.64	0.61
CKAP2L	0.63	0.59
TPX2	0.63	0.6
DEPDC1	0.63	0.6
MELK	0.63	0.59
FARSB	0.63	0.59
KIAA1524	0.63	0.59
R3HDM1	0.63	0.61
BUB1B	0.63	0.62
CENPO	0.63	0.59
PA2G4	0.63	0.56
FEN1	0.63	0.62
FANCI	0.62	0.58
PLK1	0.62	0.6
SGOL1	0.62	0.6
WHSC1	0.62	0.6
TTK	0.62	0.59
KIF14	0.62	0.6
TSR1	0.62	0.58
KPNA2	0.62	0.59
ERCC6L	0.62	0.6
FOXM1	0.62	0.62
NCAPH	0.62	0.6
CCT8	0.62	0.57
MCM4	0.62	0.59
KIF18A	0.62	0.57
DKC1	0.61	0.61
DBF4	0.61	0.59
KIF23	0.61	0.58
PRPF40A	0.61	0.54
SMC2	0.61	0.55
DTL	0.61	0.59
INCENP	0.61	0.58
KIF11	0.61	0.57
SHCBP1	0.61	0.54
ARHGAP11A	0.61	0.59
SSRP1	0.61	0.6
POLR1B	0.6	0.5
RAD51	0.6	0.56
FIP1L1	0.6	0.6
MAD2L1	0.6	0.57
GSG2	0.6	0.58
URB2	0.6	0.59
CSE1L	0.6	0.58
NOL10	0.6	0.57
HNRNPR	0.6	0.56
MCM6	0.6	0.57
HNRNPD	0.6	0.55
ANAPC1	0.6	0.51
RNASEH1	0.6	0.54
NUP153	0.6	0.55
CCRN4L	0.6	0.56
DLGAP5	0.6	0.57
NUSAP1	0.6	0.57
MTIF2	0.6	0.54
UHRF1	0.6	0.58
PRC1	0.6	0.59
RRM2	0.6	0.56
UBE2K	0.6	0.52
NOP14	0.6	0.57
BUB3	0.6	0.54
CLSPN	0.6	0.56
ASPM	0.59	0.6
GMPS	0.59	0.55
CCDC86	0.59	0.6
CCT7	0.59	0.57
LMNB2	0.59	0.55
PLK4	0.59	0.6
MASTL	0.59	0.56
DDX18	0.59	0.48
CDC6	0.59	0.56
DIAPH3	0.59	0.52
NEK2	0.59	0.57
EXO1	0.59	0.57
WDR75	0.59	0.55
RACGAP1	0.59	0.54
SPC25	0.59	0.54
SLBP	0.58	0.53
MKI67	0.58	0.57
KPNB1	0.58	0.57
CCT4	0.58	0.5
KIF20A	0.58	0.57
CEP135	0.58	0.54
AURKA	0.58	0.56
TIPIN	0.58	0.55
FAM136A	0.58	0.57
H2AFZ	0.58	0.55
SET	0.58	0.5
EIF2S1	0.58	0.49
SDAD1	0.58	0.54
RAD51AP1	0.58	0.54
CPSF3	0.58	0.48
GRPEL1	0.58	0.54
SUV39H2	0.58	0.55
GART	0.58	0.55
LIN54	0.58	0.53
NOLC1	0.58	0.54
NCL	0.58	0.55
SKA3	0.58	0.55
CDCA3	0.58	0.58
LMNB1	0.58	0.55
KIF2C	0.58	0.56
BIRC5	0.58	0.54
NCAPD2	0.58	0.55
CKAP2	0.58	0.54
NLN	0.57	0.51
GRSF1	0.57	0.52
ANLN	0.57	0.54
STIP1	0.57	0.56
HMMR	0.57	0.52
CKAP5	0.57	0.54
UCHL5	0.57	0.52
POLR2D	0.57	0.49
SSB	0.57	0.53
IMMT	0.57	0.54
PATL1	0.57	0.54
CENPH	0.57	0.55
PNO1	0.57	0.5
PNPT1	0.57	0.53
USP14	0.57	0.5
OLA1	0.57	0.51
RRM1	0.57	0.54
SGOL2	0.57	0.52
FAM83D	0.57	0.56
CENPI	0.57	0.54
HJURP	0.57	0.54
CPSF6	0.57	0.33
CENPN	0.57	0.52
SASS6	0.57	0.54
WDR12	0.57	0.54
HAUS6	0.57	0.52
HEATR1	0.57	0.51
IARS	0.57	0.53
MTBP	0.57	0.54
CEP55	0.57	0.54
POP1	0.56	0.55
PGAM5	0.56	0.53
BRCA1	0.56	0.53
EIF2S2	0.56	0.51
HSPD1	0.56	0.55
PPM1G	0.56	0.53
TOPBP1	0.56	0.54
MSH6	0.56	0.51
ESPL1	0.56	0.57
C18orf54	0.56	0.5
UBA6	0.56	0.53
DENR	0.56	0.52
EXOSC2	0.56	0.51
KIF20B	0.56	0.52
ZWILCH	0.56	0.51
MRTO4	0.56	0.53
ELAVL1	0.56	0.51
CUL2	0.56	0.48
MRPL3	0.56	0.52
PWP1	0.56	0.48
DARS	0.56	0.52
TRA2B	0.56	0.46
UNG	0.56	0.53
CDC25C	0.56	0.54
TMPO	0.56	0.51
NUP37	0.56	0.49
RFWD3	0.55	0.5
RRP1B	0.55	0.52
EIF4E	0.55	0.5
GTSE1	0.55	0.53
MTHFD1L	0.55	0.49

^a^Correlation coefficient determined by UALCAN database. ^b^Correlation coefficient determined by GEPIA database.

## Data Availability

The data used to support the findings of this study are included within the supplementary information files.

## Supplementary Materials

Supplementary Materials Table S1. The significant DEGs between normal samples and NSCLC. Table S2. The co-expressed genes of SPOCK2 from UALCAN and GEPIA databases. Table S3. The potential upstream lncRNAs of hsa-miR-1-3p predicted by ENCOLI, miRNet and LncACTdb databases.
