# Supplementary material for: Dysregulation of Pseudogenes/lncRNA-Hsa-miR-1-3p-PAICS Pathway Promotes the Development of NSCLC
Source: J Oncol. 2022 Aug 30;2022:4714931. doi: 10.1155/2022/4714931 (PMC9448537; doi:10.1155/2022/4714931)
Supplement: Supplementary Materials — Table S1. The significant DEGs between normal samples and NSCLC. Table S2. The co-expressed genes of SPOCK2 from UALCAN and GEPIA databases. Table S3. The potential upstream lncRNAs of hsa-miR-1-3p predicted by ENCOLI, miRNet and LncACTdb databases. [file 4714931.f1.zip › 4714931.f1/Supplementary Table S1.docx]

**Supplementary Table S1.** **The significant DEGs between normal samples and NSCLC.**

| Upregulated DEGs in NSCLC samples | Downregulated DEGs in NSCLC samples |
| --- | --- |
| KRT6A | ADH1B |
| MMP12 | CLDN18 |
| AKR1B10 | AGER |
| MMP1 | SFTPC |
| S100A2 | AQP4 |
| GJB2 | CYP4B1 |
| GREM1 | TMEM100 |
| COL11A1 | FABP4 |
| TOP2A | GKN2 |
| PSAT1 | FAM107A |
| SPP1 | MT1M |
| KRT15 | CPB2 |
| GPX2 | CLIC5 |
| SLC2A1 | GPM6A |
| SOX2 | PEBP4 |
| ANLN | TNNC1 |
| CDC20 | MCEMP1 |
| TMPRSS4 | FCN3 |
| TPX2 | WIF1 |
| RRM2 | ABCA8 |
| BIRC5 | SFTPD |
| ASPM | CA4 |
| IGF2BP3 | ADAMTS8 |
| CCNB1 | INMT |
| WDR72 | MAMDC2 |
| DSP | SCN7A |
| CCNB2 | VEPH1 |
| GPR87 | LRRK2 |
| DLGAP5 | CHRDL1 |
| KIAA0101 | COL4A3 |
| UBE2C | SUSD2 |
| COL10A1 | TCF21 |
| TFAP2A | PGC |
| CTHRC1 | SCGB1A1 |
| CXCL14 | ADRB1 |
| BUB1B | FOSB |
| MAD2L1 | FAM150B |
| CDCA7 | CXCL2 |
| CENPF | PDK4 |
| UHRF1 | C2orf40 |
| PBK | FMO2 |
| GINS1 | NOSTRIN |
| CEP55 | SCGB3A2 |
| TTK | SELE |
| PRC1 | C7 |
| MELK | SDPR |
| SULF1 | PTPRB |
| NEK2 | BTNL9 |
| FERMT1 | CD36 |
| KIF2C | PLA2G1B |
| UBE2T | SFTA1P |
| BUB1 | ACKR1 |
| SIX1 | CACNA2D2 |
| FOXM1 | MARCO |
| CDH3 | FHL1 |
| NUSAP1 | LPL |
| HS6ST2 | HPGD |
| ECT2 | LIMCH1 |
| WDR66 | OLR1 |
| CDKN3 | GPIHBP1 |
| MMP10 | ABI3BP |
| KIF11 | ZNF385B |
| KIF4A | ZBTB16 |
| TK1 | ADIRF |
| MCM2 | HHIP |
| MMP11 | HSD17B6 |
| AURKA | AFF3 |
| CXCL13 | FAM189A2 |
| ZWINT | OGN |
| NDC80 | SCARA5 |
| CEACAM5 | IL6 |
| KIF20A | GLDN |
| THBS2 | HBB |
| WISP1 | ACADL |
| NUF2 | PPBP |
| NMU | PLAC9 |
| HMGB3 | EMCN |
| CENPU | STX11 |
| FNDC1 | MFAP4 |
| CDK1 | EDNRB |
| DEPDC1 | CFD |
| SRD5A1 | ALOX5 |
| FBXO32 | TEK |
| ADAMDEC1 | SLC1A1 |
| RMI2 | FLRT3 |
| PPAP2C | CX3CR1 |
| HMMR | GPX3 |
| IGSF9 | IL33 |
| KIF14 | CD52 |
| EZH2 | COL6A6 |
| ZNF750 | CLIC3 |
| COL5A2 | FRY |
| PTTG1 | AOC3 |
| TYMS | LYVE1 |
| MCM4 | RGCC |
| SLC7A11 | PIP5K1B |
| LGR4 | PLEKHH2 |
| CCDC34 | SLC39A8 |
| NQO1 | DLC1 |
| PLOD2 | GPR133 |
| CASC5 | ITGA8 |
| CRABP2 | CHI3L2 |
| CCNA2 | SFTPB |
| KIF26B | ANKRD29 |
| ANKRD22 | CAB39L |
| GPT2 | NDNF |
| NCAPG | LAMP3 |
| TRIM59 | GIMAP8 |
| C15orf48 | ARHGAP6 |
| FAP | ROBO4 |
| EGLN3 | LDB2 |
| LRRC15 | CXCL3 |
| CST1 | MS4A7 |
| HMGB3P1 | CDO1 |
| PPAT | ANXA3 |
| IGFBP3 | CD69 |
| RP11-44F21.5 | WISP2 |
| ADAMTS12 | VSIG4 |
| C1orf106 | CAV1 |
| KIF15 | PTPN21 |
| COL5A1 | MYZAP |
| MXRA5 | FXYD1 |
| SCG5 | SLC46A2 |
| PTGFRN | C1orf162 |
| E2F8 | HIGD1B |
| RASEF | DNASE1L3 |
| SIX4 | AQP1 |
| FIGNL1 | PGM5 |
| STIL | ADTRP |
| CCNE2 | RGS13 |
| BAIAP2L1 | FOXF1 |
| SRPX2 | PHACTR1 |
| MMP9 | PPP1R14A |
| CLDN1 | SCEL |
| PAICS | ADRB2 |
| FEN1 | GATA6 |
| BRIP1 | FCN1 |
| LEPREL4 | SOX17 |
| COL3A1 | S100A12 |
| SORD | ARRB1 |
| NME1 | MSR1 |
| TNC | CSRNP1 |
| LAD1 | NR4A2 |
| TENM4 | SOX7 |
| GOLM1 | TPPP3 |
| FLJ13744 | FRMD3 |
| CEMIP | FGR |
| TMPRSS11E | DPEP2 |
| GPX8 | KAL1 |
| PLAU | FAM162B |
| TPBG | GADD45B |
| SHCBP1 | ABCA3 |
| SPINK1 | KCNT2 |
| PAFAH1B3 | SPOCK2 |
| PDK1 | NTN4 |
| TSPAN6 | KANK3 |
| MMP13 | ECSCR |
| PYCR1 | CYYR1 |
| BIK | CXCR2 |
| CENPK | DUOX1 |
| PCAT6 | MS4A15 |
| SDC1 | FHL5 |
| HN1 | CDH5 |
| DEPDC1B | GNG11 |
| MFAP2 | JAM2 |
| KISS1R | GDF10 |
| MDK | AGTR1 |
| FAXC | CCM2L |
| HIST1H2BD | RAMP3 |
| LCN2 | KLF2 |
| CP | PCOLCE2 |
| PPAPDC1A | CCBE1 |
| F2RL1 | UPK3B |
| COL1A1 | ATOH8 |
| EFNA4 | FILIP1 |
| KRT80 | SLC6A14 |
| EPHX3 | HYAL1 |
| GRTP1 | VIPR1 |
| MND1 | PALMD |
| GMNN | ADAMTSL4 |
| KDELR3 | MNDA |
| SERINC2 | TMEM139 |
| TRIM2 | SMAD9 |
| HOXB7 | SPIDR |
| UGT8 | ADARB1 |
| SFN | GIMAP6 |
| TMEM177 | LIFR |
| GGCT | DACH1 |
| GCNT3 | GPRC5A |
| ATP10B | LOC101926959 |
| DNAH14 | TSPAN12 |
| PDIA4 | GIMAP7 |
| ALDH18A1 | PROK2 |
| OCIAD2 | FBLN5 |
| SFXN1 | RAI2 |
| CD24 | LRRN3 |
| TDO2 | MYRF |
| CDKN2A | CAMK2N1 |
| NGEF | GRK5 |
| PLEK2 | TIE1 |
| FUT2 | MYCT1 |
| FUT3 | EP300-AS1 |
| PAK6 | GLIPR2 |
| KCNK1 | PDZD2 |
| LRIG3 | GRAMD2 |
| XPR1 | ZBED2 |
| PCP4 | SMAD6 |
| ERO1L | F8 |
| RUNX2 | PCDH9 |
| MUC20 | PTGDS |
| NHS | COBL |
| MAP7D2 | CPA3 |
| RCC1 | NR4A3 |
| RGS17 | RBMS3 |
| TOX3 | RBP4 |
| MSI2 | ANK2 |
| AFAP1-AS1 | CCL2 |
| SUGCT | LINC00312 |
| HN1L | ESAM |
| TLCD1 | NCKAP5 |
| PSPH | PLLP |
| SOX4 | IGSF10 |
| TCN1 | PCAT19 |
| GALNT7 | LINC00968 |
| COMP | ID4 |
| DEPDC7 | BRE-AS1 |
| C2CD4A | RASIP1 |
| GFPT1 | ANGPT1 |
| DIO2 | FOS |
| SRD5A3 | SERTM1 |
| IGFL2 | TPSB2 |
| STYK1 | BMP2 |
| ARHGEF19 | CLDN5 |
| SPAG4 | PPARG |
| ITGA11 | PID1 |
| ORC6 | PAQR5 |
| DNAJC12 | DOCK4 |
| FHL2 | TPSAB1 |
| LEMD1 | LOC643733 |
| LAPTM4B | CLEC14A |
| ADAM28 | FAM13C |
| TXNDC17 | PTPRM |
| PPAPDC1B | ASPA |
| CDH1 | RTKN2 |
| ST14 | NPNT |
| LGSN | SELP |
| THY1 | EDN1 |
| LSR | ALOX5AP |
| GALNT3 | PRKCE |
| GMDS | ZFP36 |
| SLC39A11 | SOCS2 |
| FRK | NEBL |
| CEACAM1 | LIMS2 |
| HOOK1 | TMEM178A |
| MMP7 | PECAM1 |
| LAMP5 | ICAM2 |
| CFB | TM6SF1 |
| SFRP2 | C1QTNF7 |
| TUBB2B | C14orf132 |
| BZW2 | COX7A1 |
| CILP2 | TNS1 |
|  | C11orf96 |
|  | SCN4B |
|  | SRPX |
|  | TGFBR3 |
|  | MMRN1 |
|  | MMP19 |
|  | NR4A1 |
|  | EMP2 |
|  | SLCO2A1 |
|  | S100A4 |
|  | HBEGF |
|  | GUCY1A2 |
|  | ERG |
|  | CSF3 |
|  | LINC01140 |
|  | FGD5 |
|  | MSLN |
|  | SLIT2 |
|  | MAOB |
|  | GMFG |
|  | MFNG |
|  | DENND3 |
|  | ITIH5 |
|  | CPED1 |
|  | ARHGEF26 |
|  | SSTR1 |
|  | STARD13 |
|  | HSPA12B |
|  | CST6 |
|  | MME |
|  | AREG |
|  | MS4A2 |
|  | MYH11 |
|  | DAPK2 |
|  | CA2 |
|  | ZEB2 |
|  | ARHGAP29 |
|  | HOXA5 |
|  | GPR146 |
|  | SLC6A4 |
|  | VWF |
|  | C5AR1 |
|  | TSPAN7 |
|  | GIMAP1 |
|  | FLJ35700 |
|  | EPAS1 |
|  | SEMA3G |
|  | LIN7A |
|  | NEDD9 |
|  | PLA1A |
|  | APOLD1 |
|  | NEXN |
|  | ADAMTSL3 |
|  | LRRC32 |
|  | TAL1 |
|  | CD97 |
|  | MTURN |
|  | PDE5A |
|  | GATA2 |
|  | DUSP1 |
|  | ITM2A |
|  | STXBP6 |
|  | RAMP2 |
|  | P2RY14 |
|  | TGFBR2 |
|  | KL |
|  | ARHGAP31 |
|  | RECK |
|  | DPYSL2 |
|  | ACVRL1 |
|  | FAM167A |
|  | NFKBIZ |
|  | OR7E47P |
|  | KIAA1462 |
|  | OSCAR |
|  | S1PR1 |
|  | CXCL12 |
|  | CARD16 |
|  | CD93 |
|  | DKK2 |
|  | RASSF2 |
|  | ATF3 |
|  | MMRN2 |
|  | PPP1R15A |
|  | TLR8 |
|  | ACKR4 |
|  | SLPI |
|  | KLF9 |
|  | HSPB8 |
|  | SECISBP2L |
|  | CALCRL |
|  | CLEC12A |
|  | CYBRD1 |
|  | SYNC |
|  | COX4I2 |
|  | EGR3 |
|  | GPM6B |
|  | CNRIP1 |
|  | NLRC4 |
|  | FPR1 |
|  | TBX5-AS1 |
|  | ADAMTS1 |
|  | RP11-389C8.2 |
|  | LMO2 |
|  | TMEM204 |
|  | FLI1 |
|  | ABCG2 |
|  | PTGS2 |
|  | THBD |
|  | PAPSS2 |
|  | PDLIM2 |
|  | TMOD1 |
|  | ECM2 |
|  | IL1B |
|  | IL1RL1 |
|  | PDE2A |
|  | GPR126 |
|  | KLF6 |
|  | MYADM |
|  | CLC |
|  | FIBIN |
|  | FAM46B |
|  | GIMAP4 |
|  | CDKN2B |
|  | SOCS3 |
|  | SH2D3C |
|  | FAT3 |
|  | TPPP |
|  | SIRPB1 |
|  | TBX2 |
|  | SGCG |
|  | CRTAM |
|  | GYPC |
|  | CRTAC1 |
|  | PLAC8 |
|  | CAV2 |
|  | KLRF1 |
|  | KLF4 |
|  | SCAI |
|  | REEP1 |
|  | CLEC1A |
|  | GPR65 |
|  | BCHE |
|  | RGS18 |
|  | PILRA |
|  | RCSD1 |
|  | G0S2 |
|  | NKG7 |
|  | KIAA1324L |
|  | RASL12 |
|  | AQP9 |
|  | SHANK3 |
|  | PRELP |
|  | FCGR3B |
|  | KANK2 |
|  | VGLL3 |
|  | IRAK3 |
|  | EMR1 |
|  | BCL2A1 |
|  | RFX2 |
|  | TCEAL2 |
|  | SLC14A1 |
|  | ACE |
|  | MAGI2-AS3 |
|  | RBMS2 |
|  | SPARCL1 |
|  | ST6GALNAC5 |
|  | RGS2 |
|  | ZNF331 |
|  | VNN2 |
|  | GRIA1 |
|  | CBX7 |
|  | P2RY13 |
|  | ITGA1 |
|  | ABCB1 |
|  | TOX2 |
|  | CH25H |
|  | TIMP3 |
|  | LHFP |
|  | TBX5 |
|  | LOC731424 |
|  | TLR4 |
|  | CLDN11 |
|  | METTL7A |
|  | C1orf115 |
|  | PTX3 |
|  | KCNAB1 |
|  | MAP2 |
|  | ITGAL |
|  | TMEM88 |
|  | C10orf10 |
|  | MAP3K8 |
|  | MSRB3 |
|  | TMEM47 |
|  | GZMH |
|  | PKIG |
|  | IL18RAP |
|  | CCL23 |
|  | WASF3 |
|  | DOCK11 |
|  | DES |
|  | NEGR1 |
|  | CMTM2 |
|  | LTBP4 |
|  | RP11-247L20.4 |
|  | SYNPO2 |
|  | RP1-78O14.1 |
|  | GNLY |
|  | PROS1 |
|  | BTG2 |
|  | AKAP12 |
|  | CDKN1C |
|  | RAB11FIP1 |
|  | EFEMP1 |
|  | ROBO2 |
|  | SASH1 |
|  | CCDC68 |
|  | COL13A1 |
|  | DQ592442 |
|  | mir-223 |
|  | CCDC102B |
|  | AX748273 |
|  | NOTCH4 |
|  | LGI3 |
|  | FERMT2 |
|  | SCNN1G |
|  | FXYD6 |
|  | MAL |
|  | DST |
|  | LOC285043 |
|  | RRAS |
|  | RUNX1T1 |
|  | MAFF |
|  | RNF144B |
|  | AIF1L |
|  | ARC |
|  | ELTD1 |
|  | C20orf85 |
|  | PTPLA |
|  | ANGPTL1 |
|  | APOL3 |
|  | HECW2 |
|  | SVEP1 |
|  | PEAK1 |
|  | FMO3 |
|  | ARHGEF10 |
|  | RNF125 |
|  | SYNM |
|  | TCEAL7 |
|  | CXCL5 |
|  | FZD4 |
|  | SPTBN1 |
|  | C9orf24 |
|  | SLC16A6 |
|  | ABLIM3 |
|  | JUNB |
|  | LATS2 |
|  | OSR1 |
|  | ABCA6 |
|  | KIAA0040 |
|  | SAMD4A |
|  | EGR1 |
|  | TTN |
|  | AFAP1L1 |
|  | SLC19A3 |
|  | PREX2 |
|  | BCL6B |
|  | SEMA5A |
|  | LEPREL1 |
|  | GJA5 |
|  | PTGER4 |
|  | ARAP3 |
|  | SLC2A3 |
|  | SNTN |
|  | RXFP1 |
|  | FAM134B |
|  | OTUD1 |
|  | SH3GL3 |
|  | OLFML1 |
|  | MYL9 |
|  | CCL4 |
|  | SERPING1 |
|  | PMP22 |
|  | CADM1 |
|  | PDE8B |
|  | BMP5 |
|  | WFS1 |
|  | PRF1 |
|  | TRHDE |
|  | PODXL |
|  | C10orf54 |
|  | ST6GALNAC3 |
|  | GNG2 |
|  | S100A3 |
|  | DPT |
|  | TACC1 |
|  | CSF2RB |
|  | SEMA6A |
|  | DYNLRB2 |
|  | SOSTDC1 |
|  | KLRB1 |
|  | RASSF8 |
|  | LSAMP |
|  | CFP |
|  | TRPC6 |
|  | MYH10 |
|  | AOX1 |
|  | CTNNAL1 |
|  | PEAR1 |
|  | NHSL1 |
|  | SIK1 |
|  | GIPC2 |
|  | AGTPBP1 |
|  | LGALSL |
|  | CDH19 |
|  | CD274 |
|  | KCNJ8 |
|  | PKNOX2 |
|  | ID1 |
|  | RP11-401P9.4 |
|  | RAB8B |
|  | CORO2B |
|  | LDLR |
|  | RORA |
|  | FAM101B |
|  | RSPO3 |
|  | RAPGEF4 |
|  | SLIT3 |
|  | PTGIS |
|  | ELMO1 |
|  | GPC3 |
|  | FBLN1 |
|  | IL18R1 |
|  | KLRD1 |
|  | SNRK |
|  | NES |
|  | PPAP2B |
|  | LMCD1 |
|  | FPR2 |
|  | CYR61 |
|  | SPRYD7 |
|  | TNFRSF10D |
|  | EPB41L3 |
|  | ATP1A2 |
|  | DNAJB4 |
|  | RHOB |
|  | PCDH17 |
|  | IL7R |
|  | CLEC4E |
|  | DENND2A |
|  | ZC3H12C |
|  | PLCE1 |
|  | CFL2 |
|  | KRT4 |
|  | FKBP1B |
|  | SBSPON |
|  | MGAM |
|  | RARRES2 |
|  | PREX1 |
|  | CXorf36 |
|  | DIXDC1 |
|  | FENDRR |
|  | MCOLN3 |
|  | RNF182 |
|  | ID3 |
|  | LOC101928370 |
|  | HEG1 |
|  | TJP2 |
|  | NDRG2 |
|  | CASKIN2 |
|  | CBFA2T3 |
|  | EBF1 |
|  | HSPB6 |
|  | GALNT18 |
|  | CA3 |
|  | SGCE |
|  | EYA4 |
|  | FRAS1 |
|  | ETS1 |
|  | AHNAK |
|  | TNFAIP3 |
|  | S100A8 |
|  | FEZ1 |
|  | LRCH2 |
|  | KLB |
|  | FGF7 |
|  | LOC285812 |
|  | KDR |
|  | CNTN6 |
|  | PRICKLE2 |
|  | RCAN1 |
|  | JAM3 |
|  | PTRF |
|  | PLCB4 |
|  | NTNG1 |
|  | SLC5A9 |
|  | PPARGC1A |
