# Supplementary material for: Dysregulation of Pseudogenes/lncRNA-Hsa-miR-1-3p-PAICS Pathway Promotes the Development of NSCLC
Source: J Oncol. 2022 Aug 30;2022:4714931. doi: 10.1155/2022/4714931 (PMC9448537; doi:10.1155/2022/4714931)
Supplement: Supplementary Materials — Table S1. The significant DEGs between normal samples and NSCLC. Table S2. The co-expressed genes of SPOCK2 from UALCAN and GEPIA databases. Table S3. The potential upstream lncRNAs of hsa-miR-1-3p predicted by ENCOLI, miRNet and LncACTdb databases. [file 4714931.f1.zip › 4714931.f1/Supplementary Table S2.docx]

**Supplementary Table S2. The co-expressed genes of SPOCK2 from UALCAN and GEPIA databases.**

| Co-expressed genes determined by GEPIA | Co-expressed genes determined by UALCAN | Co-expressed genes determined by both GEPIA and UALCAN |
| --- | --- | --- |
| PPAT | PPAT | PPAT |
| SRP72 | SRP72 | SRP72 |
| POLR2B | POLR2B | POLR2B |
| LYAR | LYAR | LYAR |
| NCAPG | NCAPG | NCAPG |
| WDR43 | CENPE | WDR43 |
| CCNA2 | CCNA2 | CCNA2 |
| CENPE | CHEK1 | CENPE |
| NAA15 | CDCA5 | NAA15 |
| ABCE1 | CCNB1 | ABCE1 |
| CHEK1 | FOXM1 | CHEK1 |
| PRR11 | NAA15 | PRR11 |
| CCNB1 | CDC25A | CCNB1 |
| SKA1 | FEN1 | SKA1 |
| CDCA5 | BUB1B | CDCA5 |
| BUB1 | KIF4A | BUB1 |
| CDC25A | WDR43 | CDC25A |
| PSMD12 | ABCE1 | PSMD12 |
| MCM10 | DKC1 | MCM10 |
| KIF4A | SKA1 | KIF4A |
| CKAP2L | MCM10 | CKAP2L |
| TPX2 | R3HDM1 | TPX2 |
| DEPDC1 | SGOL1 | DEPDC1 |
| MELK | KIF14 | MELK |
| FARSB | BUB1 | FARSB |
| KIAA1524 | ERCC6L | KIAA1524 |
| R3HDM1 | SSRP1 | R3HDM1 |
| BUB1B | CCDC86 | BUB1B |
| CENPO | NCAPH | CENPO |
| PA2G4 | ASPM | PA2G4 |
| FEN1 | FIP1L1 | FEN1 |
| FANCI | WHSC1 | FANCI |
| PLK1 | DEPDC1 | PLK1 |
| SGOL1 | PLK4 | SGOL1 |
| WHSC1 | TPX2 | WHSC1 |
| NOA1 | PLK1 | TTK |
| TTK | KIAA1524 | KIF14 |
| KIF14 | KPNA2 | TSR1 |
| TSR1 | DTL | KPNA2 |
| KPNA2 | TTK | ERCC6L |
| ERCC6L | MELK | FOXM1 |
| FOXM1 | URB2 | NCAPH |
| NCAPH | PRC1 | CCT8 |
| CCT8 | MCM4 | MCM4 |
| CTD-2510F5.4 | DBF4 | KIF18A |
| MCM4 | ARHGAP11A | DKC1 |
| KIF18A | PSMD12 | DBF4 |
| DKC1 | PRR11 | KIF23 |
| DBF4 | FARSB | PRPF40A |
| KIF23 | CENPO | SMC2 |
| PRPF40A | CKAP2L | DTL |
| SMC2 | C4orf14 | INCENP |
| PAICSP4 | CSE1L | KIF11 |
| DTL | TSR1 | SHCBP1 |
| INCENP | CDCA3 | ARHGAP11A |
| KIF11 | KIF23 | SSRP1 |
| NIFK | INCENP | POLR1B |
| SHCBP1 | SPAG5 | RAD51 |
| ARHGAP11A | FANCI | FIP1L1 |
| SSRP1 | UHRF1 | MAD2L1 |
| POLR1B | GSG2 | GSG2 |
| RAD51 | ESPL1 | URB2 |
| FIP1L1 | FAM136A | CSE1L |
| MAD2L1 | CCT7 | NOL10 |
| GSG2 | MKI67 | HNRNPR |
| URB2 | KIF20A | MCM6 |
| RP11-424C20.2 | NUSAP1 | HNRNPD |
| CSE1L | KIF11 | ANAPC1 |
| NOL10 | CCT8 | RNASEH1 |
| HNRNPR | XPO5 | NUP153 |
| MCM6 | MAD2L1 | CCRN4L |
| HNRNPD | EXO1 | DLGAP5 |
| ANAPC1 | NEK2 | NUSAP1 |
| RNASEH1 | C15orf42 | MTIF2 |
| NUP153 | NOL10 | UHRF1 |
| CCRN4L | C11orf82 | PRC1 |
| DLGAP5 | TOP2A | RRM2 |
| NUSAP1 | KIF18A | UBE2K |
| MTIF2 | MCM6 | NOP14 |
| UHRF1 | KPNB1 | BUB3 |
| PRC1 | NOP14 | CLSPN |
| RRM2 | DLGAP5 | ASPM |
| UBE2K | RRM2 | GMPS |
| NOP14 | HNRNPR | CCDC86 |
| BUB3 | CLSPN | CCT7 |
| CLSPN | LRPPRC | LMNB2 |
| ORC1 | CDC20 | PLK4 |
| ASPM | CASC5 | MASTL |
| GMPS | CCRN4L | DDX18 |
| CCDC86 | AURKB | CDC6 |
| CCT7 | ORC1L | DIAPH3 |
| LMNB2 | RAD51 | NEK2 |
| PLK4 | C1orf135 | EXO1 |
| MASTL | MASTL | WDR75 |
| DDX18 | FAM83D | RACGAP1 |
| CDC6 | CENPF | SPC25 |
| DDIAS | CDC6 | SLBP |
| DIAPH3 | AURKA | MKI67 |
| NEK2 | STIP1 | KPNB1 |
| EXO1 | KIF2C | CCT4 |
| WDR75 | PA2G4 | KIF20A |
| RACGAP1 | LMNB1 | CEP135 |
| SPC25 | NCL | AURKA |
| SLBP | SKA3 | TIPIN |
| MKI67 | SUV39H2 | FAM136A |
| KPNB1 | HNRNPD | H2AFZ |
| CCT4 | NUP153 | SET |
| KIF20A | LMNB2 | EIF2S1 |
| CEP135 | WDR75 | SDAD1 |
| AURKA | GART | RAD51AP1 |
| TIPIN | DDX10 | CPSF3 |
| AUNIP | FAM72B | GRPEL1 |
| FAM136A | C21orf45 | SUV39H2 |
| H2AFZ | GAR1 | GART |
| SET | KIF18B | LIN54 |
| EIF2S1 | SMC2 | NOLC1 |
| SDAD1 | NCAPD2 | NCL |
| RAD51AP1 | TIPIN | SKA3 |
| CPSF3 | POP1 | CDCA3 |
| RP4-785G19.2 | TCOF1 | LMNB1 |
| MIS18A | CENPH | KIF2C |
| GRPEL1 | HSPD1 | BIRC5 |
| SUV39H2 | H2AFZ | NCAPD2 |
| GART | CHAF1A | CKAP2 |
| LIN54 | NOL11 | NLN |
| NOLC1 | GMPS | GRSF1 |
| NCL | CDCA2 | ANLN |
| TMA16 | ARHGAP11B | STIP1 |
| SKA3 | CKAP5 | HMMR |
| CDCA3 | CDKN3 | CKAP5 |
| LMNB1 | BIRC5 | UCHL5 |
| KIF2C | RACGAP1 | POLR2D |
| BIRC5 | FAM72A | SSB |
| NCAPD2 | NUP205 | IMMT |
| CKAP2 | C15orf23 | PATL1 |
| NLN | RNASEH1 | CENPH |
| GRSF1 | CDC25C | PNO1 |
| TICRR | CKAP2 | PNPT1 |
| ANLN | SPC25 | USP14 |
| STIP1 | RRM1 | OLA1 |
| KNSTRN | GRPEL1 | RRM1 |
| HMMR | CENPI | SGOL2 |
| CKAP5 | ANLN | FAM83D |
| UCHL5 | PRKDC | CENPI |
| POLR2D | CEP55 | HJURP |
| SSB | MTIF2 | CPSF6 |
| IMMT | CEP135 | CENPN |
| PATL1 | EEF1E1 | SASS6 |
| CENPH | KIF15 | WDR12 |
| PNO1 | HJURP | HAUS6 |
| PNPT1 | PRPF40A | HEATR1 |
| USP14 | BUB3 | IARS |
| OLA1 | TOPBP1 | MTBP |
| RRM1 | TRIP13 | CEP55 |
| SGOL2 | SASS6 | POP1 |
| FAM83D | PATL1 | PGAM5 |
| CENPI | SDAD1 | BRCA1 |
| HJURP | IMMT | EIF2S2 |
| CPSF6 | WDR12 | HSPD1 |
| CENPN | RAD51AP1 | PPM1G |
| SASS6 | MTBP | TOPBP1 |
| WDR12 | SHCBP1 | MSH6 |
| HAUS6 | NOLC1 | ESPL1 |
| HEATR1 | UNG | C18orf54 |
| IARS | CDC45 | UBA6 |
| MTBP | DSCC1 | DENR |
| CEP55 | RBM28 | EXOSC2 |
| ZPR1 | CLPB | KIF20B |
| POP1 | CCNF | ZWILCH |
| PGAM5 | RUVBL1 | MRTO4 |
| BRCA1 | ATAD5 | ELAVL1 |
| EIF2S2 | VRK1 | CUL2 |
| HSPD1 | DHX15 | MRPL3 |
| PPM1G | NCAPD3 | PWP1 |
| TOPBP1 | IARS | DARS |
| MSH6 | PNPT1 | TRA2B |
| ESPL1 | BRCA1 | UNG |
| C18orf54 | MRTO4 | CDC25C |
| UBA6 | SSB | TMPO |
| DENR | RIOK1 | NUP37 |
| EXOSC2 | ENOPH1 | RFWD3 |
| KIF20B | GTSE1 | RRP1B |
| ZWILCH | SLBP | EIF4E |
| MRTO4 | UBA6 | GTSE1 |
| ELAVL1 | FAM72D | MTHFD1L |
| CUL2 | NDC80 |  |
| MRPL3 | BRCA2 |  |
| PWP1 | PPM1G |  |
| DARS | PGAM5 |  |
| TRA2B | PRPF4 |  |
| UNG | RAD54L |  |
| CDC25C | TACC3 |  |
| TMPO | LIN54 |  |
| NUP37 | SNRPD1 |  |
| ORC6 | CCNB2 |  |
| RFWD3 | EFTUD2 |  |
| RRP1B | TEX10 |  |
| EIF4E | UBE2K |  |
| GTSE1 | NUF2 |  |
| MTHFD1L | ORC6L |  |
|  | GRSF1 |  |
|  | MRPL3 |  |
|  | GTPBP4 |  |
|  | CDCA8 |  |
|  | UBE2T |  |
|  | EIF2C2 |  |
|  | EPR1 |  |
|  | KIF20B |  |
|  | ATIC |  |
|  | EXOC1 |  |
|  | KIFC1 |  |
|  | CENPA |  |
|  | C1orf163 |  |
|  | CENPN |  |
|  | UCHL5 |  |
|  | DARS |  |
|  | DIAPH3 |  |
|  | HAUS6 |  |
|  | DENR |  |
|  | LETM1 |  |
|  | RRP1B |  |
|  | RAD54B |  |
|  | NCAPG2 |  |
|  | HMMR |  |
|  | SGOL2 |  |
|  | TMPO |  |
|  | FBXO5 |  |
|  | PUS7 |  |
|  | EIF2S2 |  |
|  | MSH2 |  |
|  | MKI67IP |  |
|  | NOP58 |  |
|  | POLR1A |  |
|  | CCDC99 |  |
|  | ZNF259 |  |
|  | HEATR1 |  |
|  | NLN |  |
|  | TAF5 |  |
|  | ZWINT |  |
|  | TIMELESS |  |
|  | NFXL1 |  |
|  | MTHFD2 |  |
|  | C12orf48 |  |
|  | DCAF13 |  |
|  | XRCC2 |  |
|  | EXOSC9 |  |
|  | FBXO45 |  |
|  | FAM64A |  |
|  | SFRS1 |  |
|  | EXOSC2 |  |
|  | ELAVL1 |  |
|  | WDHD1 |  |
|  | NAA25 |  |
|  | MCM2 |  |
|  | RFC5 |  |
|  | DARS2 |  |
|  | KDM1A |  |
|  | OLA1 |  |
|  | MSH6 |  |
|  | ANAPC1 |  |
|  | SF3B2 |  |
|  | ECT2 |  |
|  | THOC4 |  |
|  | DHX33 |  |
|  | ZWILCH |  |
|  | ATAD2 |  |
|  | TCP1 |  |
|  | PAWR |  |
|  | EIF4E |  |
|  | CCT3 |  |
|  | TOMM70A |  |
|  | FANCD2 |  |
|  | DHX36 |  |
|  | MTHFD1 |  |
|  | POLA2 |  |
|  | CCT4 |  |
|  | OIP5 |  |
|  | TCERG1 |  |
|  | WDR3 |  |
|  | RPP40 |  |
|  | LARP1 |  |
|  | H2AFX |  |
|  | TDG |  |
|  | TMEM48 |  |
|  | RANBP1 |  |
|  | CAD |  |
|  | C18orf54 |  |
|  | POLR1B |  |
|  | MRPL19 |  |
|  | RFWD3 |  |
|  | PNO1 |  |
|  | POC1A |  |
|  | PDSS1 |  |
|  | HSPA4 |  |
|  | PBK |  |
|  | USP14 |  |
|  | PSMD11 |  |
|  | ABCF2 |  |
|  | L2HGDH |  |
|  | SET |  |
|  | CDK2 |  |
|  | MTA2 |  |
|  | RIF1 |  |
|  | DBF4B |  |
|  | RBL1 |  |
|  | EIF5B |  |
|  | C3orf26 |  |
|  | EME1 |  |
|  | BRIP1 |  |
|  | CCT5 |  |
|  | PSME3 |  |
|  | CCDC58 |  |
|  | DNAJC9 |  |
|  | TRAIP |  |
|  | CDT1 |  |
|  | MTHFD1L |  |
|  | NOL7 |  |
|  | POLR2D |  |
|  | WDR76 |  |
|  | LSM12 |  |
|  | YWHAG |  |
|  | FAM54A |  |
|  | RFC3 |  |
|  | PRPF19 |  |
|  | KIAA0406 |  |
|  | RNF26 |  |
|  | PTCD3 |  |
|  | ZC3H8 |  |
|  | SNRPA1 |  |
|  | HELLS |  |
|  | TUBG1 |  |
|  | KHDRBS1 |  |
|  | NUP37 |  |
|  | CLOCK |  |
|  | C2orf44 |  |
|  | C1orf112 |  |
|  | TYMS |  |
|  | DEK |  |
|  | POLQ |  |
|  | NUDCD1 |  |
|  | TEAD4 |  |
|  | CCDC43 |  |
|  | EIF2S1 |  |
|  | DHX37 |  |
|  | UBE2V2 |  |
|  | HNRNPL |  |
|  | PSMD2 |  |
|  | PAK1IP1 |  |
|  | DDB1 |  |
|  | EIF3J |  |
|  | DDX21 |  |
|  | CPSF3 |  |
|  | HMGB2 |  |
|  | GINS4 |  |
|  | PKMYT1 |  |
|  | CHAF1B |  |
|  | DLAT |  |
|  | ANP32B |  |
|  | SFXN1 |  |
|  | UCK2 |  |
|  | DONSON |  |
|  | AASDH |  |
|  | MYBL2 |  |
|  | ANAPC7 |  |
|  | WDR67 |  |
|  | BCCIP |  |
|  | CHORDC1 |  |
|  | C9orf140 |  |
|  | CENPK |  |
|  | CHCHD3 |  |
|  | SERBP1 |  |
|  | UTP18 |  |
|  | HLTF |  |
|  | C16orf59 |  |
|  | NCBP1 |  |
|  | DDX18 |  |
|  | POLR3G |  |
|  | TTL |  |
|  | E2F2 |  |
|  | TTF2 |  |
|  | HNRNPC |  |
|  | LRRC59 |  |
|  | C4orf43 |  |
|  | ESCO2 |  |
|  | TBRG4 |  |
|  | FANCG |  |
|  | BLM |  |
|  | MRPL1 |  |
|  | ACLY |  |
|  | PWP1 |  |
|  | DEPDC1B |  |
|  | SEH1L |  |
|  | NOP56 |  |
|  | STIL |  |
|  | MAPRE1 |  |
|  | PSMC3IP |  |
|  | CEBPZ |  |
|  | METAP1 |  |
|  | UTP6 |  |
|  | PPP1CC |  |
|  | TK1 |  |
|  | EIF4A1 |  |
|  | CUL2 |  |
|  | PPID |  |
|  | PRMT5 |  |
|  | MPHOSPH9 |  |
|  | RFC4 |  |
|  | UBE2S |  |
|  | SAP130 |  |
|  | SBNO1 |  |
|  | METTL8 |  |
|  | MCM3 |  |
|  | GABPB1 |  |
|  | GMNN |  |
|  | SRPK1 |  |
|  | PTBP1 |  |
|  | HAT1 |  |
|  | MRPL42 |  |
|  | NOP2 |  |
|  | ZW10 |  |
|  | HNRNPK |  |
|  | PSRC1 |  |
|  | WDR74 |  |
|  | C13orf34 |  |
|  | DNAJC7 |  |
|  | PRMT3 |  |
|  | CDC123 |  |
|  | GANAB |  |
|  | HNRNPA2B1 |  |
|  | SLC7A1 |  |
|  | C9orf100 |  |
|  | ZNF367 |  |
|  | HNRNPA3 |  |
|  | MLF1IP |  |
|  | RPL7L1 |  |
|  | EIF5AL1 |  |
|  | GINS1 |  |
|  | DNAJC11 |  |
|  | BMS1 |  |
|  | MPHOSPH10 |  |
|  | KIAA0101 |  |
|  | MRPL30 |  |
|  | DNAJA1 |  |
|  | PSMD14 |  |
|  | MAPKAPK5 |  |
|  | POLE2 |  |
|  | PDCD11 |  |
|  | RNF4 |  |
|  | AGPS |  |
|  | DCUN1D5 |  |
|  | POLD2 |  |
|  | DSP |  |
|  | KRR1 |  |
|  | G3BP1 |  |
|  | FUBP1 |  |
|  | EXOSC3 |  |
|  | TTC27 |  |
|  | USP46 |  |
|  | ZC3H15 |  |
|  | GCN1L1 |  |
|  | ZC3HAV1L |  |
|  | DDX55 |  |
|  | CEP78 |  |
|  | KHSRP |  |
|  | DNA2 |  |
|  | MDH2 |  |
|  | CDC27 |  |
|  | GINS2 |  |
|  | ASF1B |  |
|  | SCFD2 |  |
|  | PRIM1 |  |
|  | ESF1 |  |
|  | EIF4A3 |  |
|  | MDC1 |  |
|  | ABCF1 |  |
|  | TARDBP |  |
|  | C6orf167 |  |
|  | SMARCAD1 |  |
|  | TRA2B |  |
|  | XPOT |  |
|  | MTCH2 |  |
|  | DNAH14 |  |
|  | MTMR2 |  |
|  | TUBB |  |
|  | KIAA1429 |  |
|  | ZNF695 |  |
|  | ATAD3A |  |
|  | PIGW |  |
|  | C10orf2 |  |
|  | LTV1 |  |
|  | SLC25A32 |  |
|  | NEDD1 |  |
|  | MTPAP |  |
|  | TMEM194A |  |
|  | NAT10 |  |
|  | EPT1 |  |
|  | CENPL |  |
|  | SEPHS1 |  |
|  | SCLT1 |  |
|  | EZH2 |  |
|  | NKRF |  |
|  | RQCD1 |  |
|  | FXR1 |  |
|  | HSPA9 |  |
|  | C10orf18 |  |
|  | FADS1 |  |
|  | BBS7 |  |
|  | KNTC1 |  |
|  | PPIL5 |  |
|  | SCO1 |  |
|  | E2F8 |  |
|  | GPI |  |
|  | STRAP |  |
|  | AIMP1 |  |
|  | HDAC2 |  |
|  | GSTCD |  |
|  | MPP6 |  |
|  | WRNIP1 |  |
|  | GEMIN5 |  |
|  | NAA50 |  |
|  | PLAA |  |
|  | NAA35 |  |
|  | SLC5A6 |  |
|  | XPO1 |  |
|  | E2F3 |  |
|  | YWHAQ |  |
|  | MRPS9 |  |
|  | PTDSS1 |  |
|  | MCM7 |  |
|  | SFRS9 |  |
|  | SENP1 |  |
|  | AFG3L2 |  |
|  | SMN2 |  |
|  | DCAF16 |  |
|  | RAN |  |
|  | RNASEH2A |  |
|  | CDCA4 |  |
|  | AHCTF1 |  |
|  | CENPW |  |
|  | LIN9 |  |
|  | CS |  |
|  | N4BP2 |  |
|  | MRPS30 |  |
|  | VPS33A |  |
|  | YME1L1 |  |
|  | LOC221710 |  |
|  | TUBA1C |  |
|  | HNRNPA3P1 |  |
|  | C9orf40 |  |
|  | RRP15 |  |
|  | ADNP2 |  |
|  | MCM8 |  |
|  | RAD21 |  |
|  | FOXK2 |  |
|  | MRPS18C |  |
|  | HNRNPU |  |
|  | CPSF2 |  |
|  | GINS3 |  |
|  | FAM98B |  |
|  | OTUD6B |  |
|  | RAD18 |  |
|  | C2orf3 |  |
|  | TPRKB |  |
|  | C4orf46 |  |
|  | NOC2L |  |
|  | RPF2 |  |
|  | FKBP4 |  |
|  | LBR |  |
|  | CTPS |  |
|  | GTF3C2 |  |
|  | DSN1 |  |
|  | SAAL1 |  |
|  | ANKRD17 |  |
|  | SUZ12 |  |
|  | C16orf87 |  |
|  | PHB |  |
|  | NME1 |  |
|  | E2F1 |  |
|  | AASDHPPT |  |
|  | CCDC138 |  |
|  | HNRNPM |  |
|  | CEP152 |  |
|  | E2F7 |  |
|  | NARS |  |
|  | SIGMAR1 |  |
|  | CDC7 |  |
|  | MRPS5 |  |
|  | QTRTD1 |  |
|  | IPO4 |  |
|  | ZC3HC1 |  |
|  | PTPN11 |  |
|  | YEATS2 |  |
|  | TARS |  |
|  | RFC2 |  |
|  | NUP54 |  |
|  | KIAA0090 |  |
|  | CDK1 |  |
|  | CCDC85C |  |
|  | ZRANB3 |  |
|  | RCC2 |  |
|  | WDR4 |  |
|  | PSMD1 |  |
|  | NUP155 |  |
|  | NDUFS1 |  |
|  | TPI1 |  |
|  | YWHAZ |  |
|  | TIMM8A |  |
|  | XRCC5 |  |
|  | GEN1 |  |
|  | RBM17 |  |
|  | RAD23B |  |
|  | EIF2B1 |  |
|  | UTP15 |  |
|  | DHX9 |  |
|  | YES1 |  |
|  | RFC1 |  |
|  | HSP90AB1 |  |
|  | GAPDH |  |
|  | AP1AR |  |
|  | EIF3B |  |
|  | TUBA1B |  |
|  | PGM2 |  |
|  | BRIX1 |  |
|  | SMC6 |  |
|  | HMBS |  |
|  | LCLAT1 |  |
|  | NUP93 |  |
|  | RBM12 |  |
|  | GTF3C3 |  |
|  | PGAM1 |  |
|  | DNTTIP2 |  |
|  | POLD3 |  |
|  | YY1 |  |
|  | PARP1 |  |
|  | MYO19 |  |
|  | SUPT16H |  |
|  | CIRH1A |  |
|  | ARL5B |  |
|  | DFFA |  |
|  | MND1 |  |
|  | CACYBP |  |
|  | SMC3 |  |
|  | NUFIP1 |  |
|  | METTL2A |  |
|  | SYNCRIP |  |
|  | KPNA1 |  |
|  | TROAP |  |
|  | SNRNP27 |  |
|  | MRPL37 |  |
|  | FANCM |  |
|  | ACP1 |  |
|  | MARS2 |  |
|  | UBAP2 |  |
|  | EXOSC10 |  |
|  | DNAJC14 |  |
|  | WDR5 |  |
|  | SLC38A1 |  |
|  | GTF2H3 |  |
|  | CYCS |  |
|  | CBX1 |  |
|  | CCNJ |  |
|  | HSPA14 |  |
|  | DCAF17 |  |
|  | SMPD4 |  |
|  | HSPA4L |  |
|  | SART3 |  |
|  | UBXN2A |  |
|  | RSRC1 |  |
|  | CBS |  |
|  | PDXP |  |
|  | C18orf19 |  |
|  | FTSJ3 |  |
|  | USP1 |  |
|  | UMPS |  |
|  | SR140 |  |
|  | MINA |  |
|  | EIF1AD |  |
|  | NRAS |  |
|  | PFN2 |  |
|  | GFM1 |  |
|  | MRPS22 |  |
|  | EIF4G1 |  |
|  | IPO9 |  |
|  | PPIL1 |  |
|  | C9orf30 |  |
|  | CCDC21 |  |
|  | SRM |  |
|  | SFPQ |  |
|  | DHX57 |  |
|  | CEP76 |  |
|  | HDGF |  |
|  | OPA1 |  |
|  | PTTG1 |  |
|  | SNRNP200 |  |
|  | FAM111B |  |
|  | SMNDC1 |  |
|  | TBP |  |
|  | TAF1A |  |
|  | C7orf29 |  |
|  | ZNF286A |  |
|  | HMGA1 |  |
|  | ERAL1 |  |
|  | KIAA0020 |  |
|  | VCP |  |
|  | PES1 |  |
|  | EIF2AK2 |  |
|  | CHD7 |  |
|  | SAPS3 |  |
|  | MDH1 |  |
|  | LARS2 |  |
|  | NOC3L |  |
|  | SETD8 |  |
|  | WBP11 |  |
|  | FASTKD2 |  |
|  | RBM14 |  |
|  | DDX47 |  |
|  | SNRPE |  |
|  | AIMP2 |  |
|  | SUV39H1 |  |
|  | TACO1 |  |
|  | EIF5A |  |
|  | BRI3BP |  |
|  | STAMBP |  |
|  | UBE2R2 |  |
|  | LARP1B |  |
|  | RBMX |  |
|  | E2F6 |  |
|  | FASTKD1 |  |
|  | TRIM59 |  |
|  | RECQL4 |  |
|  | GARS |  |
|  | KPNA4 |  |
|  | HYLS1 |  |
|  | DDX1 |  |
|  | NLE1 |  |
|  | LOC441089 |  |
|  | BAT2 |  |
|  | EPRS |  |
|  | STOML2 |  |
|  | C2orf69 |  |
|  | POLR3A |  |
|  | CBX3 |  |
|  | SKP2 |  |
|  | USP10 |  |
|  | SAE1 |  |
|  | YKT6 |  |
|  | LRRC42 |  |
|  | IPO5 |  |
|  | USP5 |  |
|  | TAF2 |  |
|  | RG9MTD1 |  |
|  | PPP4R2 |  |
|  | HAUS3 |  |
|  | NUP160 |  |
|  | SLC4A1AP |  |
|  | SMU1 |  |
|  | CDC5L |  |
|  | PITPNB |  |
|  | WASF1 |  |
|  | METTL2B |  |
|  | ILF2 |  |
|  | PPIG |  |
|  | MRE11A |  |
|  | MAZ |  |
|  | C12orf43 |  |
|  | DNAJC2 |  |
|  | NAF1 |  |
|  | GNL3 |  |
|  | FANCC |  |
|  | SMS |  |
|  | INTS2 |  |
|  | CDC23 |  |
|  | ZBTB2 |  |
|  | SNRPF |  |
|  | C12orf24 |  |
|  | THOP1 |  |
|  | SNF8 |  |
|  | MTL5 |  |
|  | UGGT1 |  |
|  | MRPL47 |  |
|  | PSMD7 |  |
|  | CDK8 |  |
|  | METTL5 |  |
|  | GUF1 |  |
|  | TMEM206 |  |
|  | RPAP3 |  |
|  | C16orf88 |  |
|  | NOL9 |  |
|  | DHFR |  |
|  | HSPE1 |  |
|  | NKIRAS2 |  |
|  | PAXIP1 |  |
|  | PSMB2 |  |
|  | GPN3 |  |
|  | RAD51C |  |
|  | SRPK2 |  |
|  | RNF214 |  |
|  | SENP3 |  |
|  | PRTFDC1 |  |
|  | QSER1 |  |
|  | VTA1 |  |
|  | DAZAP1 |  |
|  | APTX |  |
|  | PCNA |  |
|  | CISD2 |  |
|  | VDAC1 |  |
|  | ILF3 |  |
|  | PDCD2 |  |
|  | SEC23A |  |
|  | ZFP91 |  |
|  | PHF6 |  |
|  | DNMT1 |  |
|  | HOMER1 |  |
|  | ZBTB9 |  |
|  | JARID2 |  |
|  | FGFR1OP |  |
|  | SSX2IP |  |
|  | PNMA1 |  |
|  | PDCL3 |  |
|  | LMAN1 |  |
|  | NUP85 |  |
|  | GRPEL2 |  |
|  | FAM199X |  |
|  | NONO |  |
|  | TOMM40 |  |
|  | SMYD5 |  |
|  | C17orf71 |  |
|  | LDHA |  |
|  | CNOT10 |  |
|  | DPH2 |  |
|  | NCBP2 |  |
|  | BAG2 |  |
|  | LRRC40 |  |
|  | RGP1 |  |
|  | RARS |  |
|  | LOC727896 |  |
|  | MRFAP1 |  |
|  | YRDC |  |
|  | KIF5B |  |
|  | ATP5B |  |
|  | PRDM4 |  |
|  | RRP9 |  |
|  | GAS2L3 |  |
|  | PSME4 |  |
|  | NIP7 |  |
|  | HBS1L |  |
|  | RPUSD4 |  |
|  | TTPAL |  |
|  | RBM27 |  |
|  | NUDT15 |  |
|  | UTP11L |  |
|  | C2orf49 |  |
|  | CCAR1 |  |
|  | HCFC1 |  |
|  | C17orf53 |  |
|  | TAF9 |  |
|  | SNW1 |  |
|  | EBNA1BP2 |  |
|  | PSIMCT-1 |  |
|  | CSNK2A1P |  |
|  | SMARCC1 |  |
|  | TMEM209 |  |
|  | TNPO3 |  |
|  | RRP12 |  |
|  | UTP20 |  |
|  | FANCA |  |
|  | ENO1 |  |
|  | YARS |  |
|  | EIF4H |  |
|  | C11orf84 |  |
|  | ISG20L2 |  |
|  | UTP14A |  |
|  | C1orf109 |  |
|  | NSUN2 |  |
|  | C19orf48 |  |
|  | NUP43 |  |
|  | HAUS2 |  |
|  | LONP1 |  |
|  | BAZ1B |  |
|  | GOT2 |  |
|  | VRK2 |  |
|  | RDX |  |
|  | STMN1 |  |
|  | RPE |  |
|  | TLK2 |  |
|  | C5orf34 |  |
|  | C6orf153 |  |
|  | CDC73 |  |
|  | KIF24 |  |
|  | DHCR7 |  |
|  | C12orf32 |  |
|  | DTYMK |  |
|  | TRIM28 |  |
|  | SF3B3 |  |
|  | GPATCH4 |  |
|  | C17orf96 |  |
|  | GNPNAT1 |  |
|  | CLNS1A |  |
|  | LIG3 |  |
|  | LUC7L2 |  |
|  | SNRPC |  |
|  | ANP32A |  |
|  | TNPO1 |  |
|  | TUBGCP4 |  |
|  | STT3A |  |
|  | RNF34 |  |
|  | AATF |  |
|  | UBE2C |  |
|  | UBE2G1 |  |
|  | TET3 |  |
|  | ALKBH2 |  |
|  | TMEM33 |  |
|  | BARD1 |  |
|  | TMEM185B |  |
|  | NUP35 |  |
|  | SPC24 |  |
|  | SUPV3L1 |  |
|  | ACTL6A |  |
|  | ZNF639 |  |
|  | SMC4 |  |
|  | CWC27 |  |
|  | HNRNPH3 |  |
|  | SLC7A5 |  |
|  | PAFAH1B2 |  |
|  | SPATA5 |  |
|  | INTS7 |  |
|  | C12orf11 |  |
|  | NFKBIL2 |  |
|  | ARMC1 |  |
|  | MRPL12 |  |
|  | LSG1 |  |
|  | PHF19 |  |
|  | FIGNL1 |  |
|  | GNL2 |  |
|  | C10orf12 |  |
|  | REXO4 |  |
|  | ARCN1 |  |
|  | TSSC1 |  |
|  | CPSF7 |  |
|  | TMTC3 |  |
|  | SPATS2 |  |
|  | KARS |  |
|  | GLRX5 |  |
|  | HTRA2 |  |
|  | PGAM4 |  |
|  | SF3A3 |  |
|  | RPP30 |  |
|  | RAB6A |  |
|  | TAF11 |  |
|  | DCUN1D4 |  |
|  | PSMB5 |  |
|  | CDV3 |  |
|  | RPS6KC1 |  |
|  | TRAP1 |  |
|  | BOP1 |  |
|  | ARL6IP6 |  |
|  | ASAP1 |  |
|  | C14orf106 |  |
|  | ACTR6 |  |
|  | POFUT1 |  |
|  | PWP2 |  |
|  | SUV420H1 |  |
|  | MFN1 |  |
|  | CSNK2A1 |  |
|  | FUBP3 |  |
|  | DDX27 |  |
|  | POLA1 |  |
|  | MED17 |  |
|  | TBC1D1 |  |
|  | NDUFAF4 |  |
|  | DDX39 |  |
|  | AURKAPS1 |  |
|  | FIGN |  |
|  | BZW2 |  |
|  | BUD13 |  |
|  | SF3B14 |  |
|  | NUDT21 |  |
|  | PRPS1 |  |
|  | WHSC2 |  |
|  | HSP90AA1 |  |
|  | SENP2 |  |
|  | CHIC2 |  |
|  | ORC2L |  |
|  | CHRAC1 |  |
|  | SNRNP48 |  |
|  | INTS8 |  |
|  | VBP1 |  |
|  | CCDC15 |  |
|  | TMX2 |  |
|  | TIMM17A |  |
|  | PCBP1 |  |
|  | C10orf119 |  |
|  | ALG8 |  |
|  | C14orf33 |  |
|  | ZNF207 |  |
|  | HEATR2 |  |
|  | NETO2 |  |
|  | CLP1 |  |
|  | RTKN |  |
|  | SNRNP40 |  |
|  | ALS2CR4 |  |
|  | IPPK |  |
|  | TERF1 |  |
|  | CCDC137 |  |
|  | SMC1A |  |
|  | SAMD1 |  |
|  | AQR |  |
|  | SFRS2 |  |
|  | UTP3 |  |
|  | TMEM201 |  |
|  | PAK2 |  |
|  | RBM15 |  |
|  | GPR19 |  |
|  | EIF2C3 |  |
|  | CD3EAP |  |
|  | AGFG1 |  |
|  | CKS1B |  |
|  | TADA2A |  |
|  | MEST |  |
|  | CCDC34 |  |
|  | ZNF623 |  |
|  | EED |  |
|  | ATP6V1C1 |  |
|  | UPF3B |  |
|  | MYBBP1A |  |
|  | RECQL |  |
|  | CSTF3 |  |
|  | RBM19 |  |
|  | SMARCA5 |  |
|  | GNL3L |  |
|  | UHRF1BP1 |  |
|  | AHSA1 |  |
|  | CBX5 |  |
|  | RANGAP1 |  |
|  | POLR1E |  |
|  | UBXN7 |  |
|  | MRPL11 |  |
|  | PSMG1 |  |
|  | LARP4 |  |
|  | TAF1B |  |
|  | PPP2R1B |  |
|  | C17orf75 |  |
|  | MAP6D1 |  |
|  | NUP98 |  |
|  | RMI1 |  |
|  | NUP188 |  |
|  | NMD3 |  |
|  | C15orf41 |  |
|  | SRRD |  |
|  | LOC100128191 |  |
|  | POLE |  |
|  | TOMM5 |  |
|  | ECE2 |  |
|  | WDR36 |  |
|  | MAPK6 |  |
|  | PSAT1 |  |
|  | HNRNPAB |  |
|  | NASP |  |
|  | EIF3A |  |
|  | C2orf43 |  |
|  | EXOC5 |  |
|  | UBE4A |  |
|  | OXSR1 |  |
|  | VDAC2 |  |
|  | TSN |  |
|  | TWISTNB |  |
|  | C18orf55 |  |
|  | FARSA |  |
|  | METAP2 |  |
|  | PDHX |  |
|  | MRPL51 |  |
|  | NRD1 |  |
|  | ZNF473 |  |
|  | LRRC58 |  |
|  | DCAF7 |  |
|  | UBQLN1 |  |
|  | LOC401010 |  |
|  | SSBP1 |  |
|  | SKA2 |  |
|  | CEP250 |  |
|  | IGF2BP3 |  |
|  | FAM189B |  |
|  | IQGAP3 |  |
|  | TFAM |  |
|  | NAE1 |  |
|  | RRP1 |  |
|  | KCMF1 |  |
|  | C20orf20 |  |
|  | ADAM17 |  |
|  | HNRNPA1L2 |  |
|  | ATP13A3 |  |
|  | PCGF6 |  |
|  | C6orf150 |  |
|  | MRPL16 |  |
|  | CENPQ |  |
|  | NUDT1 |  |
|  | USP42 |  |
|  | ZNF146 |  |
|  | C1QBP |  |
|  | RUVBL2 |  |
|  | C11orf57 |  |
|  | CWC22 |  |
|  | KLC2 |  |
|  | SFXN4 |  |
|  | MYL6B |  |
|  | SENP5 |  |
|  | HUWE1 |  |
|  | GTF2F2 |  |
|  | SPIN4 |  |
|  | COPS8 |  |
|  | ANKIB1 |  |
|  | MAP4K4 |  |
|  | WAC |  |
|  | EIF3M |  |
|  | HNRNPUL2 |  |
|  | DNMT3A |  |
|  | PRMT1 |  |
|  | WDR77 |  |
|  | LIG1 |  |
|  | NBN |  |
|  | COIL |  |
|  | SOX12 |  |
|  | MEN1 |  |
|  | C4orf21 |  |
|  | PDS5A |  |
|  | TMEM199 |  |
|  | PARD3 |  |
|  | EXOC2 |  |
|  | MRPL15 |  |
|  | MAGOHB |  |
|  | ZNF384 |  |
|  | MYC |  |
|  | QSOX2 |  |
|  | PUS3 |  |
|  | ZNF544 |  |
|  | LRP12 |  |
|  | RDM1 |  |
|  | CTNNBL1 |  |
|  | GNAI3 |  |
|  | SFRS13A |  |
|  | DCLRE1B |  |
|  | USP37 |  |
|  | MARS |  |
|  | PPP2R5E |  |
|  | C12orf73 |  |
|  | INTS6 |  |
|  | GSS |  |
|  | FBXO30 |  |
|  | MTERFD1 |  |
|  | PSMC5 |  |
|  | MORF4L2 |  |
|  | OBFC2B |  |
|  | SMCR7L |  |
|  | BRAP |  |
|  | UBE2N |  |
|  | PMS1 |  |
|  | DDX56 |  |
|  | PELP1 |  |
|  | AHCY |  |
|  | SLC25A13 |  |
|  | NOP16 |  |
|  | MTA3 |  |
|  | SFRS7 |  |
|  | ZNF326 |  |
|  | G2E3 |  |
|  | SCFD1 |  |
|  | POLDIP2 |  |
|  | SSR1 |  |
|  | SP3 |  |
|  | UBTF |  |
|  | RSL1D1 |  |
|  | CCDC59 |  |
|  | YWHAE |  |
|  | RBM12B |  |
|  | CSTF2 |  |
|  | GPSM2 |  |
|  | FAM98A |  |
|  | MAK16 |  |
|  | TRMT6 |  |
|  | PARG |  |
|  | PSMB7 |  |
|  | UBE2O |  |
|  | MTA1 |  |
|  | RPAP2 |  |
|  | NEIL3 |  |
|  | PTGES3 |  |
|  | PUS1 |  |
|  | VDAC3 |  |
|  | C13orf37 |  |
|  | PREB |  |
|  | PPME1 |  |
|  | HPRT1 |  |
|  | CSTF1 |  |
|  | LOC550112 |  |
|  | FAM104A |  |
|  | EI24 |  |
|  | ZMYND19 |  |
|  | LEO1 |  |
|  | CFL1 |  |
|  | UBE2MP1 |  |
|  | LZIC |  |
|  | GMCL1 |  |
|  | ACAT2 |  |
|  | MTRF1L |  |
|  | DDX54 |  |
|  | ADSL |  |
|  | KIAA0947 |  |
|  | FAM161A |  |
|  | HMGXB4 |  |
|  | FAM20B |  |
|  | C1orf96 |  |
|  | KIAA1586 |  |
|  | UPF2 |  |
|  | SHMT2 |  |
|  | GTF2E1 |  |
|  | MRPL35 |  |
|  | PFKM |  |
|  | NUS1 |  |
|  | LSM5 |  |
|  | CCDC112 |  |
|  | ATP2B1 |  |
|  | POLE3 |  |
|  | HYOU1 |  |
|  | TGIF2 |  |
|  | C1orf103 |  |
|  | UCHL3 |  |
|  | PPP1R14B |  |
|  | WDR53 |  |
|  | ACTG1 |  |
|  | FAF1 |  |
|  | XRCC3 |  |
|  | RDBP |  |
|  | RNASEN |  |
|  | CBL |  |
|  | PA2G4P4 |  |
|  | LUZP6 |  |
|  | NCOA6 |  |
|  | AVL9 |  |
|  | DYNC1LI1 |  |
|  | FKBP3 |  |
|  | TGS1 |  |
|  | RAE1 |  |
|  | CTDSPL2 |  |
|  | LARS |  |
|  | HIRA |  |
|  | PARP2 |  |
|  | EHBP1 |  |
|  | MFSD2B |  |
|  | IPO7 |  |
|  | DBN1 |  |
|  | PPP2R5D |  |
|  | TIMM44 |  |
|  | CEP57 |  |
|  | ACOT7 |  |
|  | KIF2A |  |
|  | CSNK2B |  |
|  | CCNK |  |
|  | COPB2 |  |
|  | NIF3L1 |  |
|  | SNAPC3 |  |
|  | COPS7B |  |
|  | EIF2A |  |
|  | SMARCAL1 |  |
|  | CBLL1 |  |
|  | TAF1D |  |
|  | DNAJB11 |  |
|  | CFL2 |  |
|  | TFDP2 |  |
|  | SPATA5L1 |  |
|  | YARS2 |  |
|  | SND1 |  |
|  | UBR5 |  |
|  | UBA2 |  |
|  | ZFR |  |
|  | NUP62 |  |
|  | IMP4 |  |
|  | CSNK1G1 |  |
|  | SLC25A10 |  |
|  | AGBL5 |  |
|  | SLC16A1 |  |
|  | FAM168B |  |
|  | DDX46 |  |
|  | GTPBP10 |  |
|  | SLC25A33 |  |
|  | PDK1 |  |
|  | SLC25A3 |  |
|  | COX10 |  |
|  | CEP72 |  |
|  | GNB1 |  |
|  | SYCE2 |  |
|  | TTC4 |  |
|  | USP39 |  |
|  | MAEA |  |
|  | ABL2 |  |
|  | C9orf41 |  |
|  | ISY1 |  |
|  | MED1 |  |
|  | STC2 |  |
|  | COX4NB |  |
|  | KPNA3 |  |
|  | DYNC1H1 |  |
|  | C17orf80 |  |
|  | HNRPLL |  |
|  | MYBL1 |  |
|  | ACTR3B |  |
|  | LOC643387 |  |
|  | ERI1 |  |
|  | MTOR |  |
|  | MMACHC |  |
|  | RALA |  |
|  | RPA1 |  |
|  | PPIF |  |
|  | SLC30A6 |  |
|  | POLR2H |  |
|  | ALG3 |  |
|  | STRN |  |
|  | NDRG3 |  |
|  | C1orf107 |  |
|  | CEP63 |  |
|  | CCNE2 |  |
|  | PTRH2 |  |
|  | MTX2 |  |
|  | ARMC8 |  |
|  | STYX |  |
|  | NUP50 |  |
|  | ATL3 |  |
|  | DIP2B |  |
|  | CRKL |  |
|  | TOP3A |  |
|  | C14orf145 |  |
|  | KIAA0586 |  |
|  | ANKRD52 |  |
|  | GRWD1 |  |
|  | C7orf49 |  |
|  | SRRT |  |
|  | PFAS |  |
|  | U2AF2 |  |
|  | ATF2 |  |
|  | FAM175B |  |
|  | SART1 |  |
|  | SMG7 |  |
|  | NOM1 |  |
|  | EIF2B3 |  |
|  | TFG |  |
|  | DPP3 |  |
|  | NOL6 |  |
|  | FYTTD1 |  |
|  | INTS4 |  |
|  | CHAC2 |  |
|  | RPS6KB1 |  |
|  | TOMM34 |  |
|  | DUS4L |  |
|  | TLK1 |  |
|  | PHAX |  |
|  | XPO6 |  |
|  | PIGU |  |
|  | SNRPA |  |
|  | DRG1 |  |
|  | PPAN |  |
|  | PSMA2 |  |
|  | HNRNPF |  |
|  | ZFP64 |  |
|  | AMMECR1 |  |
|  | TRUB1 |  |
|  | UBE2L3 |  |
|  | SLC2A1 |  |
|  | RBM45 |  |
|  | C1orf124 |  |
|  | RALBP1 |  |
|  | KLHL23 |  |
|  | RBBP4 |  |
|  | MED6 |  |
|  | HAUS8 |  |
|  | PLEKHA8 |  |
|  | DLD |  |
|  | UBE3C |  |
|  | ATP2A2 |  |
|  | IRAK1 |  |
|  | MCART1 |  |
|  | NIPA2 |  |
|  | KIF1B |  |
|  | CKS2 |  |
|  | FH |  |
|  | CLASP1 |  |
|  | STAU1 |  |
|  | AZIN1 |  |
|  | ADNP |  |
|  | PSPC1 |  |
|  | ACTR3 |  |
|  | ZCCHC4 |  |
|  | DDX52 |  |
|  | SMG5 |  |
|  | ZNF280B |  |
|  | IWS1 |  |
|  | CNO |  |
|  | PPP1R12A |  |
|  | PSIP1 |  |
|  | SPAST |  |
|  | NFS1 |  |
|  | RIC8B |  |
|  | RG9MTD2 |  |
|  | UBE2E3 |  |
|  | DVL2 |  |
|  | ERCC3 |  |
|  | MGA |  |
|  | RWDD4A |  |
|  | ARL6IP1 |  |
|  | SIP1 |  |
|  | STAG1 |  |
|  | ESRP1 |  |
|  | NMT1 |  |
|  | RAB10 |  |
|  | MCM3APAS |  |
|  | GMEB1 |  |
|  | RCCD1 |  |
|  | GPN1 |  |
|  | RELA |  |
|  | PTCD1 |  |
|  | FBXL18 |  |
|  | FOSL1 |  |
|  | BYSL |  |
|  | CELF1 |  |
|  | MRPS10 |  |
|  | GLMN |  |
|  | C8orf33 |  |
|  | GATC |  |
|  | UCHL1 |  |
|  | SFRS3 |  |
|  | SUGT1 |  |
|  | CAPRIN1 |  |
|  | HSF1 |  |
|  | LAS1L |  |
|  | PSMC3 |  |
|  | CHD8 |  |
|  | HS2ST1 |  |
|  | CENPJ |  |
|  | KIAA0368 |  |
|  | CDK16 |  |
|  | ATXN7L3 |  |
|  | ZSCAN20 |  |
|  | CCDC51 |  |
|  | IFRD1 |  |
|  | NSDHL |  |
|  | PYGL |  |
|  | MEMO1 |  |
|  | PLOD2 |  |
|  | FAM91A1 |  |
|  | MAPK8 |  |
|  | RTCD1 |  |
|  | GATSL1 |  |
|  | SLC35F2 |  |
|  | MMADHC |  |
|  | CHUK |  |
|  | NUDT5 |  |
|  | ZNF770 |  |
|  | SDCCAG3 |  |
|  | DNAJA3 |  |
|  | TMEM135 |  |
|  | UBE2V1 |  |
|  | POLD1 |  |
|  | RAP1GDS1 |  |
|  | TRMT61B |  |
|  | RIPK2 |  |
|  | MPP2 |  |
|  | ZBTB39 |  |
|  | SF1 |  |
|  | HTT |  |
|  | DCUN1D1 |  |
|  | GPD2 |  |
|  | ORC5L |  |
|  | PUF60 |  |
|  | DNMT3B |  |
|  | TRIP12 |  |
|  | PPIAL4C |  |
|  | NFRKB |  |
|  | APEX1 |  |
|  | COPG2 |  |
|  | VARS |  |
|  | C5orf22 |  |
|  | C10orf88 |  |
|  | JMJD6 |  |
|  | C20orf27 |  |
|  | MCM5 |  |
|  | CBWD1 |  |
|  | ATPBD4 |  |
|  | YBX2 |  |
|  | RTN3 |  |
|  | PPP1R8 |  |
|  | RNF2 |  |
|  | SRP68 |  |
|  | C17orf58 |  |
|  | POM121 |  |
|  | MRPL10 |  |
|  | KIAA1715 |  |
|  | HSPA8 |  |
|  | CASP8AP2 |  |
|  | PPP3R1 |  |
|  | DHX8 |  |
|  | REPS1 |  |
|  | C2orf47 |  |
|  | ZUFSP |  |
|  | TRMT5 |  |
|  | CDYL |  |
|  | ZC3HAV1 |  |
|  | SPIN1 |  |
|  | C3orf37 |  |
|  | MRPL44 |  |
|  | CEP97 |  |
|  | CHCHD8 |  |
|  | ZCCHC8 |  |
|  | DDX23 |  |
|  | UBIAD1 |  |
|  | PGM3 |  |
|  | SLC38A7 |  |
|  | PFKP |  |
|  | THRAP3 |  |
|  | ZNF239 |  |
|  | USP32 |  |
|  | GMFB |  |
|  | IMPAD1 |  |
|  | STRN3 |  |
|  | ATP5G3 |  |
|  | SGTA |  |
|  | HOOK1 |  |
|  | C20orf72 |  |
|  | DOCK7 |  |
|  | B3GNT5 |  |
|  | PGBD1 |  |
|  | PRKAA2 |  |
|  | PALB2 |  |
|  | PAPOLA |  |
|  | HSP90AB2P |  |
|  | KIAA0114 |  |
|  | ESCO1 |  |
|  | MTAP |  |
|  | UBFD1 |  |
|  | DNM1L |  |
|  | C16orf75 |  |
|  | PVR |  |
|  | MTFR1 |  |
|  | PM20D2 |  |
|  | SAP30 |  |
|  | PHF10 |  |
|  | DBR1 |  |
|  | COQ2 |  |
|  | CIAO1 |  |
|  | FUS |  |
|  | SNAPC1 |  |
|  | RPAP1 |  |
|  | CBX2 |  |
|  | RBBP7 |  |
|  | RAD50 |  |
|  | RSF1 |  |
|  | TPR |  |
|  | KTN1 |  |
|  | SAFB |  |
|  | SMARCD1 |  |
|  | PTMA |  |
|  | PIK3R4 |  |
|  | SCYL2 |  |
|  | 6-Mar |  |
|  | VANGL1 |  |
|  | PIGX |  |
|  | GTF2H2 |  |
|  | DHX16 |  |
|  | FBXO22OS |  |
|  | GPRIN1 |  |
|  | DYNLL1 |  |
|  | TAF3 |  |
|  | ASXL1 |  |
|  | ZNF620 |  |
|  | USO1 |  |
|  | PFKFB4 |  |
|  | BEND3 |  |
|  | DCK |  |
|  | ZYG11A |  |
|  | SMARCB1 |  |
|  | COPS5 |  |
|  | PARL |  |
|  | COPG |  |
|  | SNRPD3 |  |
|  | TCF19 |  |
|  | TBC1D16 |  |
|  | C14orf104 |  |
|  | CHEK2 |  |
|  | KBTBD2 |  |
|  | COMMD2 |  |
|  | RRS1 |  |
|  | PPT2 |  |
|  | SUMO2 |  |
|  | FBL |  |
|  | TRMT12 |  |
|  | CASP2 |  |
|  | POLR3D |  |
|  | ZAK |  |
|  | C6orf182 |  |
|  | NAA40 |  |
|  | PPRC1 |  |
|  | H2AFV |  |
|  | KDM1B |  |
|  | AGPAT5 |  |
|  | RNF8 |  |
|  | PANX1 |  |
|  | KDELC2 |  |
|  | KIAA1958 |  |
|  | REST |  |
|  | DHX30 |  |
|  | PHKA1 |  |
|  | DVL3 |  |
|  | ZKSCAN5 |  |
|  | KIAA1549 |  |
|  | ABCF3 |  |
|  | DNAJC13 |  |
|  | PCCB |  |
|  | KIN |  |
|  | TMEM189 |  |
|  | TNPO2 |  |
|  | DDX20 |  |
|  | ENAH |  |
|  | C14orf80 |  |
|  | SYNJ2 |  |
|  | U2AF1 |  |
|  | AARSD1 |  |
|  | TIMM10 |  |
|  | GTF3C4 |  |
|  | TMEM177 |  |
|  | PHF20L1 |  |
|  | GTF2IRD1 |  |
|  | MIB1 |  |
|  | AK3L1 |  |
|  | NUP88 |  |
|  | DPF2 |  |
|  | HARS |  |
|  | SRP54 |  |
|  | DCAF10 |  |
|  | ERC1 |  |
|  | DAP3 |  |
|  | LOC144438 |  |
|  | MAP1D |  |
|  | VAPB |  |
|  | COQ3 |  |
|  | ARHGAP19 |  |
|  | DYNC1I2 |  |
|  | TMEM194B |  |
|  | CALU |  |
|  | MAP7D3 |  |
|  | POLR3B |  |
|  | ABCB7 |  |
|  | PLEKHA9 |  |
|  | GPATCH2 |  |
|  | BRD4 |  |
|  | YBX1 |  |
|  | PIF1 |  |
|  | OGFOD1 |  |
|  | KLHL5 |  |
|  | BOLA3 |  |
|  | NRM |  |
|  | ZNF670 |  |
|  | GOSR1 |  |
|  | PUS7L |  |
|  | OSGIN2 |  |
|  | NCKAP1 |  |
|  | WDR46 |  |
|  | CUL5 |  |
|  | CHD4 |  |
|  | GPR125 |  |
|  | CENPM |  |
|  | MRPL45 |  |
|  | ELOVL5 |  |
|  | KIAA0317 |  |
|  | ZNF518B |  |
|  | SFRS13B |  |
|  | ZDBF2 |  |
|  | G3BP2 |  |
|  | NHLRC2 |  |
|  | CPSF6 |  |
|  | MTDH |  |
|  | EDC3 |  |
|  | RCOR2 |  |
|  | COASY |  |
|  | PSMA4 |  |
|  | UHRF1BP1L |  |
|  | SOCS7 |  |
|  | NPEPPS |  |
|  | RCE1 |  |
|  | LDLRAD3 |  |
|  | C19orf57 |  |
|  | ERCC8 |  |
|  | SKIV2L2 |  |
|  | TXNRD1 |  |
|  | ITCH |  |
|  | XPO7 |  |
|  | MOCS3 |  |
|  | COG5 |  |
|  | ATRIP |  |
|  | FAM86C |  |
|  | CTSL2 |  |
|  | RCC1 |  |
|  | BBX |  |
|  | BAT2L2 |  |
|  | MRPS16 |  |
|  | HTATSF1 |  |
|  | DCPS |  |
|  | TMEM184C |  |
|  | HNRNPA1 |  |
|  | C10orf46 |  |
|  | PDCD2L |  |
|  | TP53BP1 |  |
|  | DIS3 |  |
|  | CARM1 |  |
|  | B3GALNT2 |  |
|  | EIF4G3 |  |
|  | TIMM22 |  |
|  | MINPP1 |  |
|  | PPP2CA |  |
|  | NT5C3 |  |
|  | CIAPIN1 |  |
|  | POM121C |  |
|  | ZNF697 |  |
|  | MOBKL1B |  |
|  | IREB2 |  |
|  | SLC36A4 |  |
|  | UTP23 |  |
|  | NUP210 |  |
|  | PL-5283 |  |
|  | SUDS3 |  |
|  | DHX35 |  |
|  | TH1L |  |
|  | FXN |  |
|  | BRCC3 |  |
|  | UBE2Z |  |
|  | USP28 |  |
|  | STAM |  |
|  | NEU3 |  |
|  | ANKLE2 |  |
|  | NUP133 |  |
|  | SLMO2 |  |
|  | GEMIN6 |  |
|  | DPM1 |  |
|  | WDR92 |  |
|  | GJC1 |  |
|  | RPTOR |  |
|  | PSMB6 |  |
|  | FADS2 |  |
|  | PYCR1 |  |
|  | OSGEPL1 |  |
|  | SLC20A1 |  |
|  | RNF219 |  |
|  | DPAGT1 |  |
|  | CHCHD4 |  |
|  | PCNXL3 |  |
|  | OTUD4 |  |
|  | TMEM69 |  |
|  | AMMECR1L |  |
|  | PEX5 |  |
|  | WDR45L |  |
|  | RBM15B |  |
|  | MLLT11 |  |
|  | TFRC |  |
|  | ALDOA |  |
|  | ETF1 |  |
|  | PHLPP1 |  |
|  | RPIA |  |
|  | LOC729020 |  |
|  | TCF3 |  |
|  | RBBP5 |  |
|  | CDCP1 |  |
|  | ZZZ3 |  |
|  | KDM3A |  |
|  | LMBR1 |  |
|  | ZNF232 |  |
|  | JRK |  |
|  | VPRBP |  |
|  | GSK3B |  |
|  | MAPK1IP1L |  |
|  | BLMH |  |
|  | APOOL |  |
|  | GRK4 |  |
|  | MAFG |  |
|  | ZNF496 |  |
|  | HADHA |  |
|  | NARS2 |  |
|  | NIPSNAP1 |  |
|  | TRIM37 |  |
|  | HPS3 |  |
|  | E2F5 |  |
|  | MSI2 |  |
|  | SRFBP1 |  |
|  | KPNA6 |  |
|  | PNPO |  |
|  | RNGTT |  |
|  | ANP32E |  |
|  | C1orf131 |  |
|  | RALGAPB |  |
|  | MEX3A |  |
|  | DCLRE1A |  |
|  | FIBP |  |
|  | FLVCR1 |  |
|  | WDR1 |  |
|  | C12orf29 |  |
|  | RNF115 |  |
|  | LAMC1 |  |
|  | GIT1 |  |
|  | C22orf30 |  |
|  | MAP3K2 |  |
|  | UBAP2L |  |
|  | EIF2AK1 |  |
|  | PRPF38A |  |
|  | IARS2 |  |
|  | FANCE |  |
|  | ZBTB12 |  |
|  | LARP7 |  |
|  | PHF20 |  |
|  | KIAA0100 |  |
|  | CUL4B |  |
|  | AKIRIN1 |  |
|  | ITSN1 |  |
|  | SOCS4 |  |
|  | RDH11 |  |
|  | BANF1 |  |
|  | SLC3A2 |  |
|  | MBTPS2 |  |
|  | LOC150786 |  |
|  | KIAA0895 |  |
|  | FAM168A |  |
|  | NDUFA12 |  |
|  | VMA21 |  |
|  | STARD7 |  |
|  | SDHA |  |
|  | IPO8 |  |
|  | EHMT2 |  |
|  | FBXL19 |  |
|  | FAM119A |  |
|  | CEBPG |  |
|  | TMX1 |  |
|  | PTBP2 |  |
|  | CCDC88A |  |
|  | RAB2A |  |
|  | KDM5A |  |
|  | ERO1L |  |
|  | NCOA3 |  |
|  | SMCHD1 |  |
|  | INTS12 |  |
|  | GNA13 |  |
|  | C7orf11 |  |
|  | MAPK1 |  |
|  | PCBP2 |  |
|  | ASCC3 |  |
|  | UBQLN4 |  |
|  | ENY2 |  |
|  | ELOVL6 |  |
|  | HSP90B1 |  |
|  | FTSJ1 |  |
|  | ZNF525 |  |
|  | ATP5C1 |  |
|  | RPF1 |  |
|  | ING1 |  |
|  | LOC93622 |  |
|  | KIAA1609 |  |
|  | CNOT1 |  |
|  | BOD1L |  |
|  | GTF2E2 |  |
|  | ATXN2L |  |
|  | MNAT1 |  |
|  | TSGA14 |  |
|  | GLRX2 |  |
|  | ATXN3 |  |
|  | TRIAP1 |  |
|  | GDI2 |  |
|  | MLF2 |  |
|  | MARK4 |  |
|  | TIGD5 |  |
|  | SMARCE1 |  |
|  | ZNF718 |  |
|  | ZNF598 |  |
|  | C14orf21 |  |
|  | HMGCR |  |
|  | RC3H2 |  |
|  | TIPRL |  |
|  | SNHG1 |  |
|  | PCMT1 |  |
|  | UBE2M |  |
|  | LOC341056 |  |
|  | NANP |  |
|  | PIAS2 |  |
|  | TFB2M |  |
|  | MRPL39 |  |
|  | FAM114A1 |  |
|  | C2CD3 |  |
|  | TPM3 |  |
|  | OSBP |  |
|  | RPRD1B |  |
|  | CAMSAP1 |  |
|  | ANKRD26 |  |
|  | HN1 |  |
|  | PTCD2 |  |
|  | CSNK1E |  |
|  | TBL1XR1 |  |
|  | ZBTB10 |  |
|  | NPM3 |  |
|  | TFCP2 |  |
|  | METTL10 |  |
|  | CLCN2 |  |
|  | DTD1 |  |
|  | C1orf74 |  |
|  | ANAPC5 |  |
|  | ADIPOR2 |  |
|  | CASP6 |  |
|  | OCRL |  |
|  | CARS |  |
|  | DLEU2 |  |
|  | SMARCA1 |  |
|  | VPS35 |  |
|  | TSC22D2 |  |
|  | NAIF1 |  |
|  | COPS3 |  |
|  | RNF160 |  |
|  | C14orf118 |  |
|  | BAG5 |  |
|  | ZNF643 |  |
|  | RBM4 |  |
|  | FAM169A |  |
|  | NME6 |  |
|  | HSPA13 |  |
|  | TAF6 |  |
|  | SFRS15 |  |
|  | FGD1 |  |
|  | TRRAP |  |
|  | STK35 |  |
|  | RANBP2 |  |
|  | TAOK1 |  |
|  | TEAD1 |  |
|  | PPP1R15B |  |
|  | ATR |  |
|  | THAP1 |  |
|  | VPS25 |  |
|  | GPS1 |  |
|  | RMND5A |  |
|  | MRPL13 |  |
|  | PSMD6 |  |
|  | C20orf117 |  |
|  | CBWD2 |  |
|  | NRBP1 |  |
|  | MRPS7 |  |
|  | ZMYM1 |  |
|  | GTPBP8 |  |
|  | UBA5 |  |
|  | PDHA1 |  |
|  | SRRM1 |  |
|  | MTMR4 |  |
|  | ERLIN1 |  |
|  | ZNF202 |  |
|  | LSM14A |  |
|  | TWF1 |  |
|  | ZCCHC7 |  |
|  | FAM53C |  |
|  | PRKRIR |  |
|  | MED28 |  |
|  | AAGAB |  |
|  | BRWD3 |  |
|  | CNP |  |
|  | MUTED |  |
|  | SUMO1 |  |
|  | SEC23IP |  |
|  | ALG9 |  |
|  | TBCCD1 |  |
|  | SLC25A15 |  |
|  | TDP1 |  |
|  | RB1CC1 |  |
|  | RFT1 |  |
|  | CREB1 |  |
|  | BRMS1L |  |
|  | KDM2B |  |
|  | SEC61A2 |  |
|  | HMGXB3 |  |
|  | CLGN |  |
|  | HDGFRP3 |  |
|  | 7-Mar |  |
|  | TOPORS |  |
|  | CAPZA1 |  |
|  | ATP6V1E2 |  |
|  | MFSD9 |  |
|  | PRR3 |  |
|  | PAIP1 |  |
|  | PSMD3 |  |
|  | RAB35 |  |
|  | SAC3D1 |  |
|  | COQ5 |  |
|  | BRD7 |  |
|  | CCNT1 |  |
|  | MSL3L2 |  |
|  | SLC30A9 |  |
|  | GTF2A1 |  |
|  | C19orf40 |  |
|  | THOC1 |  |
|  | C11orf30 |  |
|  | ICK |  |
|  | FAM60A |  |
|  | TBCA |  |
|  | HK2 |  |
|  | TNIP2 |  |
|  | ALG10 |  |
|  | LRCH3 |  |
|  | WAPAL |  |
|  | TRIB3 |  |
|  | ROCK2 |  |
|  | ATP6V0A2 |  |
|  | C5orf51 |  |
|  | TGFBRAP1 |  |
|  | DAXX |  |
|  | DLG5 |  |
|  | PRCC |  |
|  | XRCC6 |  |
|  | ARIH2 |  |
|  | SUPT3H |  |
|  | LOC728640 |  |
|  | NME2 |  |
|  | ZNF777 |  |
|  | ARID3B |  |
|  | ACVR2B |  |
|  | PHF13 |  |
|  | METTL4 |  |
|  | YTHDC1 |  |
|  | AGMAT |  |
|  | AARS |  |
|  | PDF |  |
|  | PRKRA |  |
|  | ARF6 |  |
|  | LRRC37B2 |  |
|  | ASXL2 |  |
|  | VTI1A |  |
|  | GTF3C5 |  |
|  | DUSP14 |  |
|  | TXNL4A |  |
|  | ZNF664 |  |
|  | ICMT |  |
|  | SEC24B |  |
|  | TMEM38B |  |
|  | ZNF507 |  |
|  | CCDC55 |  |
|  | ZNF143 |  |
|  | CENPP |  |
|  | ZNF749 |  |
|  | PACRGL |  |
|  | RNF168 |  |
|  | CLTC |  |
|  | RTTN |  |
|  | MRPL2 |  |
|  | SNAP47 |  |
|  | RHOBTB3 |  |
|  | MTERF |  |
|  | HIC2 |  |
|  | PSMD5 |  |
|  | ENTPD7 |  |
|  | PAQR3 |  |
|  | ZNF480 |  |
|  | SLC25A39 |  |
|  | PSMA5 |  |
|  | THUMPD3 |  |
|  | GBE1 |  |
|  | FCF1 |  |
|  | PTGES2 |  |
|  | ZMYM2 |  |
|  | PLA2G12A |  |
|  | ZNF8 |  |
|  | ZNF318 |  |
|  | C7orf70 |  |
|  | SSR3 |  |
|  | SAMD8 |  |
|  | NT5DC3 |  |
|  | ELK1 |  |
|  | ZNF300 |  |
|  | DUT |  |
|  | PHF14 |  |
|  | PFDN2 |  |
|  | ZNF48 |  |
|  | ALDH18A1 |  |
|  | AVEN |  |
|  | MCFD2 |  |
|  | MRPL9 |  |
|  | LRFN4 |  |
|  | GGH |  |
|  | VPS54 |  |
|  | MAP3K7 |  |
|  | WRAP53 |  |
|  | UBE3A |  |
|  | LOC728554 |  |
|  | TAF5L |  |
|  | UBL4A |  |
|  | BNIP3 |  |
|  | ZNF696 |  |
|  | THOC3 |  |
|  | BEND6 |  |
|  | SNRPB |  |
|  | LCORL |  |
|  | NOL8 |  |
|  | KDM2A |  |
|  | FAM193A |  |
|  | CECR5 |  |
|  | FOXRED1 |  |
|  | PANK3 |  |
|  | DIABLO |  |
|  | POLR3F |  |
|  | EIF3I |  |
|  | SAMD4B |  |
|  | SDHB |  |
|  | YDJC |  |
|  | C3orf17 |  |
|  | EXOSC8 |  |
|  | TSPAN5 |  |
|  | PIPSL |  |
|  | CUL1 |  |
|  | TMEM65 |  |
|  | LOC729082 |  |
|  | TAF13 |  |
|  | CIZ1 |  |
|  | ARHGAP21 |  |
|  | IMPDH2 |  |
|  | C1orf55 |  |
|  | CCDC77 |  |
|  | CORO1C |  |
|  | SIKE1 |  |
|  | EIF2B4 |  |
|  | EYA3 |  |
|  | PGK1 |  |
|  | SOS1 |  |
|  | KATNA1 |  |
|  | FKBPL |  |
|  | PPIA |  |
|  | ZNF699 |  |
|  | TROVE2 |  |
|  | C16orf61 |  |
|  | SGK196 |  |
|  | HEATR3 |  |
| Co-expressed genes determined by GEPIA | Co-expressed genes determined by UALCAN | Co-expressed genes determined by both GEPIA and UALCAN |
| PPAT | PPAT | PPAT |
| SRP72 | SRP72 | SRP72 |
| POLR2B | POLR2B | POLR2B |
| LYAR | LYAR | LYAR |
| NCAPG | NCAPG | NCAPG |
| WDR43 | CENPE | WDR43 |
| CCNA2 | CCNA2 | CCNA2 |
| CENPE | CHEK1 | CENPE |
| NAA15 | CDCA5 | NAA15 |
| ABCE1 | CCNB1 | ABCE1 |
| CHEK1 | FOXM1 | CHEK1 |
| PRR11 | NAA15 | PRR11 |
| CCNB1 | CDC25A | CCNB1 |
| SKA1 | FEN1 | SKA1 |
| CDCA5 | BUB1B | CDCA5 |
| BUB1 | KIF4A | BUB1 |
| CDC25A | WDR43 | CDC25A |
| PSMD12 | ABCE1 | PSMD12 |
| MCM10 | DKC1 | MCM10 |
| KIF4A | SKA1 | KIF4A |
| CKAP2L | MCM10 | CKAP2L |
| TPX2 | R3HDM1 | TPX2 |
| DEPDC1 | SGOL1 | DEPDC1 |
| MELK | KIF14 | MELK |
| FARSB | BUB1 | FARSB |
| KIAA1524 | ERCC6L | KIAA1524 |
| R3HDM1 | SSRP1 | R3HDM1 |
| BUB1B | CCDC86 | BUB1B |
| CENPO | NCAPH | CENPO |
| PA2G4 | ASPM | PA2G4 |
| FEN1 | FIP1L1 | FEN1 |
| FANCI | WHSC1 | FANCI |
| PLK1 | DEPDC1 | PLK1 |
| SGOL1 | PLK4 | SGOL1 |
| WHSC1 | TPX2 | WHSC1 |
| NOA1 | PLK1 | TTK |
| TTK | KIAA1524 | KIF14 |
| KIF14 | KPNA2 | TSR1 |
| TSR1 | DTL | KPNA2 |
| KPNA2 | TTK | ERCC6L |
| ERCC6L | MELK | FOXM1 |
| FOXM1 | URB2 | NCAPH |
| NCAPH | PRC1 | CCT8 |
| CCT8 | MCM4 | MCM4 |
| CTD-2510F5.4 | DBF4 | KIF18A |
| MCM4 | ARHGAP11A | DKC1 |
| KIF18A | PSMD12 | DBF4 |
| DKC1 | PRR11 | KIF23 |
| DBF4 | FARSB | PRPF40A |
| KIF23 | CENPO | SMC2 |
| PRPF40A | CKAP2L | DTL |
| SMC2 | C4orf14 | INCENP |
| PAICSP4 | CSE1L | KIF11 |
| DTL | TSR1 | SHCBP1 |
| INCENP | CDCA3 | ARHGAP11A |
| KIF11 | KIF23 | SSRP1 |
| NIFK | INCENP | POLR1B |
| SHCBP1 | SPAG5 | RAD51 |
| ARHGAP11A | FANCI | FIP1L1 |
| SSRP1 | UHRF1 | MAD2L1 |
| POLR1B | GSG2 | GSG2 |
| RAD51 | ESPL1 | URB2 |
| FIP1L1 | FAM136A | CSE1L |
| MAD2L1 | CCT7 | NOL10 |
| GSG2 | MKI67 | HNRNPR |
| URB2 | KIF20A | MCM6 |
| RP11-424C20.2 | NUSAP1 | HNRNPD |
| CSE1L | KIF11 | ANAPC1 |
| NOL10 | CCT8 | RNASEH1 |
| HNRNPR | XPO5 | NUP153 |
| MCM6 | MAD2L1 | CCRN4L |
| HNRNPD | EXO1 | DLGAP5 |
| ANAPC1 | NEK2 | NUSAP1 |
| RNASEH1 | C15orf42 | MTIF2 |
| NUP153 | NOL10 | UHRF1 |
| CCRN4L | C11orf82 | PRC1 |
| DLGAP5 | TOP2A | RRM2 |
| NUSAP1 | KIF18A | UBE2K |
| MTIF2 | MCM6 | NOP14 |
| UHRF1 | KPNB1 | BUB3 |
| PRC1 | NOP14 | CLSPN |
| RRM2 | DLGAP5 | ASPM |
| UBE2K | RRM2 | GMPS |
| NOP14 | HNRNPR | CCDC86 |
| BUB3 | CLSPN | CCT7 |
| CLSPN | LRPPRC | LMNB2 |
| ORC1 | CDC20 | PLK4 |
| ASPM | CASC5 | MASTL |
| GMPS | CCRN4L | DDX18 |
| CCDC86 | AURKB | CDC6 |
| CCT7 | ORC1L | DIAPH3 |
| LMNB2 | RAD51 | NEK2 |
| PLK4 | C1orf135 | EXO1 |
| MASTL | MASTL | WDR75 |
| DDX18 | FAM83D | RACGAP1 |
| CDC6 | CENPF | SPC25 |
| DDIAS | CDC6 | SLBP |
| DIAPH3 | AURKA | MKI67 |
| NEK2 | STIP1 | KPNB1 |
| EXO1 | KIF2C | CCT4 |
| WDR75 | PA2G4 | KIF20A |
| RACGAP1 | LMNB1 | CEP135 |
| SPC25 | NCL | AURKA |
| SLBP | SKA3 | TIPIN |
| MKI67 | SUV39H2 | FAM136A |
| KPNB1 | HNRNPD | H2AFZ |
| CCT4 | NUP153 | SET |
| KIF20A | LMNB2 | EIF2S1 |
| CEP135 | WDR75 | SDAD1 |
| AURKA | GART | RAD51AP1 |
| TIPIN | DDX10 | CPSF3 |
| AUNIP | FAM72B | GRPEL1 |
| FAM136A | C21orf45 | SUV39H2 |
| H2AFZ | GAR1 | GART |
| SET | KIF18B | LIN54 |
| EIF2S1 | SMC2 | NOLC1 |
| SDAD1 | NCAPD2 | NCL |
| RAD51AP1 | TIPIN | SKA3 |
| CPSF3 | POP1 | CDCA3 |
| RP4-785G19.2 | TCOF1 | LMNB1 |
| MIS18A | CENPH | KIF2C |
| GRPEL1 | HSPD1 | BIRC5 |
| SUV39H2 | H2AFZ | NCAPD2 |
| GART | CHAF1A | CKAP2 |
| LIN54 | NOL11 | NLN |
| NOLC1 | GMPS | GRSF1 |
| NCL | CDCA2 | ANLN |
| TMA16 | ARHGAP11B | STIP1 |
| SKA3 | CKAP5 | HMMR |
| CDCA3 | CDKN3 | CKAP5 |
| LMNB1 | BIRC5 | UCHL5 |
| KIF2C | RACGAP1 | POLR2D |
| BIRC5 | FAM72A | SSB |
| NCAPD2 | NUP205 | IMMT |
| CKAP2 | C15orf23 | PATL1 |
| NLN | RNASEH1 | CENPH |
| GRSF1 | CDC25C | PNO1 |
| TICRR | CKAP2 | PNPT1 |
| ANLN | SPC25 | USP14 |
| STIP1 | RRM1 | OLA1 |
| KNSTRN | GRPEL1 | RRM1 |
| HMMR | CENPI | SGOL2 |
| CKAP5 | ANLN | FAM83D |
| UCHL5 | PRKDC | CENPI |
| POLR2D | CEP55 | HJURP |
| SSB | MTIF2 | CPSF6 |
| IMMT | CEP135 | CENPN |
| PATL1 | EEF1E1 | SASS6 |
| CENPH | KIF15 | WDR12 |
| PNO1 | HJURP | HAUS6 |
| PNPT1 | PRPF40A | HEATR1 |
| USP14 | BUB3 | IARS |
| OLA1 | TOPBP1 | MTBP |
| RRM1 | TRIP13 | CEP55 |
| SGOL2 | SASS6 | POP1 |
| FAM83D | PATL1 | PGAM5 |
| CENPI | SDAD1 | BRCA1 |
| HJURP | IMMT | EIF2S2 |
| CPSF6 | WDR12 | HSPD1 |
| CENPN | RAD51AP1 | PPM1G |
| SASS6 | MTBP | TOPBP1 |
| WDR12 | SHCBP1 | MSH6 |
| HAUS6 | NOLC1 | ESPL1 |
| HEATR1 | UNG | C18orf54 |
| IARS | CDC45 | UBA6 |
| MTBP | DSCC1 | DENR |
| CEP55 | RBM28 | EXOSC2 |
| ZPR1 | CLPB | KIF20B |
| POP1 | CCNF | ZWILCH |
| PGAM5 | RUVBL1 | MRTO4 |
| BRCA1 | ATAD5 | ELAVL1 |
| EIF2S2 | VRK1 | CUL2 |
| HSPD1 | DHX15 | MRPL3 |
| PPM1G | NCAPD3 | PWP1 |
| TOPBP1 | IARS | DARS |
| MSH6 | PNPT1 | TRA2B |
| ESPL1 | BRCA1 | UNG |
| C18orf54 | MRTO4 | CDC25C |
| UBA6 | SSB | TMPO |
| DENR | RIOK1 | NUP37 |
| EXOSC2 | ENOPH1 | RFWD3 |
| KIF20B | GTSE1 | RRP1B |
| ZWILCH | SLBP | EIF4E |
| MRTO4 | UBA6 | GTSE1 |
| ELAVL1 | FAM72D | MTHFD1L |
| CUL2 | NDC80 |  |
| MRPL3 | BRCA2 |  |
| PWP1 | PPM1G |  |
| DARS | PGAM5 |  |
| TRA2B | PRPF4 |  |
| UNG | RAD54L |  |
| CDC25C | TACC3 |  |
| TMPO | LIN54 |  |
| NUP37 | SNRPD1 |  |
| ORC6 | CCNB2 |  |
| RFWD3 | EFTUD2 |  |
| RRP1B | TEX10 |  |
| EIF4E | UBE2K |  |
| GTSE1 | NUF2 |  |
| MTHFD1L | ORC6L |  |
|  | GRSF1 |  |
|  | MRPL3 |  |
|  | GTPBP4 |  |
|  | CDCA8 |  |
|  | UBE2T |  |
|  | EIF2C2 |  |
|  | EPR1 |  |
|  | KIF20B |  |
|  | ATIC |  |
|  | EXOC1 |  |
|  | KIFC1 |  |
|  | CENPA |  |
|  | C1orf163 |  |
|  | CENPN |  |
|  | UCHL5 |  |
|  | DARS |  |
|  | DIAPH3 |  |
|  | HAUS6 |  |
|  | DENR |  |
|  | LETM1 |  |
|  | RRP1B |  |
|  | RAD54B |  |
|  | NCAPG2 |  |
|  | HMMR |  |
|  | SGOL2 |  |
|  | TMPO |  |
|  | FBXO5 |  |
|  | PUS7 |  |
|  | EIF2S2 |  |
|  | MSH2 |  |
|  | MKI67IP |  |
|  | NOP58 |  |
|  | POLR1A |  |
|  | CCDC99 |  |
|  | ZNF259 |  |
|  | HEATR1 |  |
|  | NLN |  |
|  | TAF5 |  |
|  | ZWINT |  |
|  | TIMELESS |  |
|  | NFXL1 |  |
|  | MTHFD2 |  |
|  | C12orf48 |  |
|  | DCAF13 |  |
|  | XRCC2 |  |
|  | EXOSC9 |  |
|  | FBXO45 |  |
|  | FAM64A |  |
|  | SFRS1 |  |
|  | EXOSC2 |  |
|  | ELAVL1 |  |
|  | WDHD1 |  |
|  | NAA25 |  |
|  | MCM2 |  |
|  | RFC5 |  |
|  | DARS2 |  |
|  | KDM1A |  |
|  | OLA1 |  |
|  | MSH6 |  |
|  | ANAPC1 |  |
|  | SF3B2 |  |
|  | ECT2 |  |
|  | THOC4 |  |
|  | DHX33 |  |
|  | ZWILCH |  |
|  | ATAD2 |  |
|  | TCP1 |  |
|  | PAWR |  |
|  | EIF4E |  |
|  | CCT3 |  |
|  | TOMM70A |  |
|  | FANCD2 |  |
|  | DHX36 |  |
|  | MTHFD1 |  |
|  | POLA2 |  |
|  | CCT4 |  |
|  | OIP5 |  |
|  | TCERG1 |  |
|  | WDR3 |  |
|  | RPP40 |  |
|  | LARP1 |  |
|  | H2AFX |  |
|  | TDG |  |
|  | TMEM48 |  |
|  | RANBP1 |  |
|  | CAD |  |
|  | C18orf54 |  |
|  | POLR1B |  |
|  | MRPL19 |  |
|  | RFWD3 |  |
|  | PNO1 |  |
|  | POC1A |  |
|  | PDSS1 |  |
|  | HSPA4 |  |
|  | PBK |  |
|  | USP14 |  |
|  | PSMD11 |  |
|  | ABCF2 |  |
|  | L2HGDH |  |
|  | SET |  |
|  | CDK2 |  |
|  | MTA2 |  |
|  | RIF1 |  |
|  | DBF4B |  |
|  | RBL1 |  |
|  | EIF5B |  |
|  | C3orf26 |  |
|  | EME1 |  |
|  | BRIP1 |  |
|  | CCT5 |  |
|  | PSME3 |  |
|  | CCDC58 |  |
|  | DNAJC9 |  |
|  | TRAIP |  |
|  | CDT1 |  |
|  | MTHFD1L |  |
|  | NOL7 |  |
|  | POLR2D |  |
|  | WDR76 |  |
|  | LSM12 |  |
|  | YWHAG |  |
|  | FAM54A |  |
|  | RFC3 |  |
|  | PRPF19 |  |
|  | KIAA0406 |  |
|  | RNF26 |  |
|  | PTCD3 |  |
|  | ZC3H8 |  |
|  | SNRPA1 |  |
|  | HELLS |  |
|  | TUBG1 |  |
|  | KHDRBS1 |  |
|  | NUP37 |  |
|  | CLOCK |  |
|  | C2orf44 |  |
|  | C1orf112 |  |
|  | TYMS |  |
|  | DEK |  |
|  | POLQ |  |
|  | NUDCD1 |  |
|  | TEAD4 |  |
|  | CCDC43 |  |
|  | EIF2S1 |  |
|  | DHX37 |  |
|  | UBE2V2 |  |
|  | HNRNPL |  |
|  | PSMD2 |  |
|  | PAK1IP1 |  |
|  | DDB1 |  |
|  | EIF3J |  |
|  | DDX21 |  |
|  | CPSF3 |  |
|  | HMGB2 |  |
|  | GINS4 |  |
|  | PKMYT1 |  |
|  | CHAF1B |  |
|  | DLAT |  |
|  | ANP32B |  |
|  | SFXN1 |  |
|  | UCK2 |  |
|  | DONSON |  |
|  | AASDH |  |
|  | MYBL2 |  |
|  | ANAPC7 |  |
|  | WDR67 |  |
|  | BCCIP |  |
|  | CHORDC1 |  |
|  | C9orf140 |  |
|  | CENPK |  |
|  | CHCHD3 |  |
|  | SERBP1 |  |
|  | UTP18 |  |
|  | HLTF |  |
|  | C16orf59 |  |
|  | NCBP1 |  |
|  | DDX18 |  |
|  | POLR3G |  |
|  | TTL |  |
|  | E2F2 |  |
|  | TTF2 |  |
|  | HNRNPC |  |
|  | LRRC59 |  |
|  | C4orf43 |  |
|  | ESCO2 |  |
|  | TBRG4 |  |
|  | FANCG |  |
|  | BLM |  |
|  | MRPL1 |  |
|  | ACLY |  |
|  | PWP1 |  |
|  | DEPDC1B |  |
|  | SEH1L |  |
|  | NOP56 |  |
|  | STIL |  |
|  | MAPRE1 |  |
|  | PSMC3IP |  |
|  | CEBPZ |  |
|  | METAP1 |  |
|  | UTP6 |  |
|  | PPP1CC |  |
|  | TK1 |  |
|  | EIF4A1 |  |
|  | CUL2 |  |
|  | PPID |  |
|  | PRMT5 |  |
|  | MPHOSPH9 |  |
|  | RFC4 |  |
|  | UBE2S |  |
|  | SAP130 |  |
|  | SBNO1 |  |
|  | METTL8 |  |
|  | MCM3 |  |
|  | GABPB1 |  |
|  | GMNN |  |
|  | SRPK1 |  |
|  | PTBP1 |  |
|  | HAT1 |  |
|  | MRPL42 |  |
|  | NOP2 |  |
|  | ZW10 |  |
|  | HNRNPK |  |
|  | PSRC1 |  |
|  | WDR74 |  |
|  | C13orf34 |  |
|  | DNAJC7 |  |
|  | PRMT3 |  |
|  | CDC123 |  |
|  | GANAB |  |
|  | HNRNPA2B1 |  |
|  | SLC7A1 |  |
|  | C9orf100 |  |
|  | ZNF367 |  |
|  | HNRNPA3 |  |
|  | MLF1IP |  |
|  | RPL7L1 |  |
|  | EIF5AL1 |  |
|  | GINS1 |  |
|  | DNAJC11 |  |
|  | BMS1 |  |
|  | MPHOSPH10 |  |
|  | KIAA0101 |  |
|  | MRPL30 |  |
|  | DNAJA1 |  |
|  | PSMD14 |  |
|  | MAPKAPK5 |  |
|  | POLE2 |  |
|  | PDCD11 |  |
|  | RNF4 |  |
|  | AGPS |  |
|  | DCUN1D5 |  |
|  | POLD2 |  |
|  | DSP |  |
|  | KRR1 |  |
|  | G3BP1 |  |
|  | FUBP1 |  |
|  | EXOSC3 |  |
|  | TTC27 |  |
|  | USP46 |  |
|  | ZC3H15 |  |
|  | GCN1L1 |  |
|  | ZC3HAV1L |  |
|  | DDX55 |  |
|  | CEP78 |  |
|  | KHSRP |  |
|  | DNA2 |  |
|  | MDH2 |  |
|  | CDC27 |  |
|  | GINS2 |  |
|  | ASF1B |  |
|  | SCFD2 |  |
|  | PRIM1 |  |
|  | ESF1 |  |
|  | EIF4A3 |  |
|  | MDC1 |  |
|  | ABCF1 |  |
|  | TARDBP |  |
|  | C6orf167 |  |
|  | SMARCAD1 |  |
|  | TRA2B |  |
|  | XPOT |  |
|  | MTCH2 |  |
|  | DNAH14 |  |
|  | MTMR2 |  |
|  | TUBB |  |
|  | KIAA1429 |  |
|  | ZNF695 |  |
|  | ATAD3A |  |
|  | PIGW |  |
|  | C10orf2 |  |
|  | LTV1 |  |
|  | SLC25A32 |  |
|  | NEDD1 |  |
|  | MTPAP |  |
|  | TMEM194A |  |
|  | NAT10 |  |
|  | EPT1 |  |
|  | CENPL |  |
|  | SEPHS1 |  |
|  | SCLT1 |  |
|  | EZH2 |  |
|  | NKRF |  |
|  | RQCD1 |  |
|  | FXR1 |  |
|  | HSPA9 |  |
|  | C10orf18 |  |
|  | FADS1 |  |
|  | BBS7 |  |
|  | KNTC1 |  |
|  | PPIL5 |  |
|  | SCO1 |  |
|  | E2F8 |  |
|  | GPI |  |
|  | STRAP |  |
|  | AIMP1 |  |
|  | HDAC2 |  |
|  | GSTCD |  |
|  | MPP6 |  |
|  | WRNIP1 |  |
|  | GEMIN5 |  |
|  | NAA50 |  |
|  | PLAA |  |
|  | NAA35 |  |
|  | SLC5A6 |  |
|  | XPO1 |  |
|  | E2F3 |  |
|  | YWHAQ |  |
|  | MRPS9 |  |
|  | PTDSS1 |  |
|  | MCM7 |  |
|  | SFRS9 |  |
|  | SENP1 |  |
|  | AFG3L2 |  |
|  | SMN2 |  |
|  | DCAF16 |  |
|  | RAN |  |
|  | RNASEH2A |  |
|  | CDCA4 |  |
|  | AHCTF1 |  |
|  | CENPW |  |
|  | LIN9 |  |
|  | CS |  |
|  | N4BP2 |  |
|  | MRPS30 |  |
|  | VPS33A |  |
|  | YME1L1 |  |
|  | LOC221710 |  |
|  | TUBA1C |  |
|  | HNRNPA3P1 |  |
|  | C9orf40 |  |
|  | RRP15 |  |
|  | ADNP2 |  |
|  | MCM8 |  |
|  | RAD21 |  |
|  | FOXK2 |  |
|  | MRPS18C |  |
|  | HNRNPU |  |
|  | CPSF2 |  |
|  | GINS3 |  |
|  | FAM98B |  |
|  | OTUD6B |  |
|  | RAD18 |  |
|  | C2orf3 |  |
|  | TPRKB |  |
|  | C4orf46 |  |
|  | NOC2L |  |
|  | RPF2 |  |
|  | FKBP4 |  |
|  | LBR |  |
|  | CTPS |  |
|  | GTF3C2 |  |
|  | DSN1 |  |
|  | SAAL1 |  |
|  | ANKRD17 |  |
|  | SUZ12 |  |
|  | C16orf87 |  |
|  | PHB |  |
|  | NME1 |  |
|  | E2F1 |  |
|  | AASDHPPT |  |
|  | CCDC138 |  |
|  | HNRNPM |  |
|  | CEP152 |  |
|  | E2F7 |  |
|  | NARS |  |
|  | SIGMAR1 |  |
|  | CDC7 |  |
|  | MRPS5 |  |
|  | QTRTD1 |  |
|  | IPO4 |  |
|  | ZC3HC1 |  |
|  | PTPN11 |  |
|  | YEATS2 |  |
|  | TARS |  |
|  | RFC2 |  |
|  | NUP54 |  |
|  | KIAA0090 |  |
|  | CDK1 |  |
|  | CCDC85C |  |
|  | ZRANB3 |  |
|  | RCC2 |  |
|  | WDR4 |  |
|  | PSMD1 |  |
|  | NUP155 |  |
|  | NDUFS1 |  |
|  | TPI1 |  |
|  | YWHAZ |  |
|  | TIMM8A |  |
|  | XRCC5 |  |
|  | GEN1 |  |
|  | RBM17 |  |
|  | RAD23B |  |
|  | EIF2B1 |  |
|  | UTP15 |  |
|  | DHX9 |  |
|  | YES1 |  |
|  | RFC1 |  |
|  | HSP90AB1 |  |
|  | GAPDH |  |
|  | AP1AR |  |
|  | EIF3B |  |
|  | TUBA1B |  |
|  | PGM2 |  |
|  | BRIX1 |  |
|  | SMC6 |  |
|  | HMBS |  |
|  | LCLAT1 |  |
|  | NUP93 |  |
|  | RBM12 |  |
|  | GTF3C3 |  |
|  | PGAM1 |  |
|  | DNTTIP2 |  |
|  | POLD3 |  |
|  | YY1 |  |
|  | PARP1 |  |
|  | MYO19 |  |
|  | SUPT16H |  |
|  | CIRH1A |  |
|  | ARL5B |  |
|  | DFFA |  |
|  | MND1 |  |
|  | CACYBP |  |
|  | SMC3 |  |
|  | NUFIP1 |  |
|  | METTL2A |  |
|  | SYNCRIP |  |
|  | KPNA1 |  |
|  | TROAP |  |
|  | SNRNP27 |  |
|  | MRPL37 |  |
|  | FANCM |  |
|  | ACP1 |  |
|  | MARS2 |  |
|  | UBAP2 |  |
|  | EXOSC10 |  |
|  | DNAJC14 |  |
|  | WDR5 |  |
|  | SLC38A1 |  |
|  | GTF2H3 |  |
|  | CYCS |  |
|  | CBX1 |  |
|  | CCNJ |  |
|  | HSPA14 |  |
|  | DCAF17 |  |
|  | SMPD4 |  |
|  | HSPA4L |  |
|  | SART3 |  |
|  | UBXN2A |  |
|  | RSRC1 |  |
|  | CBS |  |
|  | PDXP |  |
|  | C18orf19 |  |
|  | FTSJ3 |  |
|  | USP1 |  |
|  | UMPS |  |
|  | SR140 |  |
|  | MINA |  |
|  | EIF1AD |  |
|  | NRAS |  |
|  | PFN2 |  |
|  | GFM1 |  |
|  | MRPS22 |  |
|  | EIF4G1 |  |
|  | IPO9 |  |
|  | PPIL1 |  |
|  | C9orf30 |  |
|  | CCDC21 |  |
|  | SRM |  |
|  | SFPQ |  |
|  | DHX57 |  |
|  | CEP76 |  |
|  | HDGF |  |
|  | OPA1 |  |
|  | PTTG1 |  |
|  | SNRNP200 |  |
|  | FAM111B |  |
|  | SMNDC1 |  |
|  | TBP |  |
|  | TAF1A |  |
|  | C7orf29 |  |
|  | ZNF286A |  |
|  | HMGA1 |  |
|  | ERAL1 |  |
|  | KIAA0020 |  |
|  | VCP |  |
|  | PES1 |  |
|  | EIF2AK2 |  |
|  | CHD7 |  |
|  | SAPS3 |  |
|  | MDH1 |  |
|  | LARS2 |  |
|  | NOC3L |  |
|  | SETD8 |  |
|  | WBP11 |  |
|  | FASTKD2 |  |
|  | RBM14 |  |
|  | DDX47 |  |
|  | SNRPE |  |
|  | AIMP2 |  |
|  | SUV39H1 |  |
|  | TACO1 |  |
|  | EIF5A |  |
|  | BRI3BP |  |
|  | STAMBP |  |
|  | UBE2R2 |  |
|  | LARP1B |  |
|  | RBMX |  |
|  | E2F6 |  |
|  | FASTKD1 |  |
|  | TRIM59 |  |
|  | RECQL4 |  |
|  | GARS |  |
|  | KPNA4 |  |
|  | HYLS1 |  |
|  | DDX1 |  |
|  | NLE1 |  |
|  | LOC441089 |  |
|  | BAT2 |  |
|  | EPRS |  |
|  | STOML2 |  |
|  | C2orf69 |  |
|  | POLR3A |  |
|  | CBX3 |  |
|  | SKP2 |  |
|  | USP10 |  |
|  | SAE1 |  |
|  | YKT6 |  |
|  | LRRC42 |  |
|  | IPO5 |  |
|  | USP5 |  |
|  | TAF2 |  |
|  | RG9MTD1 |  |
|  | PPP4R2 |  |
|  | HAUS3 |  |
|  | NUP160 |  |
|  | SLC4A1AP |  |
|  | SMU1 |  |
|  | CDC5L |  |
|  | PITPNB |  |
|  | WASF1 |  |
|  | METTL2B |  |
|  | ILF2 |  |
|  | PPIG |  |
|  | MRE11A |  |
|  | MAZ |  |
|  | C12orf43 |  |
|  | DNAJC2 |  |
|  | NAF1 |  |
|  | GNL3 |  |
|  | FANCC |  |
|  | SMS |  |
|  | INTS2 |  |
|  | CDC23 |  |
|  | ZBTB2 |  |
|  | SNRPF |  |
|  | C12orf24 |  |
|  | THOP1 |  |
|  | SNF8 |  |
|  | MTL5 |  |
|  | UGGT1 |  |
|  | MRPL47 |  |
|  | PSMD7 |  |
|  | CDK8 |  |
|  | METTL5 |  |
|  | GUF1 |  |
|  | TMEM206 |  |
|  | RPAP3 |  |
|  | C16orf88 |  |
|  | NOL9 |  |
|  | DHFR |  |
|  | HSPE1 |  |
|  | NKIRAS2 |  |
|  | PAXIP1 |  |
|  | PSMB2 |  |
|  | GPN3 |  |
|  | RAD51C |  |
|  | SRPK2 |  |
|  | RNF214 |  |
|  | SENP3 |  |
|  | PRTFDC1 |  |
|  | QSER1 |  |
|  | VTA1 |  |
|  | DAZAP1 |  |
|  | APTX |  |
|  | PCNA |  |
|  | CISD2 |  |
|  | VDAC1 |  |
|  | ILF3 |  |
|  | PDCD2 |  |
|  | SEC23A |  |
|  | ZFP91 |  |
|  | PHF6 |  |
|  | DNMT1 |  |
|  | HOMER1 |  |
|  | ZBTB9 |  |
|  | JARID2 |  |
|  | FGFR1OP |  |
|  | SSX2IP |  |
|  | PNMA1 |  |
|  | PDCL3 |  |
|  | LMAN1 |  |
|  | NUP85 |  |
|  | GRPEL2 |  |
|  | FAM199X |  |
|  | NONO |  |
|  | TOMM40 |  |
|  | SMYD5 |  |
|  | C17orf71 |  |
|  | LDHA |  |
|  | CNOT10 |  |
|  | DPH2 |  |
|  | NCBP2 |  |
|  | BAG2 |  |
|  | LRRC40 |  |
|  | RGP1 |  |
|  | RARS |  |
|  | LOC727896 |  |
|  | MRFAP1 |  |
|  | YRDC |  |
|  | KIF5B |  |
|  | ATP5B |  |
|  | PRDM4 |  |
|  | RRP9 |  |
|  | GAS2L3 |  |
|  | PSME4 |  |
|  | NIP7 |  |
|  | HBS1L |  |
|  | RPUSD4 |  |
|  | TTPAL |  |
|  | RBM27 |  |
|  | NUDT15 |  |
|  | UTP11L |  |
|  | C2orf49 |  |
|  | CCAR1 |  |
|  | HCFC1 |  |
|  | C17orf53 |  |
|  | TAF9 |  |
|  | SNW1 |  |
|  | EBNA1BP2 |  |
|  | PSIMCT-1 |  |
|  | CSNK2A1P |  |
|  | SMARCC1 |  |
|  | TMEM209 |  |
|  | TNPO3 |  |
|  | RRP12 |  |
|  | UTP20 |  |
|  | FANCA |  |
|  | ENO1 |  |
|  | YARS |  |
|  | EIF4H |  |
|  | C11orf84 |  |
|  | ISG20L2 |  |
|  | UTP14A |  |
|  | C1orf109 |  |
|  | NSUN2 |  |
|  | C19orf48 |  |
|  | NUP43 |  |
|  | HAUS2 |  |
|  | LONP1 |  |
|  | BAZ1B |  |
|  | GOT2 |  |
|  | VRK2 |  |
|  | RDX |  |
|  | STMN1 |  |
|  | RPE |  |
|  | TLK2 |  |
|  | C5orf34 |  |
|  | C6orf153 |  |
|  | CDC73 |  |
|  | KIF24 |  |
|  | DHCR7 |  |
|  | C12orf32 |  |
|  | DTYMK |  |
|  | TRIM28 |  |
|  | SF3B3 |  |
|  | GPATCH4 |  |
|  | C17orf96 |  |
|  | GNPNAT1 |  |
|  | CLNS1A |  |
|  | LIG3 |  |
|  | LUC7L2 |  |
|  | SNRPC |  |
|  | ANP32A |  |
|  | TNPO1 |  |
|  | TUBGCP4 |  |
|  | STT3A |  |
|  | RNF34 |  |
|  | AATF |  |
|  | UBE2C |  |
|  | UBE2G1 |  |
|  | TET3 |  |
|  | ALKBH2 |  |
|  | TMEM33 |  |
|  | BARD1 |  |
|  | TMEM185B |  |
|  | NUP35 |  |
|  | SPC24 |  |
|  | SUPV3L1 |  |
|  | ACTL6A |  |
|  | ZNF639 |  |
|  | SMC4 |  |
|  | CWC27 |  |
|  | HNRNPH3 |  |
|  | SLC7A5 |  |
|  | PAFAH1B2 |  |
|  | SPATA5 |  |
|  | INTS7 |  |
|  | C12orf11 |  |
|  | NFKBIL2 |  |
|  | ARMC1 |  |
|  | MRPL12 |  |
|  | LSG1 |  |
|  | PHF19 |  |
|  | FIGNL1 |  |
|  | GNL2 |  |
|  | C10orf12 |  |
|  | REXO4 |  |
|  | ARCN1 |  |
|  | TSSC1 |  |
|  | CPSF7 |  |
|  | TMTC3 |  |
|  | SPATS2 |  |
|  | KARS |  |
|  | GLRX5 |  |
|  | HTRA2 |  |
|  | PGAM4 |  |
|  | SF3A3 |  |
|  | RPP30 |  |
|  | RAB6A |  |
|  | TAF11 |  |
|  | DCUN1D4 |  |
|  | PSMB5 |  |
|  | CDV3 |  |
|  | RPS6KC1 |  |
|  | TRAP1 |  |
|  | BOP1 |  |
|  | ARL6IP6 |  |
|  | ASAP1 |  |
|  | C14orf106 |  |
|  | ACTR6 |  |
|  | POFUT1 |  |
|  | PWP2 |  |
|  | SUV420H1 |  |
|  | MFN1 |  |
|  | CSNK2A1 |  |
|  | FUBP3 |  |
|  | DDX27 |  |
|  | POLA1 |  |
|  | MED17 |  |
|  | TBC1D1 |  |
|  | NDUFAF4 |  |
|  | DDX39 |  |
|  | AURKAPS1 |  |
|  | FIGN |  |
|  | BZW2 |  |
|  | BUD13 |  |
|  | SF3B14 |  |
|  | NUDT21 |  |
|  | PRPS1 |  |
|  | WHSC2 |  |
|  | HSP90AA1 |  |
|  | SENP2 |  |
|  | CHIC2 |  |
|  | ORC2L |  |
|  | CHRAC1 |  |
|  | SNRNP48 |  |
|  | INTS8 |  |
|  | VBP1 |  |
|  | CCDC15 |  |
|  | TMX2 |  |
|  | TIMM17A |  |
|  | PCBP1 |  |
|  | C10orf119 |  |
|  | ALG8 |  |
|  | C14orf33 |  |
|  | ZNF207 |  |
|  | HEATR2 |  |
|  | NETO2 |  |
|  | CLP1 |  |
|  | RTKN |  |
|  | SNRNP40 |  |
|  | ALS2CR4 |  |
|  | IPPK |  |
|  | TERF1 |  |
|  | CCDC137 |  |
|  | SMC1A |  |
|  | SAMD1 |  |
|  | AQR |  |
|  | SFRS2 |  |
|  | UTP3 |  |
|  | TMEM201 |  |
|  | PAK2 |  |
|  | RBM15 |  |
|  | GPR19 |  |
|  | EIF2C3 |  |
|  | CD3EAP |  |
|  | AGFG1 |  |
|  | CKS1B |  |
|  | TADA2A |  |
|  | MEST |  |
|  | CCDC34 |  |
|  | ZNF623 |  |
|  | EED |  |
|  | ATP6V1C1 |  |
|  | UPF3B |  |
|  | MYBBP1A |  |
|  | RECQL |  |
|  | CSTF3 |  |
|  | RBM19 |  |
|  | SMARCA5 |  |
|  | GNL3L |  |
|  | UHRF1BP1 |  |
|  | AHSA1 |  |
|  | CBX5 |  |
|  | RANGAP1 |  |
|  | POLR1E |  |
|  | UBXN7 |  |
|  | MRPL11 |  |
|  | PSMG1 |  |
|  | LARP4 |  |
|  | TAF1B |  |
|  | PPP2R1B |  |
|  | C17orf75 |  |
|  | MAP6D1 |  |
|  | NUP98 |  |
|  | RMI1 |  |
|  | NUP188 |  |
|  | NMD3 |  |
|  | C15orf41 |  |
|  | SRRD |  |
|  | LOC100128191 |  |
|  | POLE |  |
|  | TOMM5 |  |
|  | ECE2 |  |
|  | WDR36 |  |
|  | MAPK6 |  |
|  | PSAT1 |  |
|  | HNRNPAB |  |
|  | NASP |  |
|  | EIF3A |  |
|  | C2orf43 |  |
|  | EXOC5 |  |
|  | UBE4A |  |
|  | OXSR1 |  |
|  | VDAC2 |  |
|  | TSN |  |
|  | TWISTNB |  |
|  | C18orf55 |  |
|  | FARSA |  |
|  | METAP2 |  |
|  | PDHX |  |
|  | MRPL51 |  |
|  | NRD1 |  |
|  | ZNF473 |  |
|  | LRRC58 |  |
|  | DCAF7 |  |
|  | UBQLN1 |  |
|  | LOC401010 |  |
|  | SSBP1 |  |
|  | SKA2 |  |
|  | CEP250 |  |
|  | IGF2BP3 |  |
|  | FAM189B |  |
|  | IQGAP3 |  |
|  | TFAM |  |
|  | NAE1 |  |
|  | RRP1 |  |
|  | KCMF1 |  |
|  | C20orf20 |  |
|  | ADAM17 |  |
|  | HNRNPA1L2 |  |
|  | ATP13A3 |  |
|  | PCGF6 |  |
|  | C6orf150 |  |
|  | MRPL16 |  |
|  | CENPQ |  |
|  | NUDT1 |  |
|  | USP42 |  |
|  | ZNF146 |  |
|  | C1QBP |  |
|  | RUVBL2 |  |
|  | C11orf57 |  |
|  | CWC22 |  |
|  | KLC2 |  |
|  | SFXN4 |  |
|  | MYL6B |  |
|  | SENP5 |  |
|  | HUWE1 |  |
|  | GTF2F2 |  |
|  | SPIN4 |  |
|  | COPS8 |  |
|  | ANKIB1 |  |
|  | MAP4K4 |  |
|  | WAC |  |
|  | EIF3M |  |
|  | HNRNPUL2 |  |
|  | DNMT3A |  |
|  | PRMT1 |  |
|  | WDR77 |  |
|  | LIG1 |  |
|  | NBN |  |
|  | COIL |  |
|  | SOX12 |  |
|  | MEN1 |  |
|  | C4orf21 |  |
|  | PDS5A |  |
|  | TMEM199 |  |
|  | PARD3 |  |
|  | EXOC2 |  |
|  | MRPL15 |  |
|  | MAGOHB |  |
|  | ZNF384 |  |
|  | MYC |  |
|  | QSOX2 |  |
|  | PUS3 |  |
|  | ZNF544 |  |
|  | LRP12 |  |
|  | RDM1 |  |
|  | CTNNBL1 |  |
|  | GNAI3 |  |
|  | SFRS13A |  |
|  | DCLRE1B |  |
|  | USP37 |  |
|  | MARS |  |
|  | PPP2R5E |  |
|  | C12orf73 |  |
|  | INTS6 |  |
|  | GSS |  |
|  | FBXO30 |  |
|  | MTERFD1 |  |
|  | PSMC5 |  |
|  | MORF4L2 |  |
|  | OBFC2B |  |
|  | SMCR7L |  |
|  | BRAP |  |
|  | UBE2N |  |
|  | PMS1 |  |
|  | DDX56 |  |
|  | PELP1 |  |
|  | AHCY |  |
|  | SLC25A13 |  |
|  | NOP16 |  |
|  | MTA3 |  |
|  | SFRS7 |  |
|  | ZNF326 |  |
|  | G2E3 |  |
|  | SCFD1 |  |
|  | POLDIP2 |  |
|  | SSR1 |  |
|  | SP3 |  |
|  | UBTF |  |
|  | RSL1D1 |  |
|  | CCDC59 |  |
|  | YWHAE |  |
|  | RBM12B |  |
|  | CSTF2 |  |
|  | GPSM2 |  |
|  | FAM98A |  |
|  | MAK16 |  |
|  | TRMT6 |  |
|  | PARG |  |
|  | PSMB7 |  |
|  | UBE2O |  |
|  | MTA1 |  |
|  | RPAP2 |  |
|  | NEIL3 |  |
|  | PTGES3 |  |
|  | PUS1 |  |
|  | VDAC3 |  |
|  | C13orf37 |  |
|  | PREB |  |
|  | PPME1 |  |
|  | HPRT1 |  |
|  | CSTF1 |  |
|  | LOC550112 |  |
|  | FAM104A |  |
|  | EI24 |  |
|  | ZMYND19 |  |
|  | LEO1 |  |
|  | CFL1 |  |
|  | UBE2MP1 |  |
|  | LZIC |  |
|  | GMCL1 |  |
|  | ACAT2 |  |
|  | MTRF1L |  |
|  | DDX54 |  |
|  | ADSL |  |
|  | KIAA0947 |  |
|  | FAM161A |  |
|  | HMGXB4 |  |
|  | FAM20B |  |
|  | C1orf96 |  |
|  | KIAA1586 |  |
|  | UPF2 |  |
|  | SHMT2 |  |
|  | GTF2E1 |  |
|  | MRPL35 |  |
|  | PFKM |  |
|  | NUS1 |  |
|  | LSM5 |  |
|  | CCDC112 |  |
|  | ATP2B1 |  |
|  | POLE3 |  |
|  | HYOU1 |  |
|  | TGIF2 |  |
|  | C1orf103 |  |
|  | UCHL3 |  |
|  | PPP1R14B |  |
|  | WDR53 |  |
|  | ACTG1 |  |
|  | FAF1 |  |
|  | XRCC3 |  |
|  | RDBP |  |
|  | RNASEN |  |
|  | CBL |  |
|  | PA2G4P4 |  |
|  | LUZP6 |  |
|  | NCOA6 |  |
|  | AVL9 |  |
|  | DYNC1LI1 |  |
|  | FKBP3 |  |
|  | TGS1 |  |
|  | RAE1 |  |
|  | CTDSPL2 |  |
|  | LARS |  |
|  | HIRA |  |
|  | PARP2 |  |
|  | EHBP1 |  |
|  | MFSD2B |  |
|  | IPO7 |  |
|  | DBN1 |  |
|  | PPP2R5D |  |
|  | TIMM44 |  |
|  | CEP57 |  |
|  | ACOT7 |  |
|  | KIF2A |  |
|  | CSNK2B |  |
|  | CCNK |  |
|  | COPB2 |  |
|  | NIF3L1 |  |
|  | SNAPC3 |  |
|  | COPS7B |  |
|  | EIF2A |  |
|  | SMARCAL1 |  |
|  | CBLL1 |  |
|  | TAF1D |  |
|  | DNAJB11 |  |
|  | CFL2 |  |
|  | TFDP2 |  |
|  | SPATA5L1 |  |
|  | YARS2 |  |
|  | SND1 |  |
|  | UBR5 |  |
|  | UBA2 |  |
|  | ZFR |  |
|  | NUP62 |  |
|  | IMP4 |  |
|  | CSNK1G1 |  |
|  | SLC25A10 |  |
|  | AGBL5 |  |
|  | SLC16A1 |  |
|  | FAM168B |  |
|  | DDX46 |  |
|  | GTPBP10 |  |
|  | SLC25A33 |  |
|  | PDK1 |  |
|  | SLC25A3 |  |
|  | COX10 |  |
|  | CEP72 |  |
|  | GNB1 |  |
|  | SYCE2 |  |
|  | TTC4 |  |
|  | USP39 |  |
|  | MAEA |  |
|  | ABL2 |  |
|  | C9orf41 |  |
|  | ISY1 |  |
|  | MED1 |  |
|  | STC2 |  |
|  | COX4NB |  |
|  | KPNA3 |  |
|  | DYNC1H1 |  |
|  | C17orf80 |  |
|  | HNRPLL |  |
|  | MYBL1 |  |
|  | ACTR3B |  |
|  | LOC643387 |  |
|  | ERI1 |  |
|  | MTOR |  |
|  | MMACHC |  |
|  | RALA |  |
|  | RPA1 |  |
|  | PPIF |  |
|  | SLC30A6 |  |
|  | POLR2H |  |
|  | ALG3 |  |
|  | STRN |  |
|  | NDRG3 |  |
|  | C1orf107 |  |
|  | CEP63 |  |
|  | CCNE2 |  |
|  | PTRH2 |  |
|  | MTX2 |  |
|  | ARMC8 |  |
|  | STYX |  |
|  | NUP50 |  |
|  | ATL3 |  |
|  | DIP2B |  |
|  | CRKL |  |
|  | TOP3A |  |
|  | C14orf145 |  |
|  | KIAA0586 |  |
|  | ANKRD52 |  |
|  | GRWD1 |  |
|  | C7orf49 |  |
|  | SRRT |  |
|  | PFAS |  |
|  | U2AF2 |  |
|  | ATF2 |  |
|  | FAM175B |  |
|  | SART1 |  |
|  | SMG7 |  |
|  | NOM1 |  |
|  | EIF2B3 |  |
|  | TFG |  |
|  | DPP3 |  |
|  | NOL6 |  |
|  | FYTTD1 |  |
|  | INTS4 |  |
|  | CHAC2 |  |
|  | RPS6KB1 |  |
|  | TOMM34 |  |
|  | DUS4L |  |
|  | TLK1 |  |
|  | PHAX |  |
|  | XPO6 |  |
|  | PIGU |  |
|  | SNRPA |  |
|  | DRG1 |  |
|  | PPAN |  |
|  | PSMA2 |  |
|  | HNRNPF |  |
|  | ZFP64 |  |
|  | AMMECR1 |  |
|  | TRUB1 |  |
|  | UBE2L3 |  |
|  | SLC2A1 |  |
|  | RBM45 |  |
|  | C1orf124 |  |
|  | RALBP1 |  |
|  | KLHL23 |  |
|  | RBBP4 |  |
|  | MED6 |  |
|  | HAUS8 |  |
|  | PLEKHA8 |  |
|  | DLD |  |
|  | UBE3C |  |
|  | ATP2A2 |  |
|  | IRAK1 |  |
|  | MCART1 |  |
|  | NIPA2 |  |
|  | KIF1B |  |
|  | CKS2 |  |
|  | FH |  |
|  | CLASP1 |  |
|  | STAU1 |  |
|  | AZIN1 |  |
|  | ADNP |  |
|  | PSPC1 |  |
|  | ACTR3 |  |
|  | ZCCHC4 |  |
|  | DDX52 |  |
|  | SMG5 |  |
|  | ZNF280B |  |
|  | IWS1 |  |
|  | CNO |  |
|  | PPP1R12A |  |
|  | PSIP1 |  |
|  | SPAST |  |
|  | NFS1 |  |
|  | RIC8B |  |
|  | RG9MTD2 |  |
|  | UBE2E3 |  |
|  | DVL2 |  |
|  | ERCC3 |  |
|  | MGA |  |
|  | RWDD4A |  |
|  | ARL6IP1 |  |
|  | SIP1 |  |
|  | STAG1 |  |
|  | ESRP1 |  |
|  | NMT1 |  |
|  | RAB10 |  |
|  | MCM3APAS |  |
|  | GMEB1 |  |
|  | RCCD1 |  |
|  | GPN1 |  |
|  | RELA |  |
|  | PTCD1 |  |
|  | FBXL18 |  |
|  | FOSL1 |  |
|  | BYSL |  |
|  | CELF1 |  |
|  | MRPS10 |  |
|  | GLMN |  |
|  | C8orf33 |  |
|  | GATC |  |
|  | UCHL1 |  |
|  | SFRS3 |  |
|  | SUGT1 |  |
|  | CAPRIN1 |  |
|  | HSF1 |  |
|  | LAS1L |  |
|  | PSMC3 |  |
|  | CHD8 |  |
|  | HS2ST1 |  |
|  | CENPJ |  |
|  | KIAA0368 |  |
|  | CDK16 |  |
|  | ATXN7L3 |  |
|  | ZSCAN20 |  |
|  | CCDC51 |  |
|  | IFRD1 |  |
|  | NSDHL |  |
|  | PYGL |  |
|  | MEMO1 |  |
|  | PLOD2 |  |
|  | FAM91A1 |  |
|  | MAPK8 |  |
|  | RTCD1 |  |
|  | GATSL1 |  |
|  | SLC35F2 |  |
|  | MMADHC |  |
|  | CHUK |  |
|  | NUDT5 |  |
|  | ZNF770 |  |
|  | SDCCAG3 |  |
|  | DNAJA3 |  |
|  | TMEM135 |  |
|  | UBE2V1 |  |
|  | POLD1 |  |
|  | RAP1GDS1 |  |
|  | TRMT61B |  |
|  | RIPK2 |  |
|  | MPP2 |  |
|  | ZBTB39 |  |
|  | SF1 |  |
|  | HTT |  |
|  | DCUN1D1 |  |
|  | GPD2 |  |
|  | ORC5L |  |
|  | PUF60 |  |
|  | DNMT3B |  |
|  | TRIP12 |  |
|  | PPIAL4C |  |
|  | NFRKB |  |
|  | APEX1 |  |
|  | COPG2 |  |
|  | VARS |  |
|  | C5orf22 |  |
|  | C10orf88 |  |
|  | JMJD6 |  |
|  | C20orf27 |  |
|  | MCM5 |  |
|  | CBWD1 |  |
|  | ATPBD4 |  |
|  | YBX2 |  |
|  | RTN3 |  |
|  | PPP1R8 |  |
|  | RNF2 |  |
|  | SRP68 |  |
|  | C17orf58 |  |
|  | POM121 |  |
|  | MRPL10 |  |
|  | KIAA1715 |  |
|  | HSPA8 |  |
|  | CASP8AP2 |  |
|  | PPP3R1 |  |
|  | DHX8 |  |
|  | REPS1 |  |
|  | C2orf47 |  |
|  | ZUFSP |  |
|  | TRMT5 |  |
|  | CDYL |  |
|  | ZC3HAV1 |  |
|  | SPIN1 |  |
|  | C3orf37 |  |
|  | MRPL44 |  |
|  | CEP97 |  |
|  | CHCHD8 |  |
|  | ZCCHC8 |  |
|  | DDX23 |  |
|  | UBIAD1 |  |
|  | PGM3 |  |
|  | SLC38A7 |  |
|  | PFKP |  |
|  | THRAP3 |  |
|  | ZNF239 |  |
|  | USP32 |  |
|  | GMFB |  |
|  | IMPAD1 |  |
|  | STRN3 |  |
|  | ATP5G3 |  |
|  | SGTA |  |
|  | HOOK1 |  |
|  | C20orf72 |  |
|  | DOCK7 |  |
|  | B3GNT5 |  |
|  | PGBD1 |  |
|  | PRKAA2 |  |
|  | PALB2 |  |
|  | PAPOLA |  |
|  | HSP90AB2P |  |
|  | KIAA0114 |  |
|  | ESCO1 |  |
|  | MTAP |  |
|  | UBFD1 |  |
|  | DNM1L |  |
|  | C16orf75 |  |
|  | PVR |  |
|  | MTFR1 |  |
|  | PM20D2 |  |
|  | SAP30 |  |
|  | PHF10 |  |
|  | DBR1 |  |
|  | COQ2 |  |
|  | CIAO1 |  |
|  | FUS |  |
|  | SNAPC1 |  |
|  | RPAP1 |  |
|  | CBX2 |  |
|  | RBBP7 |  |
|  | RAD50 |  |
|  | RSF1 |  |
|  | TPR |  |
|  | KTN1 |  |
|  | SAFB |  |
|  | SMARCD1 |  |
|  | PTMA |  |
|  | PIK3R4 |  |
|  | SCYL2 |  |
|  | 6-Mar |  |
|  | VANGL1 |  |
|  | PIGX |  |
|  | GTF2H2 |  |
|  | DHX16 |  |
|  | FBXO22OS |  |
|  | GPRIN1 |  |
|  | DYNLL1 |  |
|  | TAF3 |  |
|  | ASXL1 |  |
|  | ZNF620 |  |
|  | USO1 |  |
|  | PFKFB4 |  |
|  | BEND3 |  |
|  | DCK |  |
|  | ZYG11A |  |
|  | SMARCB1 |  |
|  | COPS5 |  |
|  | PARL |  |
|  | COPG |  |
|  | SNRPD3 |  |
|  | TCF19 |  |
|  | TBC1D16 |  |
|  | C14orf104 |  |
|  | CHEK2 |  |
|  | KBTBD2 |  |
|  | COMMD2 |  |
|  | RRS1 |  |
|  | PPT2 |  |
|  | SUMO2 |  |
|  | FBL |  |
|  | TRMT12 |  |
|  | CASP2 |  |
|  | POLR3D |  |
|  | ZAK |  |
|  | C6orf182 |  |
|  | NAA40 |  |
|  | PPRC1 |  |
|  | H2AFV |  |
|  | KDM1B |  |
|  | AGPAT5 |  |
|  | RNF8 |  |
|  | PANX1 |  |
|  | KDELC2 |  |
|  | KIAA1958 |  |
|  | REST |  |
|  | DHX30 |  |
|  | PHKA1 |  |
|  | DVL3 |  |
|  | ZKSCAN5 |  |
|  | KIAA1549 |  |
|  | ABCF3 |  |
|  | DNAJC13 |  |
|  | PCCB |  |
|  | KIN |  |
|  | TMEM189 |  |
|  | TNPO2 |  |
|  | DDX20 |  |
|  | ENAH |  |
|  | C14orf80 |  |
|  | SYNJ2 |  |
|  | U2AF1 |  |
|  | AARSD1 |  |
|  | TIMM10 |  |
|  | GTF3C4 |  |
|  | TMEM177 |  |
|  | PHF20L1 |  |
|  | GTF2IRD1 |  |
|  | MIB1 |  |
|  | AK3L1 |  |
|  | NUP88 |  |
|  | DPF2 |  |
|  | HARS |  |
|  | SRP54 |  |
|  | DCAF10 |  |
|  | ERC1 |  |
|  | DAP3 |  |
|  | LOC144438 |  |
|  | MAP1D |  |
|  | VAPB |  |
|  | COQ3 |  |
|  | ARHGAP19 |  |
|  | DYNC1I2 |  |
|  | TMEM194B |  |
|  | CALU |  |
|  | MAP7D3 |  |
|  | POLR3B |  |
|  | ABCB7 |  |
|  | PLEKHA9 |  |
|  | GPATCH2 |  |
|  | BRD4 |  |
|  | YBX1 |  |
|  | PIF1 |  |
|  | OGFOD1 |  |
|  | KLHL5 |  |
|  | BOLA3 |  |
|  | NRM |  |
|  | ZNF670 |  |
|  | GOSR1 |  |
|  | PUS7L |  |
|  | OSGIN2 |  |
|  | NCKAP1 |  |
|  | WDR46 |  |
|  | CUL5 |  |
|  | CHD4 |  |
|  | GPR125 |  |
|  | CENPM |  |
|  | MRPL45 |  |
|  | ELOVL5 |  |
|  | KIAA0317 |  |
|  | ZNF518B |  |
|  | SFRS13B |  |
|  | ZDBF2 |  |
|  | G3BP2 |  |
|  | NHLRC2 |  |
|  | CPSF6 |  |
|  | MTDH |  |
|  | EDC3 |  |
|  | RCOR2 |  |
|  | COASY |  |
|  | PSMA4 |  |
|  | UHRF1BP1L |  |
|  | SOCS7 |  |
|  | NPEPPS |  |
|  | RCE1 |  |
|  | LDLRAD3 |  |
|  | C19orf57 |  |
|  | ERCC8 |  |
|  | SKIV2L2 |  |
|  | TXNRD1 |  |
|  | ITCH |  |
|  | XPO7 |  |
|  | MOCS3 |  |
|  | COG5 |  |
|  | ATRIP |  |
|  | FAM86C |  |
|  | CTSL2 |  |
|  | RCC1 |  |
|  | BBX |  |
|  | BAT2L2 |  |
|  | MRPS16 |  |
|  | HTATSF1 |  |
|  | DCPS |  |
|  | TMEM184C |  |
|  | HNRNPA1 |  |
|  | C10orf46 |  |
|  | PDCD2L |  |
|  | TP53BP1 |  |
|  | DIS3 |  |
|  | CARM1 |  |
|  | B3GALNT2 |  |
|  | EIF4G3 |  |
|  | TIMM22 |  |
|  | MINPP1 |  |
|  | PPP2CA |  |
|  | NT5C3 |  |
|  | CIAPIN1 |  |
|  | POM121C |  |
|  | ZNF697 |  |
|  | MOBKL1B |  |
|  | IREB2 |  |
|  | SLC36A4 |  |
|  | UTP23 |  |
|  | NUP210 |  |
|  | PL-5283 |  |
|  | SUDS3 |  |
|  | DHX35 |  |
|  | TH1L |  |
|  | FXN |  |
|  | BRCC3 |  |
|  | UBE2Z |  |
|  | USP28 |  |
|  | STAM |  |
|  | NEU3 |  |
|  | ANKLE2 |  |
|  | NUP133 |  |
|  | SLMO2 |  |
|  | GEMIN6 |  |
|  | DPM1 |  |
|  | WDR92 |  |
|  | GJC1 |  |
|  | RPTOR |  |
|  | PSMB6 |  |
|  | FADS2 |  |
|  | PYCR1 |  |
|  | OSGEPL1 |  |
|  | SLC20A1 |  |
|  | RNF219 |  |
|  | DPAGT1 |  |
|  | CHCHD4 |  |
|  | PCNXL3 |  |
|  | OTUD4 |  |
|  | TMEM69 |  |
|  | AMMECR1L |  |
|  | PEX5 |  |
|  | WDR45L |  |
|  | RBM15B |  |
|  | MLLT11 |  |
|  | TFRC |  |
|  | ALDOA |  |
|  | ETF1 |  |
|  | PHLPP1 |  |
|  | RPIA |  |
|  | LOC729020 |  |
|  | TCF3 |  |
|  | RBBP5 |  |
|  | CDCP1 |  |
|  | ZZZ3 |  |
|  | KDM3A |  |
|  | LMBR1 |  |
|  | ZNF232 |  |
|  | JRK |  |
|  | VPRBP |  |
|  | GSK3B |  |
|  | MAPK1IP1L |  |
|  | BLMH |  |
|  | APOOL |  |
|  | GRK4 |  |
|  | MAFG |  |
|  | ZNF496 |  |
|  | HADHA |  |
|  | NARS2 |  |
|  | NIPSNAP1 |  |
|  | TRIM37 |  |
|  | HPS3 |  |
|  | E2F5 |  |
|  | MSI2 |  |
|  | SRFBP1 |  |
|  | KPNA6 |  |
|  | PNPO |  |
|  | RNGTT |  |
|  | ANP32E |  |
|  | C1orf131 |  |
|  | RALGAPB |  |
|  | MEX3A |  |
|  | DCLRE1A |  |
|  | FIBP |  |
|  | FLVCR1 |  |
|  | WDR1 |  |
|  | C12orf29 |  |
|  | RNF115 |  |
|  | LAMC1 |  |
|  | GIT1 |  |
|  | C22orf30 |  |
|  | MAP3K2 |  |
|  | UBAP2L |  |
|  | EIF2AK1 |  |
|  | PRPF38A |  |
|  | IARS2 |  |
|  | FANCE |  |
|  | ZBTB12 |  |
|  | LARP7 |  |
|  | PHF20 |  |
|  | KIAA0100 |  |
|  | CUL4B |  |
|  | AKIRIN1 |  |
|  | ITSN1 |  |
|  | SOCS4 |  |
|  | RDH11 |  |
|  | BANF1 |  |
|  | SLC3A2 |  |
|  | MBTPS2 |  |
|  | LOC150786 |  |
|  | KIAA0895 |  |
|  | FAM168A |  |
|  | NDUFA12 |  |
|  | VMA21 |  |
|  | STARD7 |  |
|  | SDHA |  |
|  | IPO8 |  |
|  | EHMT2 |  |
|  | FBXL19 |  |
|  | FAM119A |  |
|  | CEBPG |  |
|  | TMX1 |  |
|  | PTBP2 |  |
|  | CCDC88A |  |
|  | RAB2A |  |
|  | KDM5A |  |
|  | ERO1L |  |
|  | NCOA3 |  |
|  | SMCHD1 |  |
|  | INTS12 |  |
|  | GNA13 |  |
|  | C7orf11 |  |
|  | MAPK1 |  |
|  | PCBP2 |  |
|  | ASCC3 |  |
|  | UBQLN4 |  |
|  | ENY2 |  |
|  | ELOVL6 |  |
|  | HSP90B1 |  |
|  | FTSJ1 |  |
|  | ZNF525 |  |
|  | ATP5C1 |  |
|  | RPF1 |  |
|  | ING1 |  |
|  | LOC93622 |  |
|  | KIAA1609 |  |
|  | CNOT1 |  |
|  | BOD1L |  |
|  | GTF2E2 |  |
|  | ATXN2L |  |
|  | MNAT1 |  |
|  | TSGA14 |  |
|  | GLRX2 |  |
|  | ATXN3 |  |
|  | TRIAP1 |  |
|  | GDI2 |  |
|  | MLF2 |  |
|  | MARK4 |  |
|  | TIGD5 |  |
|  | SMARCE1 |  |
|  | ZNF718 |  |
|  | ZNF598 |  |
|  | C14orf21 |  |
|  | HMGCR |  |
|  | RC3H2 |  |
|  | TIPRL |  |
|  | SNHG1 |  |
|  | PCMT1 |  |
|  | UBE2M |  |
|  | LOC341056 |  |
|  | NANP |  |
|  | PIAS2 |  |
|  | TFB2M |  |
|  | MRPL39 |  |
|  | FAM114A1 |  |
|  | C2CD3 |  |
|  | TPM3 |  |
|  | OSBP |  |
|  | RPRD1B |  |
|  | CAMSAP1 |  |
|  | ANKRD26 |  |
|  | HN1 |  |
|  | PTCD2 |  |
|  | CSNK1E |  |
|  | TBL1XR1 |  |
|  | ZBTB10 |  |
|  | NPM3 |  |
|  | TFCP2 |  |
|  | METTL10 |  |
|  | CLCN2 |  |
|  | DTD1 |  |
|  | C1orf74 |  |
|  | ANAPC5 |  |
|  | ADIPOR2 |  |
|  | CASP6 |  |
|  | OCRL |  |
|  | CARS |  |
|  | DLEU2 |  |
|  | SMARCA1 |  |
|  | VPS35 |  |
|  | TSC22D2 |  |
|  | NAIF1 |  |
|  | COPS3 |  |
|  | RNF160 |  |
|  | C14orf118 |  |
|  | BAG5 |  |
|  | ZNF643 |  |
|  | RBM4 |  |
|  | FAM169A |  |
|  | NME6 |  |
|  | HSPA13 |  |
|  | TAF6 |  |
|  | SFRS15 |  |
|  | FGD1 |  |
|  | TRRAP |  |
|  | STK35 |  |
|  | RANBP2 |  |
|  | TAOK1 |  |
|  | TEAD1 |  |
|  | PPP1R15B |  |
|  | ATR |  |
|  | THAP1 |  |
|  | VPS25 |  |
|  | GPS1 |  |
|  | RMND5A |  |
|  | MRPL13 |  |
|  | PSMD6 |  |
|  | C20orf117 |  |
|  | CBWD2 |  |
|  | NRBP1 |  |
|  | MRPS7 |  |
|  | ZMYM1 |  |
|  | GTPBP8 |  |
|  | UBA5 |  |
|  | PDHA1 |  |
|  | SRRM1 |  |
|  | MTMR4 |  |
|  | ERLIN1 |  |
|  | ZNF202 |  |
|  | LSM14A |  |
|  | TWF1 |  |
|  | ZCCHC7 |  |
|  | FAM53C |  |
|  | PRKRIR |  |
|  | MED28 |  |
|  | AAGAB |  |
|  | BRWD3 |  |
|  | CNP |  |
|  | MUTED |  |
|  | SUMO1 |  |
|  | SEC23IP |  |
|  | ALG9 |  |
|  | TBCCD1 |  |
|  | SLC25A15 |  |
|  | TDP1 |  |
|  | RB1CC1 |  |
|  | RFT1 |  |
|  | CREB1 |  |
|  | BRMS1L |  |
|  | KDM2B |  |
|  | SEC61A2 |  |
|  | HMGXB3 |  |
|  | CLGN |  |
|  | HDGFRP3 |  |
|  | 7-Mar |  |
|  | TOPORS |  |
|  | CAPZA1 |  |
|  | ATP6V1E2 |  |
|  | MFSD9 |  |
|  | PRR3 |  |
|  | PAIP1 |  |
|  | PSMD3 |  |
|  | RAB35 |  |
|  | SAC3D1 |  |
|  | COQ5 |  |
|  | BRD7 |  |
|  | CCNT1 |  |
|  | MSL3L2 |  |
|  | SLC30A9 |  |
|  | GTF2A1 |  |
|  | C19orf40 |  |
|  | THOC1 |  |
|  | C11orf30 |  |
|  | ICK |  |
|  | FAM60A |  |
|  | TBCA |  |
|  | HK2 |  |
|  | TNIP2 |  |
|  | ALG10 |  |
|  | LRCH3 |  |
|  | WAPAL |  |
|  | TRIB3 |  |
|  | ROCK2 |  |
|  | ATP6V0A2 |  |
|  | C5orf51 |  |
|  | TGFBRAP1 |  |
|  | DAXX |  |
|  | DLG5 |  |
|  | PRCC |  |
|  | XRCC6 |  |
|  | ARIH2 |  |
|  | SUPT3H |  |
|  | LOC728640 |  |
|  | NME2 |  |
|  | ZNF777 |  |
|  | ARID3B |  |
|  | ACVR2B |  |
|  | PHF13 |  |
|  | METTL4 |  |
|  | YTHDC1 |  |
|  | AGMAT |  |
|  | AARS |  |
|  | PDF |  |
|  | PRKRA |  |
|  | ARF6 |  |
|  | LRRC37B2 |  |
|  | ASXL2 |  |
|  | VTI1A |  |
|  | GTF3C5 |  |
|  | DUSP14 |  |
|  | TXNL4A |  |
|  | ZNF664 |  |
|  | ICMT |  |
|  | SEC24B |  |
|  | TMEM38B |  |
|  | ZNF507 |  |
|  | CCDC55 |  |
|  | ZNF143 |  |
|  | CENPP |  |
|  | ZNF749 |  |
|  | PACRGL |  |
|  | RNF168 |  |
|  | CLTC |  |
|  | RTTN |  |
|  | MRPL2 |  |
|  | SNAP47 |  |
|  | RHOBTB3 |  |
|  | MTERF |  |
|  | HIC2 |  |
|  | PSMD5 |  |
|  | ENTPD7 |  |
|  | PAQR3 |  |
|  | ZNF480 |  |
|  | SLC25A39 |  |
|  | PSMA5 |  |
|  | THUMPD3 |  |
|  | GBE1 |  |
|  | FCF1 |  |
|  | PTGES2 |  |
|  | ZMYM2 |  |
|  | PLA2G12A |  |
|  | ZNF8 |  |
|  | ZNF318 |  |
|  | C7orf70 |  |
|  | SSR3 |  |
|  | SAMD8 |  |
|  | NT5DC3 |  |
|  | ELK1 |  |
|  | ZNF300 |  |
|  | DUT |  |
|  | PHF14 |  |
|  | PFDN2 |  |
|  | ZNF48 |  |
|  | ALDH18A1 |  |
|  | AVEN |  |
|  | MCFD2 |  |
|  | MRPL9 |  |
|  | LRFN4 |  |
|  | GGH |  |
|  | VPS54 |  |
|  | MAP3K7 |  |
|  | WRAP53 |  |
|  | UBE3A |  |
|  | LOC728554 |  |
|  | TAF5L |  |
|  | UBL4A |  |
|  | BNIP3 |  |
|  | ZNF696 |  |
|  | THOC3 |  |
|  | BEND6 |  |
|  | SNRPB |  |
|  | LCORL |  |
|  | NOL8 |  |
|  | KDM2A |  |
|  | FAM193A |  |
|  | CECR5 |  |
|  | FOXRED1 |  |
|  | PANK3 |  |
|  | DIABLO |  |
|  | POLR3F |  |
|  | EIF3I |  |
|  | SAMD4B |  |
|  | SDHB |  |
|  | YDJC |  |
|  | C3orf17 |  |
|  | EXOSC8 |  |
|  | TSPAN5 |  |
|  | PIPSL |  |
|  | CUL1 |  |
|  | TMEM65 |  |
|  | LOC729082 |  |
|  | TAF13 |  |
|  | CIZ1 |  |
|  | ARHGAP21 |  |
|  | IMPDH2 |  |
|  | C1orf55 |  |
|  | CCDC77 |  |
|  | CORO1C |  |
|  | SIKE1 |  |
|  | EIF2B4 |  |
|  | EYA3 |  |
|  | PGK1 |  |
|  | SOS1 |  |
|  | KATNA1 |  |
|  | FKBPL |  |
|  | PPIA |  |
|  | ZNF699 |  |
|  | TROVE2 |  |
|  | C16orf61 |  |
|  | SGK196 |  |
|  | HEATR3 |  |
