# Supplementary material for: Dysregulation of Pseudogenes/lncRNA-Hsa-miR-1-3p-PAICS Pathway Promotes the Development of NSCLC
Source: J Oncol. 2022 Aug 30;2022:4714931. doi: 10.1155/2022/4714931 (PMC9448537; doi:10.1155/2022/4714931)
Supplement: Supplementary Materials — Table S1. The significant DEGs between normal samples and NSCLC. Table S2. The co-expressed genes of SPOCK2 from UALCAN and GEPIA databases. Table S3. The potential upstream lncRNAs of hsa-miR-1-3p predicted by ENCOLI, miRNet and LncACTdb databases. [file 4714931.f1.zip › 4714931.f1/Supplementary Table S3.docx]

**Supplementary Table S3. The potential upstream lncRNAs of hsa-miR-1-3p predicted by ENCOLI, miRNet and LncACTdb databases.**

| ENCORI | miRNet | LncACTdb |
| --- | --- | --- |
| AL050343.1 | CCDC18-AS1 | MIR4435-2HG |
| CCDC18-AS1 | LAMTOR5-AS1 | LINC00174 |
| AC093157.1 | LAMTOR5-AS1 | LINC00482 |
| AL355310.1 | LINC00622 | AC084219.4 |
| LAMTOR5-AS1 | MIR29B2CHG | C1orf132 |
| LAMTOR5-AS1 | CYTOR | C1RL-AS1 |
| LINC00622 | CYTOR | UCA1 |
| AC234582.1 | MIR4435-2HG | DHRS4-AS1 |
| AL162431.2 | MIR4435-2HG | CYTOR |
| MIR29B2CHG | ACAP2-IT1 | NUTM2A-AS1 |
| CYTOR | STX18-AS1 | CCDC18-AS1 |
| AC133644.3 | MIR4453HG | SNHG14 |
| CYTOR | FAM198B-AS1 | SMIM25 |
| AC133644.3 | LINC00242 | LAMTOR5-AS1 |
| MIR4435-2HG | LINC00174 | RP5-1160K1.3 |
| MIR4435-2HG | LINC-PINT | NUTM2B-AS1 |
| AC137630.4 | LINC-PINT | FAM225B |
| AC007620.2 | OTUD6B-AS1 | MIAT |
| ACAP2-IT1 | RMRP | RBM26-AS1 |
| STX18-AS1 | FAM225B | HOTAIR |
| AC107068.1 | FAM225A | ACAP2-IT1 |
| AC104791.2 | LINC00707 | RP11-263K19.4 |
| MIR4453HG | LINC01518 | FAM225A |
| FAM198B-AS1 | NUTM2B-AS1 | LINC-PINT |
| AC025437.5 | NUTM2A-AS1 | CTD-2619J13.14 |
| LINC00242 | DNMBP-AS1 | RP11-421L21.3 |
| AC092171.5 | RPARP-AS1 | AC012146.7 |
| LINC00174 | NEAT1 | SDCBP2-AS1 |
| AC078846.1 | NEAT1 | AF127936.9 |
| LINC-PINT | NEAT1 | COX10-AS1 |
| LINC-PINT | NEAT1 | NR2F1-AS1 |
| AC055854.1 | NEAT1 | LINC00707 |
| OTUD6B-AS1 | MALAT1 | AC007620.3 |
| RMRP | MALAT1 | RP11-46A10.5 |
| FAM225B | SENCR | RP11-506M13.3 |
| FAM225A | C1RL-AS1 | NEAT1 |
| AL162727.3 | HOTAIR | UBL7-AS1 |
| AL596244.1 | LINC00641 | MALAT1 |
| LINC00707 | DHRS4-AS1 | RP11-459E5.1 |
| LINC01518 | VASH1-AS1 | RP11-282K24.3 |
| AC010997.5 | SNHG14 | OTUD6B-AS1 |
| AL132656.2 | SNHG14 | CTC-436K13.5 |
| NUTM2B-AS1 | SNHG14 | SENCR |
| NUTM2A-AS1 | TMEM202-AS1 | RP11-488C13.5 |
| DNMBP-AS1 | UBL7-AS1 | LINC00641 |
| RPARP-AS1 | LINC01197 | RP11-121C2.2 |
| NEAT1 | COX10-AS1 | LINC00261 |
| NEAT1 | MAPT-IT1 | CTD-2574D22.4 |
| NEAT1 | SOX9-AS1 | CTD-2542L18.1 |
| NEAT1 | LINC00482 | LINC00622 |
| NEAT1 | UCA1 | RP11-701H24.4 |
| MALAT1 | SDCBP2-AS1 | RP11-1007O24.3 |
| MALAT1 | LINC00261 | AC135048.13 |
| SENCR | SMIM25 | RP11-244O19.1 |
| C1RL-AS1 | MIAT | LA16c-313D11.12 |
| AC008115.4 |  | RP11-96D1.10 |
| AC092828.1 |  | RP11-498C9.15 |
| HOTAIR |  | RP11-180P8.3 |
| AC026367.2 |  | RP4-657D16.3 |
| AC127070.4 |  | CTD-2017D11.1 |
| AC127070.4 |  | DKFZP434I0714 |
| AC127070.4 |  | RPARP-AS1 |
| AC127070.4 |  | RMRP |
| AC127070.4 |  | RP11-932O9.10 |
| AC127070.4 |  | RP11-1275H24.3 |
| LINC00641 |  | RP13-131K19.7 |
| DHRS4-AS1 |  | RP11-448A19.1 |
| AL359220.1 |  | RP4-568C11.4 |
| VASH1-AS1 |  | RP11-131L12.4 |
| AL357093.2 |  | RP11-180M15.6 |
| AL928654.4 |  | AC142472.6 |
| SNHG14 |  | LINC00869 |
| AC124312.3 |  | RP11-9E17.1 |
| SNHG14 |  | RP11-147L13.11 |
| AC124312.3 |  | RP11-342K2.1 |
| SNHG14 |  | RP11-181K12.1 |
| AC091057.3 |  | RP11-521B24.4 |
| TMEM202-AS1 |  | RP11-609N14.4 |
| UBL7-AS1 |  | MAPT-IT1 |
| LINC01197 |  | RP5-1014D13.2 |
| Z92544.2 |  | RP11-855A2.2 |
| AC131649.2 |  | CTC-260E6.4 |
| AC120114.2 |  |  |
| AC135048.1 |  |  |
| AC020978.7 |  |  |
| AC092127.1 |  |  |
| AC012146.1 |  |  |
| COX10-AS1 |  |  |
| AC142472.1 |  |  |
| MAPT-IT1 |  |  |
| AC018628.1 |  |  |
| AC005972.1 |  |  |
| AC134407.3 |  |  |
| AC005332.7 |  |  |
| SOX9-AS1 |  |  |
| LINC00482 |  |  |
| AC145207.5 |  |  |
| AC007996.1 |  |  |
| UCA1 |  |  |
| AC011447.7 |  |  |
| AC092279.1 |  |  |
| AC021092.1 |  |  |
| AC012313.1 |  |  |
| SDCBP2-AS1 |  |  |
| LINC00261 |  |  |
| AL035661.1 |  |  |
| SMIM25 |  |  |
| AP000553.1 |  |  |
| MIAT |  |  |
| AL022311.1 |  |  |
